# Phenotypic and Molecular Alterations in the Mammary Tissue of R-Spondin1 Knock-Out Mice during Pregnancy

**DOI:** 10.1371/journal.pone.0162566

**Published:** 2016-09-09

**Authors:** Sead Chadi, Jacqueline Polyte, Lucas Lefevre, Johan Castille, Aude Ehanno, Johann Laubier, Florence Jaffrézic, Fabienne Le Provost

**Affiliations:** GABI, INRA, AgroParisTech, Université Paris-Saclay, Jouy-en-Josas, France; National Cancer Center, JAPAN

## Abstract

R-spondin1 (Rspo1) is a member of a secreted protein family which has pleiotropic functions in development and stem cell growth. *Rspo1* knock-out mice are sex-reversed, but some remain sub-fertile, so they fail to nurse their pups. A lack of *Rspo1* expression in the mammary gland results in an absence of duct side-branching development and defective alveolar formation. The aim of this study was to characterize the phenotypic and molecular alterations of mammary gland due to *Rspo1* knock-out. Using the transcriptional profiling of mammary tissues, we identified misregulated genes in the mammary gland of *Rspo1* knock-out mice during pregnancy. A stronger expression of mesenchymal markers was observed, without modifications to the structure of mammary epithelial tissue. Mammary epithelial cell immunohistochemical analysis revealed a persistence of virgin markers, which signify a delay in cell differentiation. Moreover, serial transplantation experiments showed that Rspo1 is associated with a regenerative potential of mammary epithelial cell control. Our finding also highlights the negatively regulated expression of Rspo1’s partners, *Lgr4* and *RNF43*, in the mammary gland during pregnancy. Moreover, we offer evidence that Tgf-β signalling is modified in the absence of Rspo1. Taken together, our results show an abrupt halt or delay to mammary development during pregnancy due to the loss of a further differentiated function.

## Introduction

The *R-spondin1* (*Rspo1*) gene belongs to a family which encodes secreted proteins (Rspo1-Rspo4) that are widely expressed in vertebrate embryos and adults, and have pleiotropic functions in development and stem cell growth [1–5]. The *in vivo* functions of Rspo1 have been unravelled by means of genetic studies in humans and mice. Mutations in the human *RSPO1* gene were identified in individuals with female to male (XX) sex reversal exhibiting XX true hermaphroditism [6, 7]. Loss of the *Rspo1* gene in XX mice causes masculinized ovaries, with epididymis and vas deferens-like structures, rather than a complete phenotypic male conversion [8, 9]. *Rspo1* null foetal ovaries display oocyte depletion. *Rspo1* null female mice have extremely poor fertility; interestingly, however, even when they are able to produce offspring they are subsequently unable to feed their pups [10].

A functional link between Rspo and Wnt signalling was established from the ability of Rspo to enhance β-catenin signalling [(for a review, see [2, 4]). Leucine-rich repeat-containing G-protein-coupled receptors (Lgr) 4/5/6 may interact physically with low-density lipoprotein receptor-related protein (Lrp) 5/6 after Rspo recognition, and activate Wnt/β-catenin signalling [11–16]. Rspo proteins are also able to promote Wnt/β-catenin signalling by stabilizing the Frizzled and Lrp5/6 receptors. Zinc and RING finger 3 (Znrf3) and its homologue RING finger 43 (Rnf43) are two recently discovered transmembrane E3 ubiquitin ligases that promote the turnover of Frizzled and Lrp6 receptors on the cell surface [17]. Data have shown that RSPO1 induces the clearance of Znrf3 from the membrane by interacting with the extracellular domains of Lgr4 and Znrf3, which stabilize the Frizzled and Lrp6 receptors in order to enhance Wnt/β-catenin signalling [18]. In addition, Rspo proteins also regulate non-canonical Wnt pathways [19, 20]. The Xenopus Rspo3 protein activates Wnt/PCP signalling in cooperation with Wnt5A by promoting syndecan4-mediated Fzd7/Wnt5A complex internalization [19]. Because a direct interaction between Rspo3 and Fzd7 may not exist, it remains unclear how the Rspo protein imposes its activity on Fzd7. Recently, Carmon *et al*. [20] showed that the intracellular scaffold protein IQ motif containing GTPase-activating protein 1 (IQGAP1) is an Lgr4-interacting protein that mediates the interaction between Rspo-Lgr4 and the Wnt pathway, and potentiates β-catenin-independent signalling by regulating actin dynamics.

Unlike most other organs, most of the development of the mammary gland occurs after birth (reviewed [21–25]). At birth, the mammary gland is made up of a small mammary ductal tree with 15–20 branches. At puberty, the primary ducts extend into the fat pad tissue. Early in pregnancy, the mammary epithelial ducts form side branches that serve as ductules for the alveolar structures of differentiated mammary epithelial cells (MEC). During the second part of pregnancy, these alveolar structures differentiate and become the sites for milk production. Physiological changes that affect the mid-pregnancy gland lead to a reduction in cell proliferation and an increase in differentiation parameters, while other mechanisms inhibit the activation of secretion. Epithelial compartment composed by ductal and lobular structures contain luminal cells which form a layer of cells surrounding a central lumen. The luminal cells are surrounded by a basal layer of cells, containing myoepithelial and stem/progenitor cells, which rest on a “basement membrane” composed of extracellular matrix components permit to separate the parenchymal and stromal compartments. These different processes are coordinated by multiple signalling pathways [(for a review, see [23, 26–28]).

Our team has shown that mammary epithelial *Rspo1* expression is required for normal mammary gland development in the mouse [10]. In order to investigate the role of Rspo1 in the mammary epithelium, its loss-of-function phenotype was studied by transplanting the mammary epithelium of *Rspo1*^-/-^ animals and their WT litter-mates. Because the *Rspo1*^-/-^ females were sub-fertile [9, 10], this approach enabled an assessment of mammary epithelial development during pregnancy without the potential secondary effects of *Rspo1* deletion in other tissues, thus ensuring that *Rspo1*^-/-^ and WT mammary tissues were subject to the same hormonal environment.

The defective mammary ductal system formation and side-branching development that occurs in nulliparous *Rspo1*^-/-^ mice was also observed in transplanted samples at mid and late pregnancy (day-12 and day-16, [10]). In late pregnancy, a marked defect to alveolar development was observed in transplanted *Rspo1*^-/-^ mammary epithelium samples [10]. Rspo1 is essential for mammary ductal invasion, branching and alveologenesis.

The principal objective of the work reported here was to clarify the molecular functions that are altered during pregnancy in the mammary gland in the absence of *Rspo1* expression.

## Materials and Methods

### Animals and tissue collection

The origin of *Rspo1*^-/-^ mice has already been described [8]. The mouse line was kept on a FVN/B background. The animals were kept at a temperature of 21°C at 55% humidity under a 12 h/12 h light/dark cycle with free access to food and water. The day of vaginal plug appearance was counted as day-0 of pregnancy. All experiments involving animals were performed in strict accordance with the guidelines of the Code for Methods and Welfare Considerations in Behavioural Research with Animals (Directive 86/609EC) and the recommendations of the French Commission de Génie Génétique (Permit # 12931 (01.06.2003)) which approved this study. Every effort was made to minimize animal suffering. For the surgical procedure, the animals received an analgesic by intraperitoneal (i.p.) injection of finadyne (2 mg/kg), 20 min before anaesthesia. Then they were anaesthetized by the inhalation of 2.5% isofluorane.

### Transplantation of mammary epithelium

Mammary epithelium transplant experiments were performed as previously described [29, 30]. Briefly, the proximal part of the inguinal gland of 3-week-old athymic NCr-nu/nu mice containing the mammary epithelium was excised (cleared fat pad). Small pieces of mammary tissue collected from the nulliparous female mice were grafted into the cleared fat pad of the host mice. For serial mammary epithelium transplant experiments, eleven weeks after transplantation, the tissues were harvested from nulliparous animals and used for a further transplantation or collected to evaluate the percentage at which each outgrowth filled the host fat pad. Three independent serial transplant experiments were performed.

### RNA isolation

Tissues were excised from the animals and processed immediately for total RNA extraction using the RNeasy Lipid Tissue kit (Qiagen) or RNA Now reagent (Ozyme), as described by the manufacturers. The quantity and quality of RNA were assessed using an Agilent BioAnalyzer.

### Microarray analysis

100 ng of total RNA were labelled in accordance with the manufacturer’s protocols and hybridized to the Affymetrix ^®^ Mouse Gene 1.1 ST Array, representing 28,000 well-annotated genes with more than 770,000 distinct probe sets. This analysis was performed at the Affymetrix Platform at Institut Curie, Paris. Experiments were performed with RNA extracted from the transplanted mammary glands of eight mice at pregnancy day-12 (four wild-type (WT) and four *Rspo1*^-/-^ samples) and of eight other mice at pregnancy day-16 (four WT and four *Rspo1*^-/-^ samples).

### Quantitative PCR analysis

mRNA quantifications were performed by the reverse transcription (RT) of 5 μg total RNA using the Superscript First Strand Synthesis System II (Invitrogen), according to the manufacturer’s instructions. Quantitative PCR (qPCR) was performed on RT products using the Mastercycler ep Realplex (Eppendorf). The reaction conditions consisted of 15 min. at 95°C (1 cycle), 15 s at 95°C and 60 s at 60°C (45 cycles) with primers (10 μM) using Absolute QPCR SybrGreen (Thermo Scientific). The primer sequences are presented in S1 Table. Each stage was analysed in triplicate in three *Rspo1*^-/-^ and three WT animals. After normalization using the *Cpr2* or *Gapdh* housekeeping gene expression levels were compared between *Rspo1*^-/-^ and WT samples using the Delta-Delta Ct method (2^-Ct^).

### Immunohistochemistry analysis

For immunohistological analysis, dissected mammary glands were fixed in RCL2 (Alphelys) and embedded in paraffin. Paraffin sections (5 μm) were used for the experiments. Heat-induced retrieval was performed by microwaving sections in 10 mM sodium citrate at pH 6.0 for 10 min. After blocking in 0.05% foetal bovine serum (Lonza), the sections were incubated overnight at 4°C with primary antibodies (S2 Table), followed by incubation for 1h at room temperature with secondary antibody (S2 Table), then counterstained with Vectashield-DAPI medium (Vectorlabs). The rabbit polyclonal antibody recognizing Nkcc1 was obtained from Dr. Jim Turner (NIDCR, NIH, Bethesda, MD). Immunofluorescence was viewed under a Leica Leitz DMRB microscope. For Ki67 quantification, the slides were scanned on a Pannoramic Scan (3D Histech). Each section image was divided into multiple images of 1 mm^2^ format using Case viewer and Pannoramic viewer software systems. The quantification of labelled cells was performed using ImageJ software (RSB) on at least four independent 1 mm^2^ squares per animal. Each immunohistochemical analysis was performed on three *Rspo1*^-/-^ and three WT animals. The specificity of the immunolabelling technique was assessed by incubating the slides with a secondary antibody alone (data not shown).

### Statistical analysis

Data on the differences between *Rspo1*^-/-^ and WT samples in terms of the *Axin2* expression obtained by qPCR, and on the cell proliferation obtained by Ki67 immunostaining, were compared using one-way analysis of variance (ANOVA). Standard Error of the Mean (SEM) values were calculated for each group. A p-value of 0.05 was considered to be statistically significant. The microarray data were preprocessed using Robust Multi-array Average (RMA) in the default configuration for background adjustment and normalization. Analyses were performed using BioConductor version 2.10 [31] and R version 2.15.0 [32]. To identify genes that were differentially expressed, empirical Bayesian moderated *t*-statistics implemented under the BioConductor LIMMA package (version 3.12.0) [33] were applied. P-values were adjusted for multiple testing using the Benjamini and Hochberg method [34].

## Results

### Transcript profiling revealed gene expression affected in the *Rspo1*^-/-^ mammary epithelium during pregnancy

To identify the molecular functions affected by *Rspo1* knock-out in MEC transcriptional profiling, analyses were performed on transplanted mammary fat pads using the Affymetrix Mouse Gene 1.1^ST^ array at two stages of pregnancy: mid-pregnancy (day-12) corresponding to the extensive mammary epithelial cell proliferation stage, and late pregnancy (day-16) corresponding to a stage characterized by fully differentiated mammary epithelial cells present in lobulo-alveolar structures. RNA samples were prepared at each stage from four transplanted mice within each genotype (*Rspo1*^-/-^ and WT) and hybridized individually. On day-12 of pregnancy, statistical analysis of these arrays led to the identification of 246 differentially expressed genes based on mRNA accessions in the *Rspo1*^-/-^ samples as compared to WT samples (adjusted p-value < 0.05 and fold change ≤ -2 or ≥ +2). One hundred and forty genes were down-regulated in *Rspo1*^-/-^ *versus* the WT mammary epithelium, and 106 genes were up-regulated (S3 Table). On day-16 of pregnancy, statistical analysis showed that 1,690 differentially expressed genes were identified in *Rspo1*^-/-^ samples compared to WT samples (adjusted p-value < 0.05 and fold change ≤ -2 or ≥ +2). Five hundred and eighteen genes were down-regulated and 1,172 genes up-regulated (S4 Table). Among the genes that were misregulated, 213 were also misregulated on day-12 of pregnancy, representing 86.6% of misregulated genes at this stage. All genes were misregulated in the same way and in most cases the fold change values were higher on day-16 than on day-12 of pregnancy. To validate the microarray data, ten differentially expressed genes were analysed using RT-qPCR. The expression pattern obtained by RT-qPCR was consistent with the results of the microarray technique (S5 Table).

On day-12 of pregnancy, Ingenuity Pathway Analysis (IPA) (http://www.ingenuity.com/) was used to assess the functions associated with *Rspo1* knock-out (S6 Table). Twelve networks were significantly enriched; they were related to organ development and function and cellular function and maintenance (such as “cellular movement” or “cell death and survival”).

Functions highlighted on day-16 of pregnancy were also those most significantly modified on day-12 of pregnancy (S7 Table). However, networks linked to “cell-to-cell signalling and interaction” and functions related to differentiated MEC such as “lipid metabolism” and “small molecule biochemistry”, were specifically modified at pregnancy day-16.

### Mammary epithelial tissue characterization in the absence of Rspo1

The microarray analysis showed that, in the absence of Rspo1, genes coding for proteins which play key roles in cell-cell interactions (*Gjb6*, *Gjb2*, *Duox1* and *Duoxa1*) and in the composition of the extracellular matrix (*Chrdl2* or *Bnf-1*) were amongst the most down-regulated genes at mid- and late-pregnancy (Table 1, S3 and S4 Tables). Changes to the morphology of mammary tissue were therefore investigated. Tight junctions, cellular structures that facilitate cell-cell communications, are important to maintaining the three-dimensional structure of the mammary epithelium [35]. By immune-labelling the protein constituents of tight junctions, Occludin and Zo-1, as well as the luminal and myoepithelial cell markers, E-cadherin and smooth muscle actin (α-SMA), we were able to show that the structure of the mammary epithelium was not modified in *Rspo1*^-/-^ mammary samples during pregnancy (S1 Fig).

**Table 1:** Deregulated genes in *Rspo1*^-/-^ *versus* WT mammary epithelium on days 12 (P12) and 16 (P16) of pregnancy, in fold change, following Affymetrix Mouse Gene 1.1^st^ array analyses.

		P12	P16
Cell-cell interaction	***Gjb6***	-31.49	-44.83
	***Gjb2***	-6.09	-9.34
	***Duox1***	-3.79	-7.95
	***Duoxa1***	-5.83	-6.79
Extracellular matrix	***Chrdl2 (Bnf-1)***	-26.62	-26.22
Mesenchyme marker	***Ncad (Cdh2)***	+2.59	+2.44
	***Snail1***	+1.71	+2.39
	***Msn***	+1.59	+2.13
Differentiated MEC	***Wap***	-18.37	-6.33
	***Folr1***	-3.73	-5.53
	***Csn1s2b***	-2.47	-18.37
	***Lalba***	ND	-5.63
MEC differentiation	***Fabp3***	-27.69	-11.02
	***Bhlha15***	-6.78	-7.26
Mammary gland development	***Ptn***	+3.39	+2.38
Lipid synthesis	***Acsl4***	-2.68	-4.25
	***Acss1***	-1.80	-2.48
Mammary fat globule formation	***Xdh***	-1.91	-2.57
Luminal progenitor marker	***Elf5***	-2.01	-2.30
Duct development	***FoxA1***	+3.46	+3.79
	***Cited1***	+3.28	+3.67
Rspo partner	***Lgr4Rnf43***	ND-1.93	-4.92–2.76
	***Fzd4***	ND	+2.85
Tgf-β/Slit/Robo pathway	***Robo1***	ND	+2.20
	***Robo2***	ND	+2.46
	***Slit2***	+3.08	+4.18

During mammary gland development, mammary tissue undergoes extensive remodelling in the context of epithelial to mesenchymal transition (EMT), and its reversion via the mesenchyme to epithelial transition (MET) [36, 37]. In *Rspo1*^-/-^ samples, the *Ncad* gene, a marker of the mesenchyme, was over-expressed, at mid-pregnancy, while in late-pregnancy several genes that characterize mesenchymal tissue (*Ncad*, *Snail1* and *Msn*, Table 1) were over-expressed. These data could reflect an MET defect and an abnormal persistence of mesenchymal tissue or a delay to development of the gland or a greater proportion of stromal elements in the absence of Rspo1.

### MEC characterization in the absence of Rspo1

**Fig 1:**
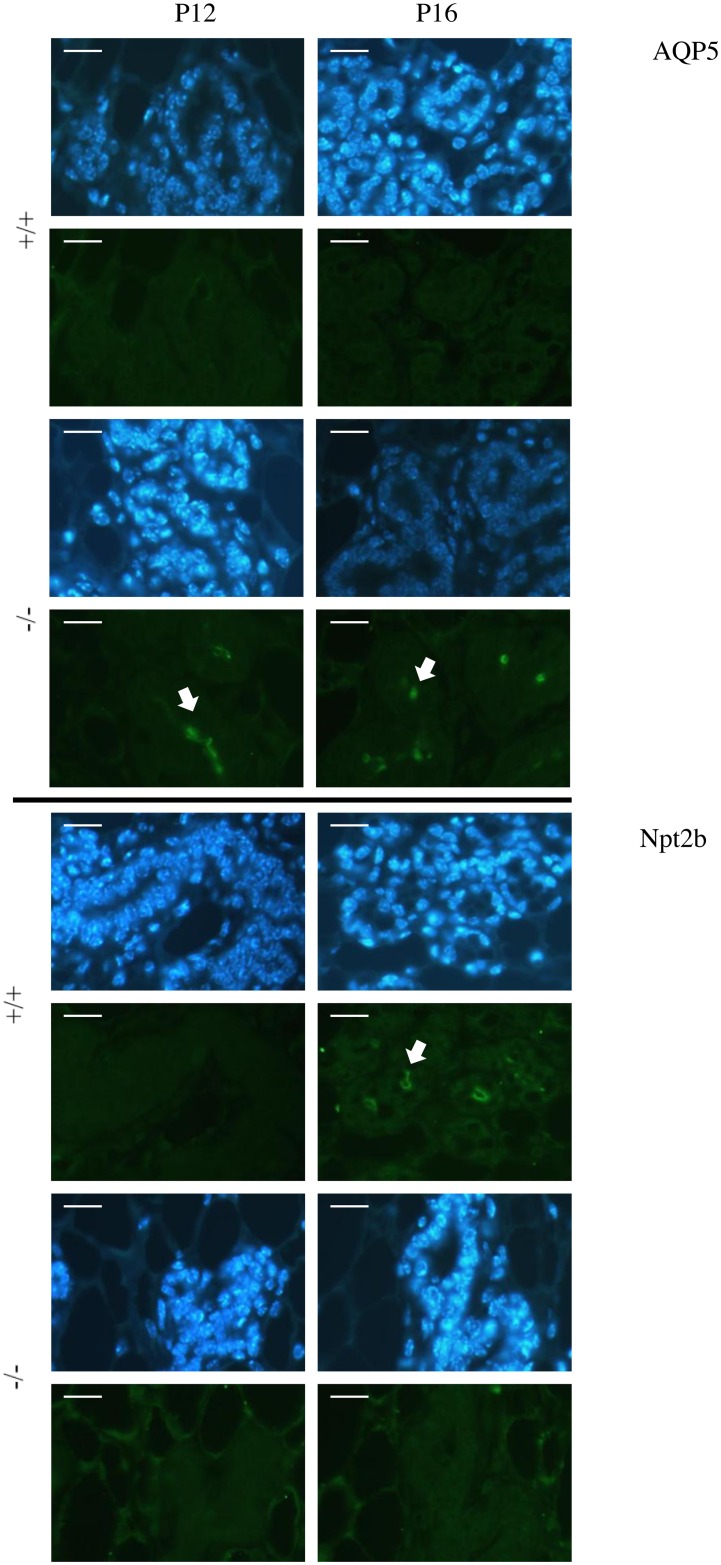
Defects of mammary epithelium development and differentiation on days 12 and 16 of pregnancy in *Rspo1*^-/-^ mice. Immunostaining of Aqp5, a marker of ductal epithelial cells at the virgin stage, and Npt2b, a marker of secretory MEC, in representative *Rspo1*^-/-^ (-/-, N = 3) and WT (+/+, N = 3) mammary tissues during pregnancy (day-12 (P12) and day-16 (P16)). Nuclei were stained with DAPI (blue). Scale bars correspond to 12.5 μm.

Although the general aspect of mammary epithelial tissue was not altered in *Rspo1*^-/-^ mice, further characterizations of MEC were performed in order to understand the role of Rspo1. The differentiated status of luminal cells was investigated using markers specific to epithelial cell identity [38] such as Nkcc1, a marker of ductal epithelial cells, and Aqp5, a marker of the apical membrane of ductal epithelial cells during the virgin stage. Immunohistochemical analysis did not reveal any difference between *Rspo1*^-/-^ and WT animals with respect to Nkcc1: as expected, the Nkcc1 protein was present in the basal membrane of ductal epithelial cells on days 12 and 16 of pregnancy (data not shown). However, Aqp5 was detected in the *Rspo1*^-/-^ mammary epithelium, but not in the WT mammary epithelium, at both days 12 and 16 of pregnancy (Fig 1).

Microarray analysis revealed that, unlike the WT samples, the most down-regulated gene in *Rspo1*^-/-^ samples, was *Gjb6* (also known as *connexin30*), which is involved in initiating or maintaining alveolar development (Table 1; S3 and S4 Tables, [39]). Moreover, on day-12 of pregnancy, genes coding for proteins characteristic of differentiated MEC (such as *Wap*, *Folr1*), or required for MEC differentiation (such as *Fabp3*, *Bhlha15*), as well as those involved in mammary gland development (such as *Ptn*) were amongst the most down-regulated genes (Table 1 and S3 Table). In late-pregnancy (day-16), major genes involved in the synthesis of lipids (*Acsl4*, *Acss1*) and mammary fat globule formation (*Xdh*), as well as genes coding for milk proteins (such as *Csn1s2b*, *Wap*, *Lalba*) and all functions characterising differentiated luminal cells, were down-regulated (Table 1). However, the expression of MEC markers, such as *Krt8*, *Krt18* or *Cdh1*, was not misregulated. Moreover, using immunohistochemical analysis, we showed that Npt2b, a marker of the secretory process [38], was present in WT, but not in *Rspo1*^-/-^ samples, on day-16 of pregnancy (Fig 1), thus confirming the persistence of virgin markers and the defect in differentiation markers in the MEC of *Rspo1*^-/-^ mice.

### Characterization of the regenerative potential of mammary epithelium in the absence of Rspo1

**Fig 2:**
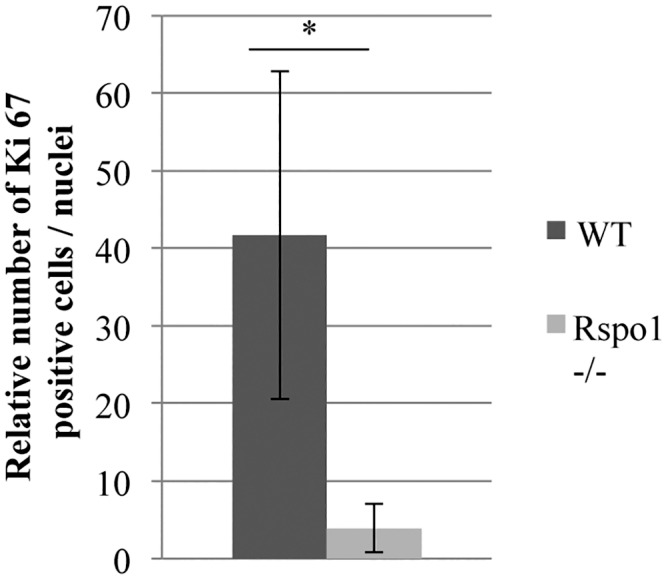
Defective MEC proliferation at mid-pregnancy in the absence of Rspo1. Detection of cell proliferative activity by Ki-67 immunostaining. Data represent mean ± SEM values obtained on day-12 (P12) of pregnancy on paraffin sections of mammary gland from *Rspo1*^-/-^ (N = 3) and WT (N = 3) mice. *: indicates a significant difference (p < 0.05, ANOVA).

Mid-pregnancy is a stage characterized by massive MEC proliferation. In order to investigate whether the absence of Rspo1 impacts the proliferation of MEC, Ki-67 immunostaining experiments were performed. The number of Ki-67 positive cells was significantly smaller in the *Rspo1*^-/-^ group than in WT mice, thus revealing a proliferation defect (Fig 2).

**Fig 3:**
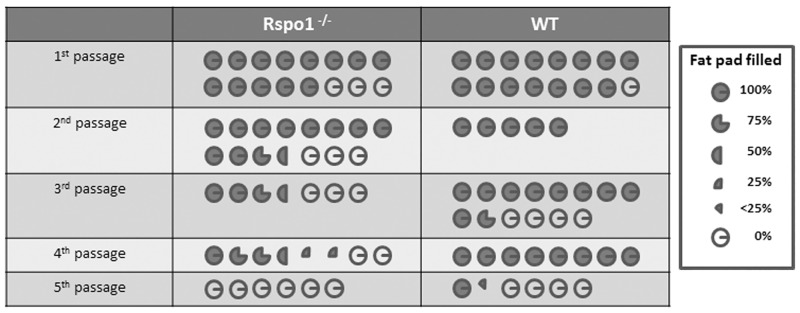
The regenerative potential of *Rspo1*^-/-^ mammary epithelium was affected. A table which summarizes three independent serial transplant experiments with *Rspo1*^-/-^ and WT engrafted gland is represented. Each engrafted gland is represented by a micrograph. Dark sectors indicate the area of fat pad filled by engrafted epithelium.

Expression of the *Elf5* transcription factor, which regulates mammary gland stem cell activity [40], was down-regulated in the *Rspo1*^-/-^ group (Table 1; S3 and S4 Tables). Moreover, the regenerative potential of the *Rspo1*^-/-^ mammary cell population was evaluated by serial transplantation experiments which enabled assay of the mammary regeneration potential of multiple types of stem and progenitor cells. A defect of mammary gland reconstruction was observed in the absence of *Rspo1* (Fig 3).

Taken together, these data suggest a role for Rspo1 in the regenerative potential of mammary epithelial tissue.

### Characterization of signalling pathways in the mammary epithelium in the absence of Rspo1

**Fig 4:**
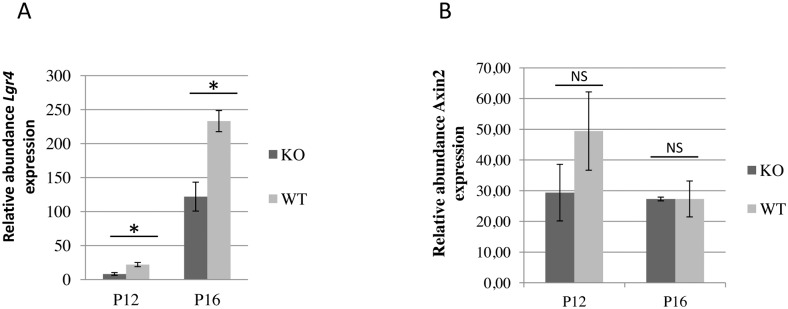
*Lgr4* and *Axin2* transcript levels in mammary epithelium in the absence of Rspo1. Quantification of *Lgr4* (A) and *Axin2* (B) mRNA in *Rspo1*^-/-^ (KO, N = 3 for each stage) and WT (N = 3 for each stage) mammary gland on days 12 (P12) and 16 (P16) of pregnancy determined by RT-qPCR assay. *Lgr4/Cpr2 and Axin2/Cpr2* mRNA ratios were calculated. NS: no significant difference, *: indicate a significant difference (p < 0.05, ANOVA).

The role of Rspo proteins as Wnt agonists enhancing Wnt/β-catenin activation has been described in several tissues [(for a review, see [2]). The involvement of the Wnt signalling pathway in mammary branching has also been well documented [41–44]. However, our microarray data did not reveal any changes in the expression of the classic target genes of this signalling pathway, such as *Axin2*, *Myc*, *CyclinD1* and *Jun*, and the data were confirmed for *Axin2* by RT-qPCR (Fig 4).

To be active, Rspo forms a ligand/receptor complex with various membrane proteins such as the Wnt receptors Frizzled and Lrp6, Kremen, Syndecan4 and Lgr4/5, as well as the membrane E3 ubiquitin ligases Znrf3/Rnf43. Here, the expression of *Lgr4* and *Rnf43* was down-regulated, while that of *Fzd4* was up-regulated (Fig 4 and Table 1, S4 Table).

**Fig 5:**
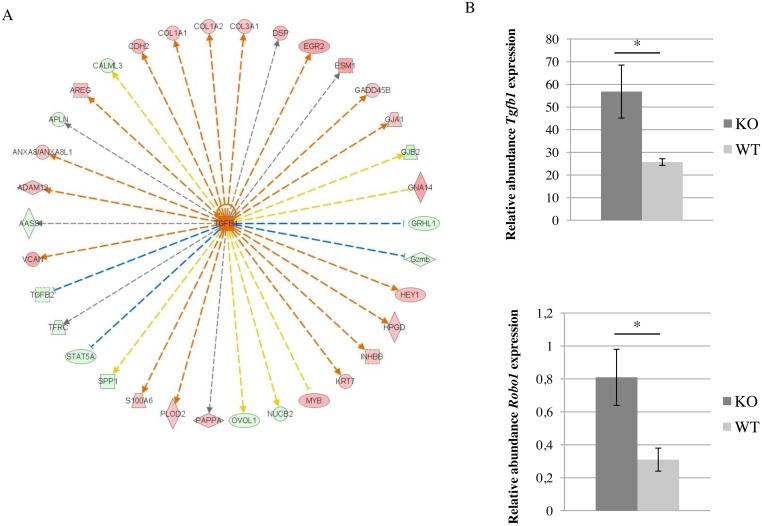
Tgf-β1 network in mammary epithelium in the absence of Rspo1. **A-** Ingenuity Pathway Analysis performed using the 246 differentially expressed genes identified in the absence of Rspo1 on day-12 of pregnancy highlighted the Tgf-β1 network. Green and red colours indicate down and up-regulation, respectively. **B-** *Tgf-β1* and *Robo1* transcript levels were measured using RT-qPCR on mammary gland from *Rspo1*^-/-^ (KO, N = 3 for each stage) and WT (N = 3 for each stage) mice on day-12 of pregnancy. Transcript levels were quantified relative to those of *Gapdh*. *: indicates a significant difference (p < 0.05, ANOVA).

IPA analysis of the microarray data obtained from mammary gland collected on day-12 of pregnancy highlighted the Tgf-β1 network (Fig 5). *Tgf-β1* misregulation was not detected by microarray analysis. However, using RT-qPCR, we were able to show that *Tgf-β1* was more strongly expressed in the *Rspo1*^-/-^ group than in the WT group (Fig 5), as was *Robo1*, which has recently been identified as being regulated by Tgf-β1 [45], thus demonstrating a modification to the Tgf-β1 network in the absence of Rspo1.

## Discussion

Rspo1 is essential for normal mammary gland development. *In vivo*, Chadi *et al*. [10] showed that its absence from MEC affects ductal invasion, branching formation and alveolar formation. A plethora of molecular signals cooperate to ensure mammary morphogenesis through communication between epithelial and stromal cells. This process is set in motion by ovarian and pituitary hormones that can deliver signals to both types of cells through their receptors. In the context of Rspo1^-/-^ mammary epithelial tissue transplantation into WT mice, the phenotype defect was only due to the absence of Rspo1 in the mammary epithelium, thus demonstrating the importance of Rspo1 to MEC functioning. Rspo1 is absent from MEC, an abrupt halt or a delay to mammary development is observed during pregnancy. This phenotype resembles the arrest observed in prolactin- or progesterone-receptor knock-out mice [46]. The changes observed using a microarray approach indicate that this arrest results in a loss of further differentiated function.

The transcriptional profiling confirmed the abnormal maintenance of mesenchymal markers at the two stages of pregnancy. These results could be explained by the deficiency in epithelial side-branching and a greater proportion of stroma. The structure of the Rspo1^-/-^ mammary epithelial tissue present did not appear to be disturbed, but the MEC conserved the unusual characteristics of virgin ductal epithelial cells in the absence of Rspo1, and were less differentiated.

An impact of the absence of Rspo1 on cell proliferation was observed in our model. Down-regulation of the *Elf5* progenitor cell marker gene in Rspo1^-/-^ mammary epithelium, up-regulation of *Slit2* and *Robo2* (Table 1), which are key molecules in mammary stem cell senescence [47] and the data obtained by successive transplantations showing a reduction of regenerative potential in the absence of Rspo1 (Fig 3), could be explained by the presence of a progenitor population that was less abundant in the absence of Rspo1. Moreover, our results are congruent with the decreased repopulating frequency observed under the *Rspo1* knock-down approach developed by Cai *et al*. [48].

The precise mechanism of Rspo signalling remains unclear. Various membrane proteins have been reported to bind to Rspo, including the Wnt receptors Frizzled and Lrp6, Kremen, Syndecan4 and Lgr4/5, as well as the membrane E3 ubiquitin ligases Znrf3 and Rnf43, and several models of Rspo signalling have been proposed. Recently, Hao *et al*. [18] identified two membrane E3 ubiquitin ligases, Znrf3 and Rnf43, that target Wnt receptors for degradation. In the absence of Rspo1, expression of the Rpso1 receptor *Lgr4* and ligase *Rnf43* were down-regulated, and that of Wnt receptor Fzd4 was up-regulated. In the future, it will of major interest to determine whether Rspo1 might be a positive regulator of its own partners, *Lgr4* and *Rnf43* in the MEC, or whether the number of cells expressing its partners is reduced in the absence of Rspo1.

Rspo1 has been described as promoting the Wnt/β-catenin signalling pathway [(for a review, see [2, 4]). Recently Cai *et al*. [48], using a knock-down of *Rspo1* and *Wnt4 in vitro*, showed that Rspo1 and Wnt4 cooperate to promote mammary stem cell self-renewal via the Wnt/β-catenin signalling pathway. *In vivo*, our data showed that the knock-out of *Rspo1* in MEC did not modify the expression of target genes in the Wnt/β-catenin signalling pathway (such as *Myc*, *CyclinD1*, *Jun*, *Axin2*). Our data were congruent with those found by Baljinnyam *et al*. [49], who recently showed that the β-catenin pathway is not involved in the down-regulation of most genes identified as being Rspo2/Wnt targets. Rspo2/Wnt3a signalling regulates the expression of various effectors in several growth factor pathways (Insulin/Igf and Fgf) and that of several transcription factors (Ahr, Helt, Klf5, Tcf4) in C57MG mouse MEC [49]. Here, in the *Rspo1*^-/-^ mammary epithelium, the expressions of these genes were not modified, suggesting that the action of Rspo1 and Rspo2 may be different. However, these differences could also be due to the experimental models used (*in vivo vs*. *in vitro*).

Numerous integrated signalling networks regulate mammary gland morphogenesis; amongst them Tgf-β signalling inhibits proliferation and branching. Macias *et al*. [45] recently proposed a model where SLIT/ROBO1 signalling suppresses mammary branching outgrowth by limiting basal cell numbers, and they identified TGF-β1 as the negative regulator upstream of *Robo1*. In our study, IPA analysis of transcriptomic data highlighted the importance of Tgf-β1 signalling in the absence of Rspo1, at mid-pregnancy. Taken together, these data enable us to propose the hypothesis that in the absence of Rspo1, defective duct side-branching development may be linked to activation of the Tgf-β1/Slit/Robo signalling pathway, which limits basal cell numbers.

## Conclusions

The absence of Rspo1 affects both development of the mammary epithelium and the differentiation of MEC. The changes observed following transcriptomic and immunohistochemistry analyses during pregnancy in absence of Rspo1 in MEC reflected an abrupt halt to, or a delay of, mammary development during pregnancy, which results in a loss of further differentiated function.

## Supporting Information

S1 Fig Immunostaining of E-cadherin (red), Occludin (green), α-Smooth muscle actin (α-SMA, red) and Zo-1 (green) in representative *Rspo1*^-/-^ (-/-, N = 3) and WT (+/+, N = 3) mouse mammary epithelia on day-12 of pregnancy. Nuclei were stained with DAPI (blue). The scale bar corresponds to 12.5 μm.
(TIF)

S1 Table Specific primers used for RT-qPCR analyses. (DOCX)

S2 Table Antibody information. (DOCX)

S3 Table List of deregulated genes in *Rspo1*^-/-^ *versus* WT samples on day-12 of pregnancy. (DOCX)

S4 Table List of deregulated genes in *Rspo1*^-/-^ *versus* WT samples on day-16 of pregnancy. (DOCX)

S5 Table RT-qPCR analysis of 10 genes selected from the microarray data. (DOCX)

S6 Table Ingenuity Pathway Analysis-Top Functions associated with *Rspo1* inactivation on day-12 of pregnancy. (DOCX)

S7 Table Ingenuity Pathway Analysis- Top Functions associated with *Rspo1* inactivation on day-16 of pregnancy. (DOCX)
