# Supplementary figures and images for: Phenotypic and Molecular Alterations in the Mammary Tissue of R-Spondin1 Knock-Out Mice during Pregnancy
Source: PLoS One. 2016 Sep 9;11(9):e0162566. doi: 10.1371/journal.pone.0162566 (PMC5017653; doi:10.1371/journal.pone.0162566)

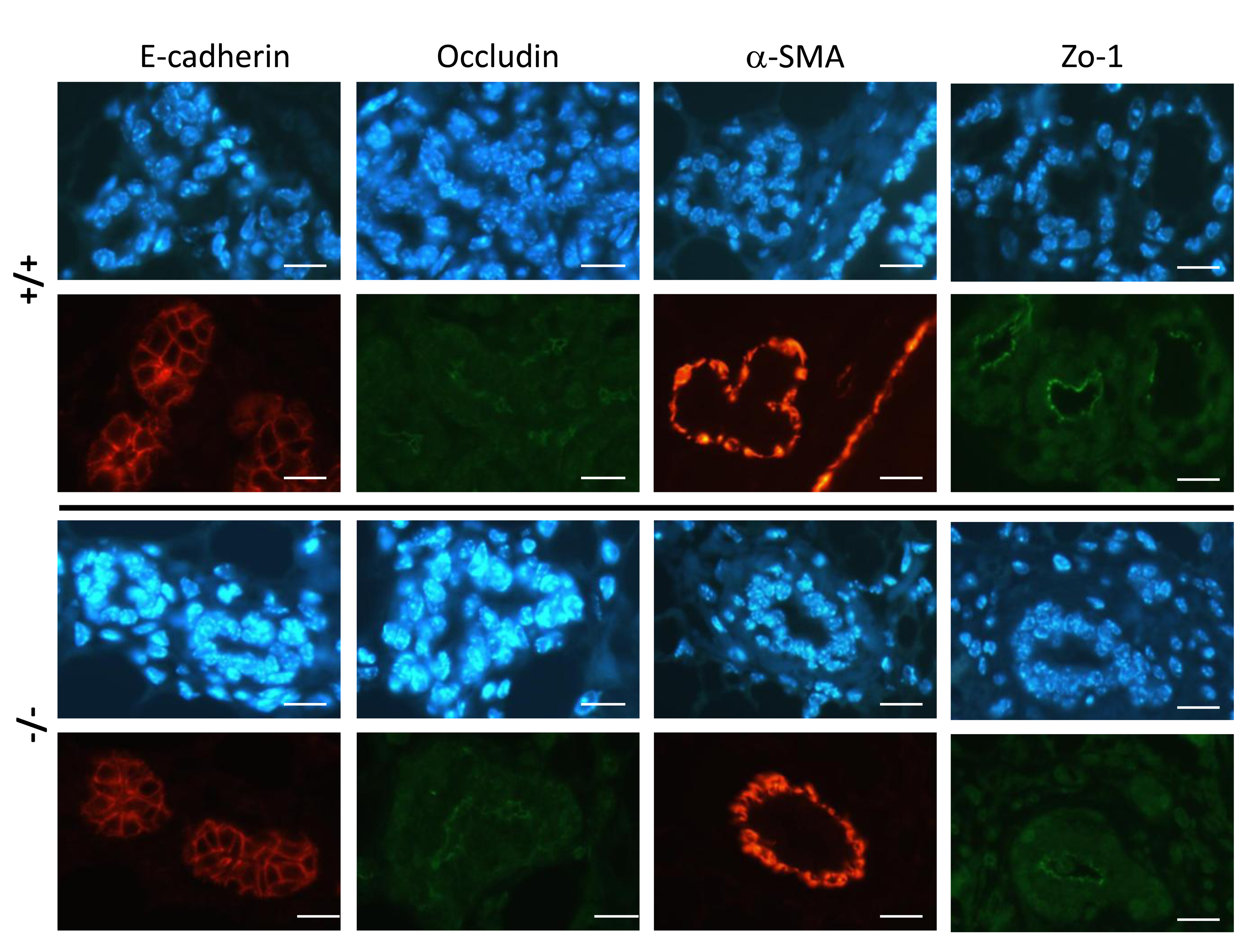

Supplement: S1 Fig — Nuclei were stained with DAPI (blue). The scale bar corresponds to 12.5 μm. (TIF) [file pone.0162566.s001.tif]
