# Supplementary material for: Phenotypic and Molecular Alterations in the Mammary Tissue of R-Spondin1 Knock-Out Mice during Pregnancy
Source: PLoS One. 2016 Sep 9;11(9):e0162566. doi: 10.1371/journal.pone.0162566 (PMC5017653; doi:10.1371/journal.pone.0162566)
Supplement: S1 Table — (DOCX) [file pone.0162566.s002.docx]

**Supplemental Table 1: Specific primers used for RT-qPCR analyses**

|  | **Sequence forward** | **Sequence reverse** |
| --- | --- | --- |
| *Axin2* | AGCGCCAACGACAGCGAGTTA | GGCGGTGGGTTCTCGGAAAA |
| *Csn1s2a* | TAACACGCCCACCCAGGAATCCA | TCTGGGGATGAAGAGCTTGGGGA |
| *Fabp3* | AGTCACTGGTGACGCTGGACG | AGGCAGCATGGTGCTGAGCTG |
| *Gjb6* | TGCAGTGACTCTTGAGCTGGGCG | ACTTCCTGGGCAGCCACCACTA |
| *Igfbp7* | GTGCCACGCATCCAACTCCCAA | GTGTGTCAGGCAAGAGCAGGGT |
| *Lect1* | AGCACAGGTGAAGGCTCGCA | CACGGCCACCCAAATCAGCGA |
| *Lgr4* | ACCCAGTGAAGCCATTCGTGGAC | TGCCGCAACTGAACGAGCCC |
| *Mtmr9* | ATCCTCCGGCAGTTCCCTTGCT | GGCCGATTGACCCATGACCACA |
| *Olah* | TGCCAAGTGGGGCCGAAAGATT | ATGATGGGCAACAGGGCAGTCA |
| *Robo1* | TGGCTCGCCGCCAAATGCAA | TGGCGGGTCTGGCTTTCTGGA |
| *Rspo1* | GGCTCTACAGCCGCTAACAG | CTGCGCACGGTACACTTGC |
| *Sparc* | AAGAAGGGCCACAAGCTCCACC | CACGCATGCGCAGAGGGAATT |
| *Tgfb1* | GCGTGCTAATGGTGGACCGCA | GGGGGTTCGGGCACTGCTTC |
| *Vcan* | AGAGTCCGTGGAAGGCACAGCA | GCCTCCGTTGAGGCATGGGTTT |
