# Supplementary material for: Phenotypic and Molecular Alterations in the Mammary Tissue of R-Spondin1 Knock-Out Mice during Pregnancy
Source: PLoS One. 2016 Sep 9;11(9):e0162566. doi: 10.1371/journal.pone.0162566 (PMC5017653; doi:10.1371/journal.pone.0162566)
Supplement: S2 Table — (DOCX) [file pone.0162566.s003.docx]

**Supplemental Table 2: Antibody information**

| **Antibodies** | **Reference** | | **Dilution** |
| --- | --- | --- | --- |
| Rabbit polyclonal anti-rat **AQP5** | Alpha diagnostic | AQP51-A | 1/100 |
| Rabbit polyclonal anti-mouse **Npt2b** | Alpha diagnostic | NPT2B11-A | 1/50 |
| Mouse monoclonal anti human **E-Cadherin** | BD Biosciences | 610182 | 1/200 |
| Rabbit polyclonal anti-**Occludin** (C-term) | Invitrogen | 40-4700 | 1/100 |
| Rabbit polyclonal anti-human **ZO1** | Millipore | AB2272 | 1/200 |
| Mouse monoclonal anti **α-SMA** Cy3 conjugated | Sigma | C6198 | 1/200 |
| Rabbit monoclonal anti-human **Ki67** | Thermo Scientific | MA1-39550 | 1/200 |
| Alexafluor 488 goat anti rabbit | Invitrogen | A-11008 | 1/2000 |
| Alexafluor 594 goat anti mouse | Invitrogen | A-11005 | 1/2000 |
