# Supplementary material for: Phenotypic and Molecular Alterations in the Mammary Tissue of R-Spondin1 Knock-Out Mice during Pregnancy
Source: PLoS One. 2016 Sep 9;11(9):e0162566. doi: 10.1371/journal.pone.0162566 (PMC5017653; doi:10.1371/journal.pone.0162566)
Supplement: S3 Table — (DOCX) [file pone.0162566.s004.docx]

**Supplemental Table 3:** List of deregulated genes in *Rspo1*^-/-^ *versus* WT samples at pregnancy day-12

| **Genes** | **Affymetrix Probe Set ID** | **mRNA Accession** | **adjusted p-value** | **Fold Change** |
| --- | --- | --- | --- | --- |
| Gjb6 | 10420366 | NM_001010937 | 6,41E-04 | -31,49 |
| Fabp3 | 10508614 | NM_010174 | 1,28E-02 | -30,39 |
| Chrdl2 | 10555280 | NM_133709 | 2,08E-03 | -26,62 |
| Wap | 10384212 | NM_011709 | 3,87E-02 | -18,37 |
| Rgs8 | 10350725 | NM_026380 | 3,96E-03 | -14,59 |
| Lao1 | 10507580 | NM_133892 | 3,38E-02 | -13,82 |
| Olah | 10479752 | NM_145921 | 6,26E-03 | -13,54 |
| Pigr | 10349580 | NM_011082 | 1,11E-02 | -10,65 |
| Rgs16 | 10350733 | NM_011267 | 3,89E-03 | -9,28 |
| Gjb2 | 10420362 | NM_008125 | 1,14E-03 | -7,89 |
| Gm12863 | 10515839 | ENSMUST00000097910 | 1,78E-02 | -7,59 |
| D730005E14Rik | 10430645 | NR_030675 | 1,14E-03 | -7,19 |
| Atp2c2 | 10575926 | NM_026922 | 1,49E-02 | -6,81 |
| Bhlha15 | 10527323 | NM_010800 | 8,20E-03 | -6,78 |
| Slc9a4 | 10345840 | NM_177084 | 2,35E-02 | -6,7 |
| Ankrd22 | 10467115 | NM_024204 | 1,53E-02 | -6,59 |
| Gldc | 10466976 | NM_138595 | 2,79E-02 | -6,57 |
| Car6 | 10518751 | NM_009802 | 1,36E-02 | -6,44 |
| Duoxa1 | 10486988 | NM_145395 | 2,64E-03 | -5,83 |
| Gm10863 | 10425265 | ENSMUST00000100455 | 5,97E-03 | -5,74 |
| Rspo1 | 10508012 | NM_138683 | 1,14E-03 | -4,93 |
| Spp1 | 10523717 | NM_009263 | 1,18E-02 | -4,91 |
| Gm10384 | 10428209 | ENSMUST00000100713 | 4,95E-03 | -4,78 |
| Mapk4 | 10459747 | NM_172632 | 1,23E-02 | -4,62 |
| Stx19 | 10436451 | NM_026588 | 2,52E-02 | -4,56 |
| Apln | 10604375 | NM_013912 | 2,07E-02 | -4,52 |
| Syt9 | 10556067 | NM_021889 | 4,69E-03 | -4,41 |
| Atp6v1c2 | 10399559 | NM_001159632 | 8,34E-03 | -4,35 |
| Slc16a12 | 10467153 | NM_172838 | 2,52E-02 | -3,96 |
| Duox1 | 10475456 | NM_001099297 | 2,15E-02 | -3,79 |
| Folr1 | 10566034 | NM_008034 | 1,28E-02 | -3,73 |
| Slc5a1 | 10521038 | NM_019810 | 1,53E-02 | -3,66 |
| Muc4 | 10435112 | NM_080457 | 1,14E-02 | -3,61 |
| Scrg1 | 10571865 | NM_009136 | 1,67E-03 | -3,61 |
| Lrrc7 | 10503054 | NM_001081358 | 4,94E-02 | -3,58 |
| Fam20c | 10526853 | NM_030565 | 1,80E-02 | -3,55 |
| Apobec3 | 10425333 | ENSMUST00000100423 | 1,93E-03 | -3,5 |
| Gzmb | 10420308 | NM_013542 | 1,66E-02 | -3,39 |
| Rnase1 | 10419563 | NM_011271 | 1,28E-02 | -3,38 |
| Gdap1l1 | 10478374 | NM_144891 | 3,39E-02 | -3,36 |
| Bcl2l15 | 10494972 | NM_001142959 | 1,49E-02 | -3,34 |
| Tmprss4 | 10593035 | NM_145403 | 4,83E-02 | -3,32 |
| Fam46c | 10500610 | NM_001142952 | 3,08E-02 | -3,26 |
| 2200002K05Rik | 10583291 | BC055786 | 1,53E-02 | -3,13 |
| Kcnk6 | 10561702 | NM_001033525 | 3,51E-02 | -3,05 |
| Card14 | 10383109 | NM_130886 | 2,08E-02 | -3,03 |
| Ccdc64b | 10442311 | NM_153784 | 2,57E-02 | -2,99 |
| Baiap2l1 | 10535559 | NM_025833 | 1,71E-02 | -2,92 |
| D730002M21Rik | 10464110 | ENSMUST00000099314 | 2,60E-02 | -2,9 |
| Sidt1 | 10439583 | NM_001159419 | 1,11E-02 | -2,9 |
| Ttc9 | 10396952 | NM_001033149 | 2,61E-03 | -2,88 |
| Hhipl2 | 10598126 | NM_030175 | 3,23E-02 | -2,87 |
| Fam20a | 10392464 | NM_153782 | 1,80E-02 | -2,86 |
| Rogdi | 10437483 | NM_133185 | 1,22E-02 | -2,84 |
| Kctd14 | 10555059 | NM_001012434 | 2,84E-02 | -2,82 |
| Bspry | 10505299 | NM_138653 | 1,58E-02 | -2,8 |
| Tgfb2 | 10360920 | NM_009367 | 1,22E-02 | -2,79 |
| Cited4 | 10507726 | NM_019563 | 1,45E-02 | -2,78 |
| 9130230L23Rik | 10530259 | NR_027961 | 7,51E-03 | -2,76 |
| Cytip | 10482802 | NM_139200 | 1,20E-02 | -2,76 |
| G630090E17Rik | 10368700 | NM_001173500 | 2,36E-02 | -2,75 |
| Ankrd56 | 10531484 | NM_175270 | 3,54E-02 | -2,74 |
| Fkbp11 | 10432243 | NM_024169 | 3,84E-02 | -2,74 |
| Scgb1c1 | 10558694 | NM_001099742 | 1,53E-02 | -2,74 |
| Tnfrsf19 | 10420596 | NM_013869 | 6,41E-04 | -2,7 |
| Acsl4 | 10607089 | NM_207625 | 1,65E-02 | -2,68 |
| Plcxd2 | 10439732 | NM_001134480 | 1,22E-02 | -2,65 |
| Meig1 | 10479761 | NM_008579 | 4,60E-03 | -2,64 |
| Il18r1 | 10345807 | NM_008365 | 3,08E-02 | -2,62 |
| Npnt | 10502240 | NM_033525 | 2,52E-02 | -2,62 |
| Ovol1 | 10465106 | NM_019935 | 3,89E-03 | -2,61 |
| Pik3c2g | 10542477 | NM_207683 | 1,21E-02 | -2,58 |
| Car2 | 10490923 | NM_009801 | 1,28E-02 | -2,57 |
| Slc30a2 | 10508961 | NM_001039677 | 4,40E-02 | -2,57 |
| St6gal1 | 10434758 | NM_145933 | 2,64E-02 | -2,57 |
| Tfrc | 10435075 | NM_011638 | 2,21E-02 | -2,54 |
| Gpr110 | 10445251 | NM_133776 | 2,52E-02 | -2,53 |
| Bcl2l14 | 10542287 | NM_025778 | 5,97E-03 | -2,49 |
| Kcnn4 | 10550877 | NM_008433 | 1,71E-02 | -2,48 |
| Csn1s2b | 10522858 | NM_009973 | 4,80E-02 | -2,47 |
| Slc6a14 | 10599008 | NM_020049 | 1,22E-02 | -2,46 |
| Atp7b | 10577449 | NM_007511 | 4,11E-02 | -2,45 |
| Rnf207 | 10518909 | NM_001033489 | 2,74E-02 | -2,44 |
| Apol7a | 10430166 | NM_029419 | 2,76E-02 | -2,42 |
| Tc2n | 10402195 | NM_028924 | 3,23E-02 | -2,42 |
| Fam189a2 | 10466735 | NM_001114174 | 8,20E-03 | -2,41 |
| Trim7 | 10375503 | NM_053166 | 2,18E-02 | -2,41 |
| 2010011I20Rik | 10478962 | NM_025912 | 1,18E-02 | -2,4 |
| BC005685 | 10401935 | BC005685 | 2,74E-02 | -2,39 |
| Slc38a3 | 10596718 | NM_023805 | 3,80E-02 | -2,39 |
| 1700040L02Rik | 10369806 | BC087900 | 8,20E-03 | -2,35 |
| Muc20 | 10439087 | NM_146071 | 3,23E-02 | -2,35 |
| Upb1 | 10364038 | NM_133995 | 1,43E-03 | -2,35 |
| Got1 | 10467842 | NM_010324 | 5,97E-03 | -2,34 |
| Sult1d1 | 10531100 | NM_016771 | 1,41E-02 | -2,34 |
| Gpr172b | 10424922 | NM_029643 | 3,10E-02 | -2,33 |
| 2810459M11Rik | 10348000 | NM_001144992 | 1,53E-02 | -2,32 |
| Calml3 | 10407416 | NM_027416 | 2,18E-02 | -2,31 |
| 1700012B07Rik | 10392476 | NM_027038 | 1,28E-02 | -2,3 |
| Cbx7 | 10430649 | NM_144811 | 1,43E-03 | -2,29 |
| Pglyrp1 | 10550509 | NM_009402 | 3,39E-02 | -2,28 |
| Gm12474 | 10494757 | ENSMUST00000066727 | 5,00E-02 | -2,26 |
| Grhl1 | 10394954 | NM_001161406 | 2,77E-02 | -2,25 |
| Ica1 | 10543120 | NM_010492 | 3,90E-02 | -2,25 |
| Timd2 | 10385455 | NM_001161355 | 4,63E-02 | -2,25 |
| Cldn8 | 10440647 | NM_018778 | 2,53E-02 | -2,24 |
| Cdhr4 | 10588819 | NM_001122635 | 2,92E-02 | -2,23 |
| Ehhadh | 10438575 | NM_023737 | 2,24E-02 | -2,21 |
| Liph | 10438592 | NM_001083894 | 7,97E-03 | -2,21 |
| Hlf | 10389786 | NM_172563 | 2,79E-02 | -2,19 |
| Fn3k | 10383564 | NM_001038699 | 7,17E-03 | -2,18 |
| Naaladl2 | 10497501 | XM_910834 | 4,31E-02 | -2,18 |
| Susd4 | 10352439 | NM_144796 | 3,96E-02 | -2,17 |
| Cd7 | 10394054 | NM_009854 | 4,08E-02 | -2,16 |
| Spnb1 | 10401068 | NM_013675 | 2,67E-02 | -2,15 |
| Scd3 | 10463347 | NM_024450 | 8,20E-03 | -2,12 |
| Elovl7 | 10407072 | NM_029001 | 3,39E-02 | -2,11 |
| Ptpn22 | 10494978 | NM_008979 | 2,76E-02 | -2,11 |
| Acbd7 | 10468945 | NM_030063 | 8,20E-03 | -2,1 |
| Crabp2 | 10493108 | NM_007759 | 3,19E-02 | -2,1 |
| Igsf5 | 10437195 | NM_001177887 | 4,59E-02 | -2,1 |
| Myo5c | 10587150 | NM_001081322 | 3,46E-02 | -2,1 |
| Gm10044 | 10417517 | ENSMUST00000081331 | 3,35E-02 | -2,08 |
| Tnik | 10491136 | NM_026910 | 1,13E-02 | -2,08 |
| Cd82 | 10485213 | NM_007656 | 1,80E-02 | -2,06 |
| Arhgdig | 10449258 | NM_008113 | 1,37E-02 | -2,05 |
| 4930451G09Rik | 10433340 | ENSMUST00000090457 | 1,80E-02 | -2,04 |
| Sh3yl1 | 10395142 | NM_013709 | 2,08E-02 | -2,03 |
| Tmprss13 | 10584870 | NM_001013373 | 4,30E-02 | -2,03 |
| Ly6g6d | 10450418 | NM_033478 | 1,12E-02 | -2,02 |
| Mast4 | 10411804 | NM_175171 | 1,18E-02 | -2,02 |
| Rab4a | 10576391 | NM_009003 | 1,05E-02 | -2,02 |
| 9930023K05Rik | 10464137 | NM_172641 | 6,09E-03 | -2,01 |
| Aass | 10543333 | NM_013930 | 3,99E-02 | -2,01 |
| Elf5 | 10474171 | NM_010125 | 4,84E-02 | -2,01 |
| Fam108c | 10565288 | NM_133722 | 4,28E-02 | -2,01 |
| Sema4d | 10409240 | NM_013660 | 1,52E-02 | -2,01 |
| Nucb2 | 10556583 | NM_001130479 | 2,79E-02 | -2 |
| Stat5a | 10381172 | NM_011488 | 3,47E-02 | -2 |
| Csmd1 | 10577240 | NM_053171 | 4,52E-02 | 2 |
| Hmcn1 | 10358664 | NM_001024720 | 3,10E-02 | 2 |
| Anxa8 | 10414065 | NM_013473 | 1,89E-02 | 2,02 |
| Dsc3 | 10457669 | NM_007882 | 1,73E-02 | 2,02 |
| Pkd1l1 | 10384266 | ENSMUST00000055297 | 3,88E-02 | 2,02 |
| Adamts15 | 10591988 | NM_001024139 | 8,62E-03 | 2,03 |
| S100a6 | 10493820 | NM_011313 | 1,89E-02 | 2,03 |
| Igsf9 | 10351801 | NM_033608 | 1,49E-02 | 2,04 |
| Mdga2 | 10400607 | AK163637 | 7,97E-03 | 2,04 |
| Elovl4 | 10595392 | NM_148941 | 3,01E-02 | 2,05 |
| Gja1 | 10363173 | NM_010288 | 4,12E-02 | 2,05 |
| Adamts14 | 10369431 | NM_001081127 | 4,98E-02 | 2,06 |
| Basp1 | 10427895 | NM_027395 | 2,26E-03 | 2,06 |
| Cdk14 | 10527940 | NM_011074 | 3,35E-02 | 2,06 |
| Col3a1 | 10346015 | NM_009930 | 2,61E-02 | 2,06 |
| Slc44a4 | 10444554 | NM_023557 | 5,97E-03 | 2,06 |
| 9530053A07Rik | 10551435 | NM_001164655 | 8,69E-03 | 2,07 |
| Bcat1 | 10549222 | NM_001024468 | 4,06E-02 | 2,07 |
| Inhbb | 10357155 | NM_008381 | 1,41E-02 | 2,07 |
| Plod2 | 10587829 | NM_001142916 | 1,28E-02 | 2,07 |
| Arhgap40 | 10478114 | NM_001145015 | 2,08E-03 | 2,08 |
| Slc35f3 | 10576586 | NM_175434 | 3,19E-02 | 2,08 |
| Ccdc141 | 10484205 | ENSMUST00000049544 | 1,45E-02 | 2,09 |
| Col1a1 | 10380419 | NM_007742 | 1,36E-02 | 2,09 |
| Gadd45b | 10364950 | NM_008655 | 4,45E-02 | 2,1 |
| Col1a2 | 10536220 | NM_007743 | 1,49E-02 | 2,11 |
| Stra6 | 10585803 | NM_009291 | 5,97E-03 | 2,14 |
| 2810055G20Rik | 10436598 | ENSMUST00000068704 | 1,80E-02 | 2,16 |
| Bmp3 | 10523506 | NM_173404 | 1,52E-02 | 2,16 |
| Capn8 | 10352416 | NM_130890 | 1,50E-02 | 2,16 |
| Ephb3 | 10434559 | NM_010143 | 8,47E-03 | 2,16 |
| Rgs2 | 10358389 | NM_009061 | 1,28E-02 | 2,16 |
| D8Ertd82e | 10571344 | NM_172911 | 6,09E-03 | 2,17 |
| Hpgd | 10571840 | NM_008278 | 5,00E-02 | 2,17 |
| A930038C07Rik | 10538802 | NM_172399 | 2,84E-02 | 2,18 |
| Col5a2 | 10354309 | NM_007737 | 4,16E-02 | 2,18 |
| Ctnnd2 | 10423471 | NM_008729 | 1,58E-02 | 2,21 |
| Gm9930 | 10367770 | ENSMUST00000066742 | 1,33E-02 | 2,21 |
| Krt7 | 10427052 | NM_033073 | 7,17E-03 | 2,21 |
| Pappa | 10505489 | NM_021362 | 8,20E-03 | 2,22 |
| Trpv6 | 10544348 | NM_022413 | 1,14E-03 | 2,22 |
| Nkd2 | 10410547 | NM_028186 | 3,89E-03 | 2,23 |
| Cldn10a | 10417027 | NM_021386 | 2,76E-02 | 2,24 |
| Gm10484 | 10527963 | AK142929 | 1,22E-02 | 2,24 |
| Ucma | 10469058 | NM_001113558 | 5,97E-03 | 2,25 |
| Trpm3 | 10462039 | NM_001035244 | 1,49E-02 | 2,26 |
| Bace2 | 10437210 | NM_019517 | 1,37E-03 | 2,28 |
| Adam12 | 10568668 | NM_007400 | 2,48E-02 | 2,33 |
| Ntng1 | 10501468 | NM_030699 | 5,97E-03 | 2,33 |
| Hey2 | 10368556 | NM_013904 | 3,83E-02 | 2,35 |
| Slc13a2 | 10388834 | NM_022411 | 1,50E-02 | 2,35 |
| Cacna1g | 10389929 | NM_009783 | 2,78E-02 | 2,37 |
| Alox12e | 10387838 | NM_145684 | 2,87E-02 | 2,39 |
| Hey1 | 10497203 | NM_010423 | 1,78E-02 | 2,39 |
| Moxd1 | 10362186 | NM_021509 | 8,69E-03 | 2,39 |
| Areg | 10523182 | NM_009704 | 1,22E-02 | 2,41 |
| Etv4 | 10391490 | NM_008815 | 3,89E-03 | 2,43 |
| Slc6a15 | 10366163 | NM_175328 | 2,64E-03 | 2,43 |
| Aldh1a3 | 10564417 | NM_053080 | 3,47E-02 | 2,47 |
| AF529169 | 10595657 | AF529169 | 1,79E-03 | 2,48 |
| Emb | 10407327 | NM_010330 | 1,58E-02 | 2,51 |
| Pof1b | 10606495 | NM_181579 | 2,76E-02 | 2,52 |
| Mtmr7 | 10578300 | NM_001040699 | 2,00E-03 | 2,54 |
| Foxi1 | 10385114 | NM_023907 | 1,88E-03 | 2,55 |
| Myb | 10368199 | NM_010848 | 1,14E-02 | 2,55 |
| Sv2b | 10564646 | NM_001109753 | 3,08E-02 | 2,55 |
| Cdh2 | 10457644 | NM_007664 | 1,02E-02 | 2,59 |
| Crispld1 | 10344990 | NM_031402 | 3,58E-02 | 2,59 |
| Dsg1a | 10454113 | NM_010079 | 3,10E-02 | 2,59 |
| Slc5a8 | 10365640 | NM_145423 | 3,39E-02 | 2,59 |
| Ceacam20 | 10550740 | NM_027839 | 8,20E-03 | 2,6 |
| Dsp | 10404649 | NM_023842 | 1,39E-02 | 2,62 |
| Ugt8a | 10501963 | NM_011674 | 1,03E-03 | 2,65 |
| Rasgef1c | 10375650 | NM_029004 | 2,15E-02 | 2,7 |
| Itih2 | 10480003 | NM_010582 | 6,95E-03 | 2,71 |
| Gm9911 | 10578017 | AK035883 | 3,33E-02 | 2,72 |
| Capn6 | 10607143 | NM_007603 | 3,54E-02 | 2,78 |
| Bhlhe41 | 10549276 | NM_024469 | 1,43E-03 | 2,81 |
| Hp | 10581605 | NM_017370 | 1,58E-02 | 2,83 |
| Tnfaip2 | 10398665 | NM_009396 | 1,25E-03 | 2,87 |
| Gm266 | 10402705 | NM_001033248 | 2,20E-02 | 2,95 |
| R3hdml | 10478383 | NM_001099331 | 1,96E-02 | 2,98 |
| Wfdc2 | 10478525 | NM_026323 | 5,31E-03 | 3 |
| Vcan | 10410931 | NM_001081249 | 1,25E-02 | 3,01 |
| 4921506M07Rik | 10395869 | BC100489 | 8,24E-03 | 3,04 |
| Slit2 | 10521759 | NM_178804 | 1,28E-02 | 3,08 |
| Gipc2 | 10502816 | NM_016867 | 2,57E-02 | 3,17 |
| Gna14 | 10461856 | NM_008137 | 1,79E-03 | 3,18 |
| Egr2 | 10363735 | NM_010118 | 3,40E-02 | 3,22 |
| Samd5 | 10367772 | NM_177271 | 8,69E-03 | 3,27 |
| Cited1 | 10606083 | NM_007709 | 1,53E-02 | 3,28 |
| Esm1 | 10407281 | NM_023612 | 5,59E-03 | 3,34 |
| Ptn | 10543959 | NM_008973 | 1,52E-03 | 3,39 |
| Ccdc129 | 10538503 | NM_001081665 | 1,45E-02 | 3,44 |
| Foxa1 | 10400504 | NM_008259 | 5,97E-03 | 3,46 |
| Crabp1 | 10585438 | NM_013496 | 1,18E-02 | 3,77 |
| Spib | 10562812 | NM_019866 | 1,28E-02 | 4,08 |
| Fut9 | 10511870 | NM_010243 | 3,69E-04 | 4,16 |
| Clic6 | 10436958 | NM_172469 | 1,88E-02 | 4,2 |
| Them5 | 10494016 | NM_025416 | 2,74E-02 | 4,2 |
| St8sia6 | 10480238 | NM_145838 | 1,14E-03 | 4,53 |
| Slco1a5 | 10549041 | NM_130861 | 1,78E-04 | 6,26 |
| Msln | 10449000 | NM_018857 | 3,02E-03 | 6,54 |
| Cybrd1 | 10472757 | NM_028593 | 5,27E-05 | 7,1 |
| Fbn2 | 10458999 | NM_010181 | 1,43E-03 | 7,37 |
| Lect1 | 10421853 | NM_010701 | 2,51E-03 | 8,65 |
| C530030P08Rik | 10375123 | ENSMUST00000101381 | 2,87E-05 | 23,23 |
|  |  |  |  |  |
