# Supplementary material for: Phenotypic and Molecular Alterations in the Mammary Tissue of R-Spondin1 Knock-Out Mice during Pregnancy
Source: PLoS One. 2016 Sep 9;11(9):e0162566. doi: 10.1371/journal.pone.0162566 (PMC5017653; doi:10.1371/journal.pone.0162566)
Supplement: S4 Table — (DOCX) [file pone.0162566.s005.docx]

**Supplemental Table 4:** List of deregulated genes in *Rspo1*^-/-^ *versus* WT samples at pregnancy day-16

| **Genes** | **Affymetrix Probe Set ID** | **mRNA Accession** | **Adjusted p-value** | **Fold Change** |
| --- | --- | --- | --- | --- |
| Gjb6 | 10420366 | NM_001010937 | 3,65E-04 | -44,83 |
| Rgs8 | 10350725 | NM_026380 | 3,94E-05 | -37,22 |
| Slc9a4 | 10345840 | NM_177084 | 8,30E-04 | -28,57 |
| Chrdl2 | 10555280 | NM_133709 | 6,96E-04 | -26,22 |
| Drd4 | 10558866 | NM_007878 | 7,18E-05 | -22,17 |
| Gldc | 10466976 | NM_138595 | 3,49E-04 | -20,87 |
| Csn1s2b | 10522858 | NM_009973 | 1,03E-04 | -18,37 |
| Sftpd | 10419096 | NM_009160 | 4,85E-04 | -18,02 |
| Mapk4 | 10459747 | NM_172632 | 3,94E-05 | -17,98 |
| Gm12863 | 10515839 | ENSMUST00000097910 | 1,53E-03 | -17,87 |
| Rgs16 | 10350733 | NM_011267 | 1,67E-04 | -17,84 |
| Atp2c2 | 10575926 | NM_026922 | 2,77E-04 | -16,48 |
| Pigr | 10349580 | NM_011082 | 2,85E-04 | -15,25 |
| Apln | 10604375 | NM_013912 | 2,77E-04 | -15,04 |
| Olah | 10479752 | NM_145921 | 1,22E-03 | -14,4 |
| Ankrd22 | 10467115 | NM_024204 | 2,77E-04 | -14,27 |
| Saa2 | 10553274 | NM_011314 | 5,97E-03 | -14,18 |
| D730005E14Rik | 10430645 | NR_030675 | 2,11E-04 | -12,87 |
| Stx19 | 10436451 | NM_026588 | 2,63E-04 | -11,67 |
| Tmprss4 | 10593035 | NM_145403 | 1,03E-04 | -11,06 |
| Fabp3 | 10508614 | NM_010174 | 1,23E-02 | -11,02 |
| Gjb2 | 10420362 | NM_008125 | 2,77E-04 | -9,34 |
| 1810065E05Rik | 10376376 | NM_027239 | 1,76E-03 | -9,01 |
| Dkkl1 | 10563170 | NM_015789 | 5,51E-04 | -8,99 |
| Slc30a2 | 10508961 | NM_001039677 | 4,03E-05 | -8,96 |
| Syt9 | 10556067 | NM_021889 | 6,11E-05 | -8,94 |
| Gm10863 | 10425265 | ENSMUST00000100455 | 8,48E-04 | -8,7 |
| Calml3 | 10407416 | NM_027416 | 3,49E-04 | -8,47 |
| Atp2b2 | 10546977 | NM_009723 | 4,03E-05 | -8,14 |
| Abcg2 | 10538640 | NM_011920 | 1,03E-04 | -8,12 |
| Duox1 | 10475456 | NM_001099297 | 6,63E-04 | -7,95 |
| Slc5a1 | 10521038 | NM_019810 | 3,94E-05 | -7,83 |
| Trim55 | 10490972 | NM_001081281 | 1,93E-03 | -7,76 |
| Cel | 10481278 | NM_009885 | 1,36E-03 | -7,64 |
| Ankrd56 | 10531484 | NM_175270 | 8,23E-05 | -7,33 |
| Bhlha15 | 10527323 | NM_010800 | 1,74E-03 | -7,26 |
| Aqp3 | 10512156 | NM_016689 | 1,07E-03 | -7,21 |
| Aass | 10543333 | NM_013930 | 5,88E-04 | -7,2 |
| Gsdmc | 10428943 | NM_031378 | 1,26E-04 | -6,98 |
| Duoxa1 | 10486988 | NM_145395 | 4,86E-04 | -6,79 |
| Fam20c | 10526853 | NM_030565 | 1,62E-04 | -6,66 |
| Fam46c | 10500610 | NM_001142952 | 1,81E-04 | -6,61 |
| Ccdc74a | 10434089 | NM_001166164 | 1,26E-04 | -6,54 |
| D730002M21Rik | 10464110 | ENSMUST00000099314 | 4,07E-04 | -6,51 |
| Kcnk6 | 10561702 | NM_001033525 | 1,26E-04 | -6,34 |
| Wap | 10384212 | NM_011709 | 3,36E-02 | -6,33 |
| Slc16a12 | 10467153 | NM_172838 | 3,28E-04 | -6,28 |
| Cytip | 10482802 | NM_139200 | 1,67E-04 | -6,26 |
| Wfdc3 | 10489535 | NM_027961 | 3,50E-03 | -6,16 |
| Gnmt | 10451451 | NM_010321 | 6,30E-04 | -6,14 |
| Lpo | 10389639 | NM_080420 | 3,36E-03 | -6,11 |
| Lao1 | 10507580 | NM_133892 | 7,92E-03 | -5,99 |
| Fkbp11 | 10432243 | NM_024169 | 3,02E-04 | -5,88 |
| Arg2 | 10396831 | NM_009705 | 4,07E-04 | -5,77 |
| Hephl1 | 10590992 | NM_001164797 | 5,44E-04 | -5,77 |
| Card14 | 10383109 | NM_130886 | 1,26E-04 | -5,73 |
| Cd320 | 10444046 | NM_019421 | 1,30E-04 | -5,67 |
| Lalba | 10432157 | NM_010679 | 9,99E-03 | -5,63 |
| Cidea | 10456392 | NM_007702 | 1,36E-03 | -5,61 |
| Rab15 | 10401114 | NM_134050 | 1,56E-04 | -5,57 |
| Folr1 | 10566034 | NM_008034 | 3,49E-04 | -5,53 |
| 2200002K05Rik | 10583291 | BC055786 | 6,18E-04 | -5,5 |
| Il18r1 | 10345807 | NM_008365 | 1,42E-03 | -5,47 |
| Scd3 | 10463347 | NM_024450 | 6,83E-04 | -5,33 |
| St6gal1 | 10434758 | NM_145933 | 2,25E-04 | -5,22 |
| Sgk2 | 10478326 | NM_013731 | 9,93E-05 | -5,21 |
| G630090E17Rik | 10368700 | NM_001173500 | 2,11E-04 | -5,15 |
| Tfrc | 10435075 | NM_011638 | 3,49E-04 | -5,08 |
| D10Bwg1379e | 10368101 | NM_001033258 | 5,19E-04 | -5,04 |
| Cnga4 | 10555925 | NM_001033317 | 1,17E-03 | -5,03 |
| Ucp3 | 10555378 | NM_009464 | 3,33E-03 | -4,99 |
| Tnfrsf19 | 10420596 | NM_013869 | 1,16E-04 | -4,94 |
| Lgr4 | 10474419 | NM_172671 | 8,23E-05 | -4,92 |
| Sectm1b | 10394060 | NM_026907 | 1,11E-03 | -4,81 |
| Plcxd2 | 10439732 | NM_001134480 | 1,27E-03 | -4,64 |
| Gdap1l1 | 10478374 | NM_144891 | 9,79E-04 | -4,62 |
| B4galt1 | 10512129 | NM_022305 | 2,11E-04 | -4,59 |
| Ccdc64b | 10442311 | NM_153784 | 2,25E-04 | -4,55 |
| Lrp8 | 10506714 | NR_033496 | 7,27E-04 | -4,53 |
| Muc4 | 10435112 | NM_080457 | 3,49E-04 | -4,53 |
| Slc34a2 | 10521892 | NM_011402 | 2,41E-04 | -4,5 |
| Atp6v1c2 | 10399559 | NM_001159632 | 1,57E-04 | -4,49 |
| Rnase1 | 10419563 | NM_011271 | 3,13E-03 | -4,49 |
| Dhdpsl | 10463200 | NM_026152 | 9,18E-04 | -4,46 |
| Slc26a7 | 10511631 | NM_145947 | 9,17E-03 | -4,46 |
| Il15 | 10579958 | NM_008357 | 2,63E-04 | -4,42 |
| Liph | 10438592 | NM_001083894 | 4,07E-04 | -4,42 |
| Slc18a1 | 10579023 | NM_153054 | 9,04E-04 | -4,4 |
| Gm10384 | 10428209 | ENSMUST00000100713 | 8,65E-04 | -4,38 |
| Apol7a | 10430166 | NM_029419 | 1,11E-03 | -4,32 |
| Susd4 | 10352439 | NM_144796 | 1,56E-04 | -4,3 |
| Cmtm6 | 10589929 | NM_026036 | 1,24E-04 | -4,29 |
| Gm11437 | 10389261 | NM_001037932 | 2,77E-04 | -4,29 |
| Atp13a4 | 10438854 | NM_001164612 | 4,20E-03 | -4,28 |
| Tmem125 | 10515797 | NM_172383 | 3,91E-04 | -4,27 |
| Doc2b | 10388465 | NM_007873 | 3,02E-04 | -4,26 |
| Acsl4 | 10607089 | NM_207625 | 3,24E-03 | -4,25 |
| Btg3 | 10445875 | NM_009770 | 1,56E-04 | -4,23 |
| Tmem144 | 10498871 | NM_027495 | 1,47E-03 | -4,18 |
| Mesdc1 | 10565250 | NM_030705 | 3,58E-04 | -4,17 |
| Gyk | 10605571 | NM_212444 | 4,49E-04 | -4,1 |
| Prox1 | 10361023 | NM_008937 | 2,17E-03 | -4,08 |
| Scgb1c1 | 10558694 | NM_001099742 | 5,75E-04 | -4,07 |
| Il18rap | 10345824 | NM_010553 | 8,57E-04 | -4,04 |
| Slc6a14 | 10599008 | NM_020049 | 1,17E-03 | -4,03 |
| Tgm1 | 10420114 | NM_001161715 | 5,61E-04 | -4 |
| Meig1 | 10479761 | NM_008579 | 1,07E-03 | -3,98 |
| Ehhadh | 10438575 | NM_023737 | 1,24E-04 | -3,94 |
| Gpr172b | 10424922 | NM_029643 | 4,69E-04 | -3,93 |
| Slc16a1 | 10495035 | NM_009196 | 2,25E-04 | -3,91 |
| Ass1 | 10363541 | NM_007494 | 3,49E-04 | -3,89 |
| Saa3 | 10563597 | NM_011315 | 1,03E-02 | -3,83 |
| Got1 | 10467842 | NM_010324 | 1,67E-04 | -3,82 |
| Ovol1 | 10465106 | NM_019935 | 2,62E-03 | -3,82 |
| Acot6 | 10397172 | NM_172580 | 1,03E-04 | -3,78 |
| Atp7b | 10577449 | NM_007511 | 7,82E-04 | -3,77 |
| Baiap2l1 | 10535559 | NM_025833 | 1,89E-04 | -3,77 |
| BC022713 | 10538273 | BC022713 | 1,17E-03 | -3,77 |
| Ccl28 | 10598203 | NM_020279 | 1,09E-03 | -3,76 |
| Hhipl2 | 10598126 | NM_030175 | 3,49E-04 | -3,75 |
| Lrrc10 | 10366541 | NM_146242 | 2,77E-04 | -3,74 |
| 2310014L17Rik | 10550059 | NM_029809 | 3,09E-04 | -3,73 |
| Hpse2 | 10467826 | NM_001081257 | 3,24E-03 | -3,72 |
| Slc9a2 | 10345855 | NM_001033289 | 6,83E-04 | -3,71 |
| Ttc39a | 10506968 | NM_153392 | 3,09E-04 | -3,69 |
| Tmem165 | 10522596 | NM_011626 | 2,19E-04 | -3,68 |
| Car6 | 10518751 | NM_009802 | 1,79E-03 | -3,65 |
| Ttc9 | 10396952 | NM_001033149 | 8,80E-04 | -3,64 |
| Tc2n | 10402195 | NM_028924 | 1,53E-02 | -3,62 |
| Apob48r | 10557434 | NM_138310 | 1,79E-03 | -3,61 |
| Upb1 | 10364038 | NM_133995 | 1,13E-03 | -3,61 |
| Bspry | 10505299 | NM_138653 | 2,37E-04 | -3,59 |
| Gm1973 | 10417245 | NM_029288 | 2,18E-03 | -3,59 |
| Muc1 | 10493474 | NM_013605 | 3,49E-04 | -3,59 |
| Spp1 | 10523717 | NM_009263 | 2,04E-03 | -3,59 |
| Mfsd2a | 10516064 | NM_029662 | 4,79E-03 | -3,57 |
| Odc1 | 10394770 | NM_013614 | 3,49E-04 | -3,57 |
| Gzmb | 10420308 | NM_013542 | 2,04E-03 | -3,54 |
| Fam20a | 10392464 | NM_153782 | 3,74E-04 | -3,53 |
| Gm12474 | 10494757 | ENSMUST00000066727 | 1,79E-03 | -3,53 |
| D830030K20Rik | 10412549 | NM_177135 | 2,62E-03 | -3,52 |
| Fam160a2 | 10566438 | NM_199009 | 4,53E-04 | -3,49 |
| Gadd45g | 10405211 | NM_011817 | 2,84E-03 | -3,43 |
| Nucb2 | 10556583 | NM_001130479 | 1,25E-03 | -3,4 |
| Sec11c | 10456346 | NM_025468 | 1,34E-03 | -3,39 |
| 2010011I20Rik | 10478962 | NM_025912 | 2,23E-04 | -3,36 |
| Samd12 | 10428594 | NM_177225 | 5,63E-03 | -3,32 |
| Slc28a3 | 10409713 | NM_022317 | 2,19E-04 | -3,3 |
| Ly6g6d | 10450418 | NM_033478 | 3,25E-04 | -3,28 |
| 2210407C18Rik | 10386197 | NM_144544 | 1,39E-02 | -3,27 |
| F5 | 10351224 | NM_007976 | 5,19E-04 | -3,27 |
| Slc37a1 | 10443764 | NM_153062 | 3,50E-04 | -3,25 |
| Npnt | 10502240 | NM_033525 | 9,04E-04 | -3,23 |
| Olfm4 | 10416689 | NM_001030294 | 1,81E-03 | -3,22 |
| Slc25a34 | 10517988 | NM_001013780 | 2,76E-02 | -3,22 |
| Arhgdig | 10449258 | NM_008113 | 8,10E-03 | -3,21 |
| Creb3l1 | 10485117 | NM_011957 | 1,96E-03 | -3,21 |
| Fam189a2 | 10466735 | NM_001114174 | 1,45E-03 | -3,21 |
| Hlf | 10389786 | NM_172563 | 2,11E-04 | -3,21 |
| Elovl7 | 10407072 | NM_029001 | 3,66E-04 | -3,2 |
| Nipal2 | 10428089 | NM_145469 | 2,77E-04 | -3,2 |
| Fut4 | 10590957 | NM_010242 | 3,78E-03 | -3,19 |
| Ammecr1 | 10607116 | NM_019496 | 2,52E-03 | -3,18 |
| BC016579 | 10439667 | NM_145389 | 1,93E-02 | -3,16 |
| Saa1 | 10563611 | NM_009117 | 4,53E-02 | -3,13 |
| Igfals | 10442625 | NM_008340 | 1,33E-03 | -3,12 |
| Rhpn2 | 10552156 | NM_027897 | 1,50E-04 | -3,12 |
| Stx3 | 10466248 | NM_001025307 | 1,17E-03 | -3,1 |
| Slc7a5 | 10582275 | NM_011404 | 1,81E-03 | -3,09 |
| Timd2 | 10385455 | NM_001161355 | 1,57E-03 | -3,09 |
| Ppil6 | 10362794 | NM_028430 | 4,87E-02 | -3,08 |
| Gzma | 10412211 | NM_010370 | 4,06E-02 | -3,06 |
| Slc7a4 | 10438189 | NM_144852 | 5,47E-04 | -3,06 |
| Tcfcp2l1 | 10349295 | NM_023755 | 4,49E-04 | -3,05 |
| G0s2 | 10361246 | NM_008059 | 8,13E-04 | -3,04 |
| Lrrc7 | 10503054 | NM_001081358 | 1,02E-02 | -3,04 |
| Rab18 | 10453705 | NM_181070 | 7,27E-04 | -3,03 |
| Rprm | 10482766 | NM_023396 | 3,35E-02 | -3,03 |
| Sytl1 | 10517009 | NM_031393 | 3,42E-04 | -3,02 |
| 9130230L23Rik | 10530259 | NR_027961 | 3,67E-03 | -3,01 |
| Pglyrp1 | 10550509 | NM_009402 | 1,15E-03 | -3,01 |
| Qsox1 | 10359034 | NM_001024945 | 4,07E-04 | -2,99 |
| Kcnn4 | 10550877 | NM_008433 | 8,48E-04 | -2,97 |
| Pycr1 | 10393887 | NM_144795 | 8,51E-04 | -2,97 |
| Btn2a2 | 10408185 | NM_175938 | 8,51E-04 | -2,96 |
| Vps37b | 10533729 | NM_177876 | 1,18E-03 | -2,96 |
| Gmppb | 10588836 | NM_177910 | 2,32E-03 | -2,95 |
| Pdgfd | 10583021 | NM_027924 | 3,16E-03 | -2,95 |
| Fn3k | 10383564 | NM_001038699 | 2,11E-04 | -2,94 |
| Rapgef5 | 10399148 | NM_175930 | 2,50E-03 | -2,94 |
| Rogdi | 10437483 | NM_133185 | 3,25E-04 | -2,9 |
| Spnb1 | 10401068 | NM_013675 | 3,28E-04 | -2,9 |
| Sult1d1 | 10531100 | NM_016771 | 1,50E-02 | -2,9 |
| Tmprss13 | 10584870 | NM_001013373 | 1,36E-03 | -2,9 |
| 2610528J11Rik | 10507551 | BC022764 | 1,63E-03 | -2,89 |
| BC016548 | 10485461 | BC016548 | 1,79E-03 | -2,89 |
| Fam59a | 10457780 | NM_001033445 | 1,76E-03 | -2,89 |
| Ano4 | 10371740 | NM_178773 | 1,51E-03 | -2,88 |
| B3gnt3 | 10579602 | NM_028189 | 8,57E-04 | -2,88 |
| Ica1 | 10543120 | NM_010492 | 5,44E-04 | -2,86 |
| Acot1 | 10397148 | NM_012006 | 1,99E-03 | -2,85 |
| Mfhas1 | 10571325 | NM_001081279 | 4,07E-04 | -2,84 |
| 9130404H23Rik | 10452566 | NM_028998 | 1,18E-02 | -2,82 |
| Clcn3 | 10578810 | NM_173874 | 4,90E-04 | -2,8 |
| Kctd14 | 10555059 | NM_001012434 | 7,83E-04 | -2,8 |
| Slc30a4 | 10487021 | NM_011774 | 1,73E-03 | -2,8 |
| Mfsd7b | 10361065 | NM_001081259 | 1,62E-03 | -2,78 |
| Scrg1 | 10571865 | NM_009136 | 4,40E-03 | -2,78 |
| Mfap3l | 10571907 | NM_027756 | 1,32E-03 | -2,77 |
| Orai1 | 10525464 | NM_175423 | 4,50E-04 | -2,77 |
| Gm9953 | 10544717 | ENSMUST00000067831 | 3,94E-03 | -2,76 |
| Rnf43 | 10380116 | NM_172448 | 3,31E-04 | -2,76 |
| Tgfb2 | 10360920 | NM_009367 | 6,03E-04 | -2,76 |
| Tmem56 | 10501802 | NM_178936 | 3,29E-03 | -2,76 |
| Egf | 10502105 | NM_010113 | 6,08E-04 | -2,75 |
| Hsd17b7 | 10359917 | NM_010476 | 5,60E-04 | -2,75 |
| Nov | 10424119 | NM_010930 | 2,42E-03 | -2,75 |
| Tchh | 10493984 | NM_001163098 | 5,46E-03 | -2,75 |
| Tpd52 | 10497214 | NM_001025261 | 1,15E-03 | -2,75 |
| 2810032G03Rik | 10394389 | ENSMUST00000037953 | 1,79E-03 | -2,74 |
| Galnt3 | 10483249 | NM_015736 | 1,50E-03 | -2,74 |
| Sectm1a | 10394068 | NM_145373 | 1,11E-03 | -2,74 |
| Tbc1d13 | 10470936 | NM_146252 | 2,43E-04 | -2,74 |
| Arfgap3 | 10430974 | NM_025445 | 4,60E-04 | -2,73 |
| Steap1 | 10528015 | NM_027399 | 4,98E-04 | -2,73 |
| Ankrd33b | 10428004 | NM_026153 | 1,63E-03 | -2,72 |
| Mfsd7c | 10397364 | NM_145447 | 1,53E-02 | -2,72 |
| Esrp2 | 10581455 | NM_176838 | 3,49E-04 | -2,71 |
| Fam18b | 10376929 | BC115504 | 6,79E-04 | -2,71 |
| Myo5c | 10587150 | NM_001081322 | 1,93E-03 | -2,71 |
| Slc11a2 | 10432573 | NM_001146161 | 2,77E-04 | -2,71 |
| Serp1 | 10498319 | NM_030685 | 1,65E-03 | -2,7 |
| Exph5 | 10585331 | NM_176846 | 1,78E-03 | -2,69 |
| Fam110a | 10488589 | NM_028666 | 2,63E-03 | -2,69 |
| Ncs1 | 10471129 | NM_019681 | 1,70E-02 | -2,69 |
| Sdr42e1 | 10582069 | NM_028725 | 1,17E-03 | -2,69 |
| Spata13 | 10415725 | NM_001033272 | 2,39E-04 | -2,69 |
| Tpd52l1 | 10368566 | NM_009413 | 3,49E-04 | -2,69 |
| Amt | 10588883 | NM_001013814 | 4,07E-04 | -2,68 |
| Atp12a | 10415446 | NM_138652 | 1,80E-02 | -2,68 |
| Cldn8 | 10440647 | NM_018778 | 3,00E-03 | -2,68 |
| Pdxk | 10370497 | NM_172134 | 1,12E-03 | -2,68 |
| Rspo1 | 10508012 | NM_138683 | 2,37E-03 | -2,68 |
| Myo5b | 10456653 | NM_201600 | 5,18E-04 | -2,67 |
| Sec23b | 10476702 | NM_019787 | 1,57E-03 | -2,67 |
| Tspan33 | 10536908 | NM_146173 | 2,83E-03 | -2,67 |
| Amigo2 | 10431935 | NM_178114 | 1,76E-03 | -2,66 |
| D630013G24Rik | 10502273 | ENSMUST00000054105 | 1,87E-02 | -2,66 |
| Nans | 10504743 | NM_053179 | 1,62E-03 | -2,65 |
| Fam134b | 10423333 | NM_001034851 | 1,26E-03 | -2,64 |
| Bcl2l15 | 10494972 | NM_001142959 | 4,93E-03 | -2,63 |
| Cmpk2 | 10395039 | NM_020557 | 1,14E-03 | -2,63 |
| Hs3st3b1 | 10386951 | NM_018805 | 8,52E-04 | -2,63 |
| Lrrc26 | 10469984 | NM_146117 | 9,23E-04 | -2,63 |
| Vopp1 | 10545014 | NM_146168 | 2,25E-04 | -2,63 |
| Btn1a1 | 10408175 | NM_013483 | 1,56E-03 | -2,62 |
| Hook1 | 10506004 | NM_030014 | 1,37E-02 | -2,61 |
| Klc3 | 10560491 | NM_146182 | 8,57E-04 | -2,61 |
| Gm5458 | 10417458 | NM_001024706 | 1,98E-02 | -2,6 |
| Gnb4 | 10497689 | NM_013531 | 7,11E-04 | -2,6 |
| Mast4 | 10411804 | NM_175171 | 2,71E-03 | -2,6 |
| 100043387 | 10479195 | NM_001099327 | 4,47E-03 | -2,59 |
| Dab2 | 10422728 | NM_023118 | 7,11E-04 | -2,59 |
| Dusp4 | 10571312 | NM_176933 | 4,46E-03 | -2,59 |
| Gm14430 | 10479198 | NM_001100415 | 4,47E-03 | -2,59 |
| OTTMUSG00000016609 | 10479192 | NM_001100416 | 4,47E-03 | -2,59 |
| Slc20a1 | 10475990 | NM_015747 | 1,15E-03 | -2,59 |
| Agr2 | 10395365 | NM_011783 | 1,12E-03 | -2,57 |
| Sema4d | 10409240 | NM_013660 | 4,46E-04 | -2,57 |
| Sema4g | 10463430 | NM_011976 | 1,07E-03 | -2,57 |
| Ttc25 | 10381140 | NM_028918 | 3,68E-03 | -2,57 |
| Xdh | 10452815 | NM_011723 | 4,23E-04 | -2,57 |
| Mocs1 | 10445826 | NM_020042 | 8,36E-04 | -2,56 |
| Tlr2 | 10498992 | NM_011905 | 1,68E-03 | -2,55 |
| Ugcg | 10505187 | NM_011673 | 7,05E-04 | -2,55 |
| Rell1 | 10530130 | NM_145923 | 2,42E-03 | -2,54 |
| Tnfrsf11a | 10349051 | NM_009399 | 9,74E-04 | -2,54 |
| Cpox | 10436392 | NM_007757 | 2,97E-04 | -2,53 |
| Fchsd2 | 10555438 | NM_199012 | 6,31E-03 | -2,53 |
| Rab11fip1 | 10577954 | NM_001080813 | 5,16E-04 | -2,53 |
| Sec14l2 | 10383819 | NM_144520 | 1,84E-03 | -2,53 |
| 1700012B07Rik | 10392476 | NM_027038 | 8,30E-04 | -2,52 |
| Ell2 | 10406254 | NM_138953 | 2,34E-03 | -2,52 |
| Pafah2 | 10508974 | NM_133880 | 2,63E-04 | -2,52 |
| 1810058N15Rik | 10460621 | ENSMUST00000058632 | 3,48E-03 | -2,51 |
| Grhl1 | 10394954 | NM_001161406 | 1,30E-03 | -2,51 |
| Gm10565 | 10510047 | NR_027137 | 2,02E-03 | -2,5 |
| Ppm1j | 10495042 | NM_027982 | 5,19E-04 | -2,5 |
| Acnat1 | 10512892 | NM_001164565 | 2,64E-03 | -2,48 |
| Acss1 | 10488482 | NM_080575 | 5,46E-04 | -2,48 |
| Slc41a1 | 10349711 | NM_173865 | 2,00E-03 | -2,48 |
| Cgn | 10500021 | NM_001037711 | 1,41E-03 | -2,47 |
| Fpgs | 10481654 | NM_010236 | 1,19E-03 | -2,47 |
| Mcfd2 | 10453436 | NM_176808 | 8,30E-04 | -2,47 |
| Pfkl | 10370376 | NM_008826 | 1,36E-03 | -2,47 |
| Dcxr | 10393926 | NM_026428 | 2,47E-03 | -2,46 |
| St3gal4 | 10592084 | NM_009178 | 1,86E-03 | -2,46 |
| Btnl5 | 10450189 | NR_004051 | 3,35E-03 | -2,45 |
| Pik3c2g | 10542477 | NM_207683 | 7,06E-03 | -2,45 |
| Slc22a23 | 10408616 | NM_001033167 | 3,49E-04 | -2,45 |
| Slc25a16 | 10363563 | NM_175194 | 9,72E-04 | -2,45 |
| Vdr | 10432032 | NM_009504 | 9,10E-04 | -2,45 |
| H47 | 10554005 | NM_024439 | 6,30E-04 | -2,44 |
| Osbpl7 | 10380739 | NM_001081434 | 5,05E-04 | -2,44 |
| Pctp | 10389775 | NM_008796 | 1,70E-03 | -2,44 |
| Gm2897 | 10417315 | NM_001177714 | 1,26E-02 | -2,43 |
| Gm3002 | 10417235 | NR_033388 | 1,26E-02 | -2,43 |
| Ap1m2 | 10591522 | NM_001110300 | 1,27E-03 | -2,42 |
| Arid5a | 10345445 | NM_001172205 | 9,00E-04 | -2,42 |
| Bglap-rs1 | 10499354 | NM_031368 | 4,41E-03 | -2,42 |
| Reep6 | 10364784 | NM_139292 | 1,13E-03 | -2,42 |
| Secisbp2l | 10487154 | NM_177608 | 9,69E-04 | -2,42 |
| Cd82 | 10485213 | NM_007656 | 8,36E-04 | -2,41 |
| Crb3 | 10446224 | NM_177638 | 3,17E-03 | -2,4 |
| Tec | 10530536 | NM_001113460 | 6,97E-03 | -2,4 |
| Mgat2 | 10396074 | NM_146035 | 5,88E-04 | -2,39 |
| Slc39a11 | 10392701 | NM_001166503 | 8,43E-04 | -2,39 |
| Tmem64 | 10503410 | NM_181401 | 4,89E-04 | -2,39 |
| Awat2 | 10605929 | NM_177746 | 1,43E-03 | -2,37 |
| Cmtm8 | 10597470 | NM_027294 | 6,83E-04 | -2,37 |
| Mpped2 | 10474361 | NM_029837 | 1,36E-03 | -2,37 |
| Ufc1 | 10360120 | NM_025388 | 7,82E-04 | -2,37 |
| Fdx1 | 10593483 | NM_007996 | 7,74E-03 | -2,36 |
| Kcnk1 | 10576581 | NM_008430 | 4,11E-04 | -2,36 |
| Prkaa2 | 10514779 | NM_178143 | 5,65E-03 | -2,36 |
| Rab3ip | 10372583 | NM_001003950 | 5,79E-04 | -2,36 |
| Tbc1d8 | 10354168 | NM_018775 | 1,44E-03 | -2,36 |
| Aldh18a1 | 10467470 | NM_019698 | 9,65E-04 | -2,35 |
| Dyrk3 | 10357590 | NM_145508 | 1,13E-03 | -2,35 |
| Irx2 | 10406005 | NM_010574 | 2,87E-04 | -2,35 |
| Osbpl10 | 10589940 | NM_148958 | 3,28E-04 | -2,35 |
| Frrs1 | 10495596 | NM_001113478 | 5,49E-04 | -2,34 |
| Gpr110 | 10445251 | NM_133776 | 1,05E-02 | -2,34 |
| Pstpip2 | 10456904 | NM_013831 | 2,52E-03 | -2,34 |
| Sigirr | 10569024 | NM_023059 | 3,69E-03 | -2,34 |
| Xbp1 | 10374035 | NM_013842 | 5,35E-04 | -2,34 |
| Tuba4a | 10355806 | NM_009447 | 6,22E-03 | -2,33 |
| Kif26b | 10352143 | NM_001161665 | 8,51E-04 | -2,32 |
| Rnf128 | 10602009 | NM_023270 | 2,18E-02 | -2,32 |
| Slc2a9 | 10529671 | NM_001102414 | 7,27E-04 | -2,32 |
| Apobec3 | 10425333 | ENSMUST00000100423 | 8,60E-03 | -2,31 |
| Cd3g | 10593015 | NM_009850 | 9,35E-03 | -2,31 |
| Slc46a3 | 10535841 | NM_027872 | 6,30E-04 | -2,31 |
| Trim2 | 10499045 | NM_030706 | 4,99E-03 | -2,31 |
| 2210411K11Rik | 10559673 | NM_029384 | 9,10E-03 | -2,3 |
| 6430548M08Rik | 10575993 | NM_172286 | 1,83E-03 | -2,3 |
| C630004H02Rik | 10392910 | BC024617 | 4,78E-03 | -2,3 |
| Elf5 | 10474171 | NM_010125 | 6,08E-04 | -2,3 |
| Fam3c | 10543319 | NM_138587 | 3,08E-03 | -2,3 |
| Gdf5 | 10488954 | NM_008109 | 7,48E-03 | -2,3 |
| Gm10864 | 10425263 | ENSMUST00000100456 | 6,23E-03 | -2,3 |
| Hs6st1 | 10345442 | NM_015818 | 3,34E-03 | -2,3 |
| Ippk | 10405013 | NM_199056 | 6,83E-04 | -2,3 |
| Kcnk5 | 10417798 | NM_021542 | 1,96E-03 | -2,3 |
| Lrrc8b | 10523758 | NM_001033550 | 8,48E-04 | -2,3 |
| Mfsd6 | 10354506 | NM_133829 | 6,79E-04 | -2,3 |
| Mon1a | 10588722 | NM_028369 | 5,35E-04 | -2,3 |
| Slc27a4 | 10470751 | NM_011989 | 1,44E-03 | -2,3 |
| Stat5a | 10381172 | NM_011488 | 2,62E-03 | -2,29 |
| Acbd7 | 10468945 | NM_030063 | 8,43E-04 | -2,28 |
| Golga7 | 10570955 | NM_020585 | 1,77E-02 | -2,28 |
| Muc20 | 10439087 | NM_146071 | 1,71E-03 | -2,28 |
| Pip5k1b | 10466779 | NM_008846 | 2,45E-03 | -2,28 |
| Tdgf1 | 10597268 | NM_011562 | 2,08E-02 | -2,28 |
| Atp6v0a2 | 10525804 | NM_011596 | 3,49E-04 | -2,27 |
| Igsf5 | 10437195 | NM_001177887 | 1,18E-03 | -2,27 |
| Tmem102 | 10387648 | NM_001033433 | 1,96E-03 | -2,27 |
| 1700001L05Rik | 10430993 | NR_027980 | 1,21E-03 | -2,26 |
| Acy1 | 10596465 | NM_025371 | 2,62E-03 | -2,26 |
| Barx2 | 10592050 | NM_013800 | 1,74E-03 | -2,26 |
| D17Wsu104e | 10451993 | NM_080837 | 4,34E-03 | -2,26 |
| Gfod2 | 10581336 | NM_027469 | 2,30E-03 | -2,26 |
| Pim1 | 10443527 | NM_008842 | 6,96E-04 | -2,26 |
| Ssr4 | 10600301 | NM_001166480 | 1,04E-03 | -2,26 |
| BC107364 | 10500347 | BC107364 | 5,39E-03 | -2,25 |
| Pitpnm3 | 10388109 | NM_001024927 | 3,78E-03 | -2,25 |
| Tle6 | 10371240 | NM_053254 | 5,67E-03 | -2,25 |
| Dpf3 | 10401359 | NM_058212 | 7,11E-04 | -2,24 |
| Gm10406 | 10417226 | NM_001164727 | 4,24E-02 | -2,24 |
| Mtap7 | 10361956 | NM_008635 | 6,17E-04 | -2,24 |
| Pold3 | 10565862 | NM_133692 | 7,82E-04 | -2,24 |
| Srd5a3 | 10522589 | NM_020611 | 8,05E-04 | -2,24 |
| Gk5 | 10587988 | NM_177352 | 5,03E-04 | -2,23 |
| Sh3bp5 | 10418702 | NM_011894 | 1,48E-03 | -2,23 |
| Usp53 | 10501879 | NM_133857 | 4,26E-02 | -2,23 |
| Fam115c | 10544452 | BC011487 | 4,63E-04 | -2,22 |
| Slc5a5 | 10579442 | NM_053248 | 1,87E-03 | -2,22 |
| Dusp8 | 10569280 | ENSMUST00000039926 | 9,44E-03 | -2,21 |
| Psg25 | 10560375 | NM_054060 | 3,79E-03 | -2,21 |
| Pycard | 10568355 | NM_023258 | 1,61E-03 | -2,21 |
| Sec61b | 10426889 | NM_024171 | 3,14E-02 | -2,21 |
| Arfgef2 | 10478778 | NM_001085495 | 5,19E-04 | -2,2 |
| Cldn7 | 10377673 | NM_016887 | 2,98E-03 | -2,2 |
| Ctse | 10349648 | NM_007799 | 2,57E-02 | -2,2 |
| Llgl2 | 10382625 | NM_145438 | 4,21E-03 | -2,2 |
| Oxct1 | 10422608 | NM_024188 | 1,07E-03 | -2,2 |
| Phgdh | 10500529 | NM_016966 | 1,01E-02 | -2,2 |
| Rasef | 10513943 | ENSMUST00000102837 | 5,44E-04 | -2,2 |
| Sec16b | 10350864 | NM_033354 | 1,94E-03 | -2,2 |
| Tbc1d14 | 10529549 | NM_001113362 | 5,06E-04 | -2,2 |
| Tsta3 | 10429674 | NM_031201 | 2,13E-03 | -2,2 |
| Susd3 | 10409162 | NM_025491 | 4,33E-03 | -2,19 |
| Tmem82 | 10517980 | NM_145987 | 8,26E-03 | -2,19 |
| Chrnb2 | 10499643 | NM_009602 | 1,67E-03 | -2,18 |
| Gusb | 10534102 | NM_010368 | 2,85E-03 | -2,18 |
| Pcsk7 | 10584954 | NM_008794 | 4,35E-04 | -2,18 |
| Rbm47 | 10530269 | NM_178446 | 2,06E-03 | -2,18 |
| Slc39a14 | 10421309 | NM_001135151 | 5,88E-04 | -2,18 |
| Uck2 | 10359849 | ENSMUST00000053686 | 8,16E-03 | -2,18 |
| 1110008P14Rik | 10481621 | NM_198001 | 9,76E-03 | -2,17 |
| Bdh1 | 10434934 | NM_175177 | 2,52E-03 | -2,17 |
| Klhl29 | 10399337 | NM_001164493 | 6,52E-03 | -2,17 |
| Nlrp9b | 10550749 | NM_194058 | 2,76E-02 | -2,17 |
| Smox | 10476301 | NM_001177833 | 3,74E-03 | -2,17 |
| St6galnac2 | 10393166 | NM_009180 | 2,12E-03 | -2,17 |
| Cadm4 | 10550915 | NM_153112 | 9,44E-03 | -2,16 |
| Entpd7 | 10463282 | NM_053103 | 6,72E-03 | -2,16 |
| Gm10417 | 10524060 | ENSMUST00000100939 | 1,89E-02 | -2,16 |
| Ltf | 10589703 | NM_008522 | 1,94E-02 | -2,16 |
| Pdzk1ip1 | 10507137 | NM_001164557 | 3,50E-03 | -2,16 |
| Radil | 10535312 | NM_178702 | 1,63E-03 | -2,16 |
| Tob1 | 10380381 | NM_009427 | 6,66E-04 | -2,16 |
| Rassf3 | 10372844 | NM_138956 | 2,19E-03 | -2,15 |
| A2ld1 | 10422512 | NM_145466 | 9,00E-04 | -2,14 |
| Esco1 | 10407040 | NM_001081222 | 4,67E-02 | -2,14 |
| Gm3696 | 10417421 | NM_001024712 | 3,67E-02 | -2,14 |
| Rab11fip4 | 10379321 | NM_175543 | 3,09E-03 | -2,13 |
| Rnf145 | 10375343 | NM_028862 | 1,59E-03 | -2,13 |
| Slc39a7 | 10450116 | NM_008202 | 3,26E-03 | -2,13 |
| Snd1 | 10536762 | NM_019776 | 1,23E-03 | -2,13 |
| Abhd5 | 10590452 | NM_026179 | 8,36E-04 | -2,12 |
| Mfsd11 | 10382852 | NM_178620 | 1,41E-03 | -2,12 |
| Prdx4 | 10607475 | NM_016764 | 3,99E-03 | -2,12 |
| Sgsm3 | 10425477 | NM_134091 | 1,51E-03 | -2,12 |
| 1700026D08Rik | 10565241 | NM_029335 | 3,50E-03 | -2,11 |
| 4930506M07Rik | 10468762 | NM_001114312 | 1,53E-02 | -2,11 |
| Gpd1l | 10597477 | NM_175380 | 6,29E-04 | -2,11 |
| Pfkfb2 | 10357535 | NM_008825 | 7,00E-03 | -2,11 |
| Slc5a6 | 10529052 | NM_001177621 | 8,40E-03 | -2,11 |
| Tmem51 | 10518108 | NM_145402 | 4,79E-03 | -2,11 |
| Tnik | 10491136 | NM_026910 | 4,00E-03 | -2,11 |
| Ttll7 | 10496837 | NM_027594 | 4,90E-03 | -2,11 |
| Agpat6 | 10577604 | NM_018743 | 8,00E-04 | -2,1 |
| Entpd3 | 10590306 | NM_178676 | 4,73E-03 | -2,1 |
| Rab4a | 10576391 | NM_009003 | 9,26E-04 | -2,1 |
| Rhou | 10576386 | NM_133955 | 1,56E-03 | -2,1 |
| Tmem44 | 10438942 | NM_172614 | 1,33E-03 | -2,1 |
| Zdhhc9 | 10604380 | NM_172465 | 1,01E-03 | -2,1 |
| Aqp9 | 10594825 | NM_022026 | 1,99E-02 | -2,09 |
| Efhd2 | 10518069 | NM_025994 | 1,63E-03 | -2,09 |
| Glrx | 10406270 | NM_053108 | 1,71E-03 | -2,09 |
| Ipcef1 | 10361338 | NM_001033391 | 9,44E-03 | -2,09 |
| Man1c1 | 10517287 | NM_207237 | 1,21E-03 | -2,09 |
| Mrs2 | 10408348 | NM_001013389 | 8,00E-04 | -2,09 |
| Srm | 10510391 | NM_009272 | 4,69E-03 | -2,09 |
| Clint1 | 10375382 | NM_001045520 | 1,19E-03 | -2,08 |
| Ctnnal1 | 10513061 | NM_018761 | 3,81E-03 | -2,08 |
| Elovl1 | 10507539 | NM_019422 | 1,60E-03 | -2,08 |
| Ildr1 | 10435514 | NM_134109 | 7,86E-04 | -2,08 |
| Tirap | 10592106 | NM_001177845 | 1,23E-03 | -2,08 |
| Atp2c1 | 10596347 | NM_175025 | 2,57E-03 | -2,07 |
| Cdhr4 | 10588819 | NM_001122635 | 2,62E-03 | -2,07 |
| Dap | 10423498 | NM_146057 | 8,43E-04 | -2,07 |
| Iars | 10405094 | NM_172015 | 9,87E-04 | -2,07 |
| Opn1mw | 10600317 | NM_008106 | 1,78E-03 | -2,07 |
| Cited4 | 10507726 | NM_019563 | 6,61E-03 | -2,06 |
| Cobl | 10384423 | NM_172496 | 4,66E-03 | -2,06 |
| Ctns | 10388241 | NM_031251 | 1,19E-03 | -2,06 |
| Fam108c | 10565288 | NM_133722 | 1,47E-03 | -2,06 |
| Gm9983 | 10408346 | ENSMUST00000069594 | 6,82E-03 | -2,06 |
| Slc39a6 | 10457872 | NM_139143 | 6,08E-04 | -2,06 |
| Zbtb42 | 10398881 | NM_001100460 | 8,34E-03 | -2,06 |
| Gm6742 | 10399228 | XR_034503 | 6,84E-03 | -2,05 |
| Impdh1 | 10543572 | NM_011829 | 7,20E-03 | -2,05 |
| Shisa7 | 10559681 | NM_172737 | 3,56E-03 | -2,05 |
| Slc2a1 | 10507594 | NM_011400 | 2,66E-02 | -2,05 |
| 0610040J01Rik | 10522004 | BC004797 | 1,54E-03 | -2,04 |
| E130012A19Rik | 10390454 | BC055770 | 2,84E-02 | -2,04 |
| Fam63b | 10594802 | NM_172772 | 8,03E-03 | -2,04 |
| Krt15 | 10391025 | NM_008469 | 2,51E-02 | -2,04 |
| Olfr1519 | 10515834 | NM_146399 | 4,20E-03 | -2,04 |
| Plxnb3 | 10600249 | NM_019587 | 1,34E-03 | -2,04 |
| Susd1 | 10513362 | NM_001163288 | 1,57E-03 | -2,04 |
| Traf6 | 10474112 | NM_009424 | 1,99E-03 | -2,04 |
| Dhodh | 10581612 | NM_020046 | 1,60E-03 | -2,03 |
| Galnt7 | 10578771 | NM_144731 | 6,08E-03 | -2,03 |
| Slc17a5 | 10595189 | NM_172773 | 7,74E-03 | -2,03 |
| Slc17a9 | 10479463 | NM_183161 | 6,41E-03 | -2,03 |
| Slc35c2 | 10489646 | NM_144893 | 1,01E-02 | -2,03 |
| Tmem30b | 10400984 | NM_178715 | 2,87E-03 | -2,03 |
| Unc13b | 10504234 | NM_001081413 | 2,17E-03 | -2,03 |
| 1810012P15Rik | 10379223 | NM_001076681 | 7,35E-03 | -2,02 |
| Efcab4a | 10558936 | NM_001025103 | 4,74E-03 | -2,02 |
| Ero1lb | 10403558 | NM_026184 | 2,63E-03 | -2,02 |
| Isg20 | 10554240 | NM_020583 | 1,89E-03 | -2,02 |
| Nhp2 | 10375880 | NM_026631 | 3,68E-03 | -2,02 |
| Slc29a4 | 10527148 | NM_146257 | 4,74E-03 | -2,02 |
| Slc43a2 | 10378649 | NM_173388 | 2,46E-03 | -2,02 |
| Stim2 | 10521950 | NM_001081103 | 9,88E-04 | -2,02 |
| Uba5 | 10596207 | NM_025692 | 1,63E-03 | -2,02 |
| Cachd1 | 10506225 | NM_198037 | 3,31E-03 | -2,01 |
| Chka | 10460221 | NM_013490 | 1,18E-02 | -2,01 |
| Hsd17b2 | 10575833 | NM_008290 | 1,08E-03 | -2,01 |
| Naaladl2 | 10497501 | XM_910834 | 3,78E-02 | -2,01 |
| Nt5dc3 | 10365518 | NM_175331 | 8,80E-04 | -2,01 |
| Pdhb | 10417538 | NM_024221 | 3,97E-03 | -2,01 |
| Plekhf1 | 10562576 | NM_024413 | 9,00E-03 | -2,01 |
| St14 | 10592001 | NM_011176 | 2,17E-03 | -2,01 |
| 4930451G09Rik | 10433340 | ENSMUST00000090457 | 2,71E-03 | -2 |
| Dnajc6 | 10506274 | NM_001164585 | 1,18E-03 | -2 |
| Gnpnat1 | 10460108 | NM_019425 | 3,30E-03 | -2 |
| Ifne | 10514335 | NM_177348 | 3,60E-02 | -2 |
| Mlx | 10381238 | NM_011550 | 6,26E-04 | -2 |
| Prok1 | 10501084 | NM_001044382 | 1,76E-03 | -2 |
| Sidt2 | 10593130 | NM_172257 | 9,04E-04 | -2 |
| Trabd | 10426157 | NM_026485 | 5,79E-03 | -2 |
| Xk | 10598586 | NM_023500 | 2,64E-03 | -2 |
| 4930402H24Rik | 10487748 | BC052447 | 5,84E-03 | 2 |
| Adamts3 | 10531189 | NM_177872 | 1,99E-03 | 2 |
| Aldh3b1 | 10464560 | NM_026316 | 2,95E-02 | 2 |
| Arhgap31 | 10439483 | NM_020260 | 6,84E-03 | 2 |
| Bnip3 | 10414269 | NM_009760 | 2,69E-02 | 2 |
| Cdc14a | 10501629 | NM_001080818 | 4,71E-03 | 2 |
| Celsr1 | 10431229 | NM_009886 | 4,24E-03 | 2 |
| Iffo1 | 10541862 | NM_178787 | 5,27E-03 | 2 |
| Itgax | 10557895 | NM_021334 | 4,68E-02 | 2 |
| Kank3 | 10444028 | NM_030697 | 5,84E-03 | 2 |
| Klf12 | 10422013 | NM_010636 | 1,86E-02 | 2 |
| Lamb1-1 | 10395163 | NM_008482 | 8,61E-03 | 2 |
| Mxra7 | 10393379 | NM_026280 | 1,99E-03 | 2 |
| Nynrin | 10415413 | NM_001040072 | 2,35E-02 | 2 |
| Oas1b | 10525158 | NR_003507 | 3,86E-02 | 2 |
| P2ry14 | 10498350 | NM_133200 | 4,82E-03 | 2 |
| Pde3a | 10542575 | NM_018779 | 1,91E-02 | 2 |
| Pigz | 10434993 | NM_172822 | 6,24E-03 | 2 |
| Sfpi1 | 10473809 | NM_011355 | 1,06E-02 | 2 |
| Slc16a5 | 10382532 | NM_001080934 | 7,85E-03 | 2 |
| Syne2 | 10396511 | NM_001005510 | 6,84E-04 | 2 |
| Thsd4 | 10594199 | NM_001040426 | 3,10E-03 | 2 |
| 2310014H01Rik | 10444932 | NM_001146711 | 2,77E-02 | 2,01 |
| Alpk1 | 10502042 | NM_027808 | 2,30E-03 | 2,01 |
| B4galt5 | 10489891 | NM_019835 | 1,39E-02 | 2,01 |
| Begain | 10402598 | NM_001163175 | 2,39E-03 | 2,01 |
| Capn2 | 10360806 | NM_009794 | 3,87E-03 | 2,01 |
| D14Ertd668e | 10420488 | NM_001164323 | 9,65E-03 | 2,01 |
| Dock10 | 10356020 | NM_175291 | 5,17E-03 | 2,01 |
| Dock6 | 10591616 | NM_177030 | 5,61E-03 | 2,01 |
| Dpysl2 | 10420988 | NM_009955 | 5,24E-03 | 2,01 |
| Fabp5 | 10490838 | NM_010634 | 2,89E-02 | 2,01 |
| Fam101b | 10388488 | NM_029658 | 5,84E-03 | 2,01 |
| Fam171b | 10473312 | NM_175514 | 1,24E-02 | 2,01 |
| Fam65b | 10404152 | NM_029679 | 3,08E-03 | 2,01 |
| Fmr1 | 10599893 | NM_008031 | 2,94E-02 | 2,01 |
| Fxyd5 | 10562192 | NM_008761 | 4,00E-03 | 2,01 |
| Gem | 10503334 | NM_010276 | 8,26E-03 | 2,01 |
| Hpgds | 10545101 | NM_019455 | 1,73E-02 | 2,01 |
| Il17rd | 10413398 | NM_134437 | 8,00E-04 | 2,01 |
| Nol3 | 10574676 | NM_030152 | 4,22E-02 | 2,01 |
| Ptgs1 | 10471721 | NM_008969 | 4,74E-03 | 2,01 |
| Sfxn4 | 10468853 | NM_053198 | 2,90E-02 | 2,01 |
| Syne1 | 10361381 | NM_001079686 | 2,43E-03 | 2,01 |
| Synpo | 10459084 | NM_001109975 | 5,50E-03 | 2,01 |
| Adamts14 | 10369431 | NM_001081127 | 2,48E-02 | 2,02 |
| AF529169 | 10595657 | AF529169 | 1,62E-03 | 2,02 |
| Bcat1 | 10549222 | NM_001024468 | 3,93E-02 | 2,02 |
| Ccdc109b | 10502156 | NM_025779 | 7,35E-03 | 2,02 |
| Ifitm1 | 10570434 | NM_026820 | 4,59E-03 | 2,02 |
| Lcp2 | 10375145 | NM_010696 | 2,09E-02 | 2,02 |
| Lmo1 | 10566723 | NM_057173 | 9,07E-03 | 2,02 |
| Lphn3 | 10522749 | NM_198702 | 1,95E-03 | 2,02 |
| Mafb | 10489246 | NM_010658 | 7,28E-03 | 2,02 |
| Picalm | 10554839 | NM_146194 | 1,17E-03 | 2,02 |
| Sepw1 | 10560174 | NM_009156 | 1,86E-02 | 2,02 |
| Socs2 | 10372069 | NM_007706 | 3,00E-03 | 2,02 |
| Spns3 | 10388211 | NM_029932 | 1,08E-02 | 2,02 |
| Spred1 | 10474671 | NM_033524 | 1,10E-03 | 2,02 |
| Ube2l6 | 10473356 | NM_019949 | 2,68E-02 | 2,02 |
| Vamp1 | 10541877 | NM_001080557 | 1,99E-02 | 2,02 |
| Vwf | 10541910 | NM_011708 | 1,45E-02 | 2,02 |
| Zc3hav1l | 10544084 | NM_172467 | 1,30E-03 | 2,02 |
| Bex1 | 10606868 | NM_009052 | 5,21E-03 | 2,03 |
| Chadl | 10430745 | NM_001164320 | 2,75E-02 | 2,03 |
| Col4a2 | 10570068 | NM_009932 | 2,43E-02 | 2,03 |
| Fdft1 | 10412909 | NM_010191 | 1,05E-02 | 2,03 |
| Gm13375 | 10469609 | NR_033225 | 1,18E-02 | 2,03 |
| Il1rl1 | 10345791 | NM_001025602 | 1,63E-03 | 2,03 |
| Ly6a | 10429564 | NM_010738 | 5,70E-03 | 2,03 |
| Mir497 | 10377800 | NR_030444 | 5,80E-03 | 2,03 |
| Rap1gap | 10509410 | NM_001081155 | 4,45E-03 | 2,03 |
| Stx2 | 10534041 | NM_007941 | 5,97E-03 | 2,03 |
| Tk1 | 10582809 | NM_009387 | 1,24E-02 | 2,03 |
| Vnn3 | 10362129 | NM_011979 | 3,64E-02 | 2,03 |
| Wdfy3 | 10531796 | NM_172882 | 7,82E-04 | 2,03 |
| Zfp334 | 10489694 | NM_178411 | 5,54E-03 | 2,03 |
| Atp2b4 | 10357833 | NM_001167949 | 4,55E-03 | 2,04 |
| Btd | 10413803 | NM_025295 | 4,37E-03 | 2,04 |
| Evi5l | 10569898 | NM_001039578 | 6,63E-04 | 2,04 |
| Fhl5 | 10511835 | NM_021318 | 2,43E-03 | 2,04 |
| Gxylt2 | 10540275 | NM_198612 | 9,72E-03 | 2,04 |
| Il1rn | 10469816 | NM_031167 | 1,32E-02 | 2,04 |
| Mustn1 | 10413609 | NM_181390 | 4,59E-03 | 2,04 |
| Phf15 | 10385747 | NM_199299 | 2,18E-02 | 2,04 |
| Prnp | 10476314 | NM_011170 | 2,15E-02 | 2,04 |
| Rcn1 | 10485645 | NM_009037 | 2,14E-03 | 2,04 |
| Sgk1 | 10362073 | NM_001161845 | 1,79E-03 | 2,04 |
| Sqrdl | 10475532 | NM_021507 | 2,22E-02 | 2,04 |
| Tmem140 | 10537227 | NM_197986 | 1,36E-02 | 2,04 |
| Tpst1 | 10526120 | NM_001130476 | 1,05E-02 | 2,04 |
| Vsig2 | 10584325 | NM_020518 | 2,04E-03 | 2,04 |
| Zfp600 | 10510191 | NM_001177546 | 1,18E-03 | 2,04 |
| 37865 | 10425726 | NM_011889 | 3,68E-03 | 2,05 |
| Adamtsl1 | 10505717 | NM_029967 | 1,46E-02 | 2,05 |
| Cercam | 10470775 | NM_207298 | 9,29E-03 | 2,05 |
| Coro1c | 10532753 | NM_011779 | 6,83E-03 | 2,05 |
| Csprs | 10356262 | NM_033616 | 1,66E-02 | 2,05 |
| Dock2 | 10385118 | NM_033374 | 1,49E-02 | 2,05 |
| Dse | 10368647 | NM_172508 | 6,47E-03 | 2,05 |
| Ephb6 | 10537657 | NM_001146351 | 2,03E-03 | 2,05 |
| Gm13051 | 10490854 | NM_001037926 | 1,41E-02 | 2,05 |
| Gucy1a2 | 10582958 | NM_001033322 | 1,69E-03 | 2,05 |
| Mst1r | 10588731 | NM_009074 | 4,04E-02 | 2,05 |
| Nt5e | 10587639 | NM_011851 | 2,44E-02 | 2,05 |
| Plxna2 | 10352867 | NM_008882 | 5,58E-03 | 2,05 |
| Rhbdl1 | 10449061 | NM_144816 | 1,80E-02 | 2,05 |
| Scn2a1 | 10472400 | NM_001099298 | 4,51E-02 | 2,05 |
| Slitrk4 | 10604853 | NM_178740 | 2,36E-03 | 2,05 |
| Stard8 | 10600921 | NM_199018 | 1,87E-03 | 2,05 |
| Wisp1 | 10424543 | NM_018865 | 6,36E-03 | 2,05 |
| Ap1s2 | 10603051 | NM_026887 | 1,18E-02 | 2,06 |
| Fam70b | 10570388 | NM_001143671 | 2,07E-02 | 2,06 |
| Gm10851 | 10469276 | ENSMUST00000100409 | 1,31E-03 | 2,06 |
| Lrrc49 | 10594221 | NM_145616 | 1,40E-03 | 2,06 |
| Nceh1 | 10491083 | NM_178772 | 1,65E-02 | 2,06 |
| Opcml | 10584024 | NM_177906 | 2,64E-02 | 2,06 |
| Plau | 10413047 | NM_008873 | 1,14E-02 | 2,06 |
| Ptprm | 10452571 | NM_008984 | 3,97E-03 | 2,06 |
| Sox18 | 10490731 | NM_009236 | 1,73E-02 | 2,06 |
| St3gal2 | 10575534 | NM_009179 | 1,31E-02 | 2,06 |
| Ttc28 | 10524312 | NM_024477 | 1,23E-02 | 2,06 |
| Vmn1r34 | 10545117 | NM_001166719 | 3,19E-02 | 2,06 |
| Alpl | 10517587 | NM_007431 | 1,02E-02 | 2,07 |
| Atp8a1 | 10530319 | NM_001038999 | 8,43E-04 | 2,07 |
| Cd38 | 10521678 | NM_007646 | 3,31E-03 | 2,07 |
| Cyyr1 | 10440513 | NM_144853 | 1,87E-03 | 2,07 |
| Fam132a | 10511258 | NM_026125 | 1,86E-02 | 2,07 |
| Fxyd6 | 10584883 | NM_022004 | 4,63E-03 | 2,07 |
| Gpr137b-ps | 10407792 | NR_003568 | 2,85E-03 | 2,07 |
| Hsh2d | 10572772 | NM_197944 | 1,75E-03 | 2,07 |
| Nt5dc2 | 10413710 | NM_027289 | 6,99E-03 | 2,07 |
| Sctr | 10349319 | NM_001012322 | 2,79E-02 | 2,07 |
| Slc38a4 | 10431915 | NM_027052 | 2,18E-02 | 2,07 |
| Srgap1 | 10372891 | NM_001081037 | 1,56E-03 | 2,07 |
| Trim30 | 10566358 | NM_009099 | 4,51E-02 | 2,07 |
| Vmn1r101 | 10560780 | NM_001166836 | 4,70E-02 | 2,07 |
| Apbb1ip | 10469695 | NM_019456 | 3,01E-02 | 2,08 |
| Col6a3 | 10356520 | AF064749 | 4,53E-02 | 2,08 |
| Dennd2d | 10501048 | NM_001093754 | 5,86E-03 | 2,08 |
| Hes1 | 10434925 | NM_008235 | 2,78E-03 | 2,08 |
| Itga4 | 10473125 | NM_010576 | 1,25E-02 | 2,08 |
| Lrrc36 | 10574765 | NM_001170788 | 4,90E-04 | 2,08 |
| Mir350 | 10360504 | NR_029775 | 3,07E-02 | 2,08 |
| Numbl | 10551336 | NM_010950 | 3,20E-02 | 2,08 |
| P2ry13 | 10498367 | NM_028808 | 8,51E-04 | 2,08 |
| Pear1 | 10499216 | NM_028460 | 9,76E-03 | 2,08 |
| Samd14 | 10380489 | NM_146025 | 2,90E-02 | 2,08 |
| Serpinh1 | 10565794 | NM_009825 | 8,28E-03 | 2,08 |
| Speer4e | 10528177 | NM_001122661 | 4,34E-02 | 2,08 |
| Ssh2 | 10378855 | ENSMUST00000037912 | 2,58E-03 | 2,08 |
| St6galnac6 | 10471464 | NM_016973 | 6,97E-03 | 2,08 |
| Adamts17 | 10554063 | NM_001033877 | 2,53E-02 | 2,09 |
| Alox5 | 10547153 | NM_009662 | 4,60E-02 | 2,09 |
| Atp1a4 | 10360248 | NM_013734 | 7,30E-03 | 2,09 |
| Capsl | 10423024 | NM_029341 | 4,21E-03 | 2,09 |
| Cntln | 10505674 | NM_175275 | 1,94E-03 | 2,09 |
| Col18a1 | 10370259 | NM_009929 | 3,30E-02 | 2,09 |
| Cyp4v3 | 10578448 | NM_133969 | 7,92E-03 | 2,09 |
| Cyp7b1 | 10497381 | NM_007825 | 1,59E-02 | 2,09 |
| Dcn | 10365974 | NM_007833 | 3,33E-03 | 2,09 |
| Esam | 10584317 | NM_027102 | 6,24E-03 | 2,09 |
| Mmp23 | 10519140 | NM_011985 | 1,38E-02 | 2,09 |
| Olfml2b | 10351491 | NM_177068 | 1,53E-02 | 2,09 |
| Snx10 | 10538290 | NM_028035 | 4,25E-02 | 2,09 |
| Steap4 | 10519497 | NM_054098 | 1,53E-02 | 2,09 |
| Tns1 | 10355514 | NM_027884 | 1,87E-02 | 2,09 |
| Vegfb | 10465559 | NM_011697 | 2,18E-02 | 2,09 |
| Zfp521 | 10457587 | NM_145492 | 4,21E-03 | 2,09 |
| AB124611 | 10583669 | NM_206536 | 5,59E-03 | 2,1 |
| Acp5 | 10591739 | NM_001102404 | 1,11E-02 | 2,1 |
| B3galnt1 | 10498647 | NM_020026 | 1,08E-02 | 2,1 |
| Cd1d1 | 10499160 | NM_007639 | 3,90E-02 | 2,1 |
| Dlg4 | 10377725 | NM_007864 | 1,58E-03 | 2,1 |
| Evi2a | 10388958 | NM_001033711 | 5,84E-03 | 2,1 |
| Mctp1 | 10406334 | NM_030174 | 1,78E-03 | 2,1 |
| Nfkbie | 10445412 | NM_008690 | 1,11E-02 | 2,1 |
| Rabep1 | 10377987 | NM_019400 | 2,19E-03 | 2,1 |
| Rbm46 | 10498972 | NM_001146328 | 1,40E-03 | 2,1 |
| Stom | 10482030 | NM_013515 | 2,49E-02 | 2,1 |
| Thbs1 | 10474700 | NM_011580 | 3,39E-03 | 2,1 |
| 5330417C22Rik | 10501374 | NM_001033304 | 1,12E-02 | 2,11 |
| Adamts10 | 10443949 | NM_172619 | 1,18E-03 | 2,11 |
| Amotl2 | 10588226 | NM_019764 | 2,79E-03 | 2,11 |
| Angpt2 | 10577315 | NM_007426 | 2,87E-03 | 2,11 |
| Bend4 | 10530310 | NM_001164806 | 5,45E-03 | 2,11 |
| Capn8 | 10352416 | NM_130890 | 1,41E-02 | 2,11 |
| Eltd1 | 10496872 | NM_133222 | 1,31E-03 | 2,11 |
| Ggta1 | 10482059 | NM_010283 | 1,55E-03 | 2,11 |
| Lipe | 10551155 | NM_010719 | 3,52E-02 | 2,11 |
| Lsp1 | 10559207 | NM_019391 | 1,95E-02 | 2,11 |
| Nlgn2 | 10387659 | NM_198862 | 2,51E-03 | 2,11 |
| Rftn2 | 10354741 | NM_028713 | 2,39E-03 | 2,11 |
| Rgs7bp | 10411949 | NM_029879 | 3,36E-03 | 2,11 |
| Zfp189 | 10504918 | NM_145547 | 3,43E-02 | 2,11 |
| 6720489N17Rik | 10409990 | BC053725 | 4,35E-02 | 2,12 |
| Abat | 10433445 | NM_172961 | 2,79E-03 | 2,12 |
| C1qb | 10517508 | NM_009777 | 5,39E-03 | 2,12 |
| Ddx26b | 10599696 | NM_172779 | 1,48E-02 | 2,12 |
| Emp2 | 10437639 | NM_007929 | 6,35E-03 | 2,12 |
| Etv4 | 10391490 | NM_008815 | 2,56E-03 | 2,12 |
| Fbxo21 | 10524941 | NM_145564 | 2,80E-02 | 2,12 |
| Hacl1 | 10418747 | NM_019975 | 2,27E-02 | 2,12 |
| Mmp27 | 10583112 | NM_001030289 | 7,79E-03 | 2,12 |
| Por | 10526363 | NM_008898 | 3,08E-02 | 2,12 |
| Ptprg | 10412667 | NM_008981 | 2,45E-03 | 2,12 |
| Tmem194b | 10346224 | NM_001142647 | 4,59E-03 | 2,12 |
| A530099J19Rik | 10407982 | NM_175688 | 4,17E-03 | 2,13 |
| Cdr2 | 10567564 | NM_007672 | 2,81E-02 | 2,13 |
| Disp2 | 10474814 | NM_170593 | 1,65E-02 | 2,13 |
| Etv5 | 10363901 | NM_023794 | 9,81E-03 | 2,13 |
| Evc | 10529613 | NM_021292 | 3,99E-03 | 2,13 |
| Grasp | 10427026 | NM_019518 | 8,73E-03 | 2,13 |
| Gypc | 10458028 | NM_001048207 | 5,66E-03 | 2,13 |
| Kbtbd11 | 10570516 | NM_029116 | 7,04E-03 | 2,13 |
| Msn | 10600836 | NM_010833 | 1,73E-03 | 2,13 |
| Rnf150 | 10573115 | NM_177378 | 7,96E-04 | 2,13 |
| Tbx3 | 10525016 | NM_011535 | 1,07E-03 | 2,13 |
| Vash2 | 10361055 | NR_027352 | 8,94E-04 | 2,13 |
| Cd33 | 10562709 | NM_001111058 | 6,63E-04 | 2,14 |
| Cds2 | 10476326 | NM_138651 | 4,62E-03 | 2,14 |
| Eng | 10471486 | NM_001146350 | 4,76E-03 | 2,14 |
| Lgmn | 10402268 | NM_011175 | 1,70E-02 | 2,14 |
| Ndn | 10553833 | NM_010882 | 4,63E-02 | 2,14 |
| Nr2f2 | 10564527 | NM_009697 | 5,96E-04 | 2,14 |
| Pld4 | 10398907 | NM_178911 | 2,87E-02 | 2,14 |
| Postn | 10492021 | NM_015784 | 2,58E-03 | 2,14 |
| Ppap2b | 10506488 | NM_080555 | 2,86E-03 | 2,14 |
| Ppargc1a | 10529979 | NR_027710 | 1,58E-03 | 2,14 |
| Rgs12 | 10521337 | NM_173402 | 3,00E-03 | 2,14 |
| Slc7a2 | 10571444 | NM_007514 | 2,03E-03 | 2,14 |
| Tll1 | 10578880 | NM_009390 | 5,86E-03 | 2,14 |
| Wif1 | 10366653 | NM_011915 | 2,48E-02 | 2,14 |
| 38961 | 10604057 | NR_033443 | 1,98E-03 | 2,15 |
| 1700029I01Rik | 10518335 | NM_027285 | 1,24E-02 | 2,15 |
| A930039A15Rik | 10350758 | AK080751 | 8,10E-03 | 2,15 |
| Abhd15 | 10378893 | NM_026185 | 1,47E-02 | 2,15 |
| Akt3 | 10360506 | NM_011785 | 1,22E-03 | 2,15 |
| Bmpr1b | 10502451 | NM_007560 | 8,86E-03 | 2,15 |
| Cyth4 | 10425092 | NM_028195 | 2,63E-02 | 2,15 |
| Hmgn3 | 10595371 | NM_026122 | 5,19E-04 | 2,15 |
| Kcnh1 | 10352798 | NM_010600 | 1,00E-02 | 2,15 |
| Ldhb | 10549097 | NM_008492 | 2,15E-02 | 2,15 |
| Lilrb4 | 10363082 | NM_013532 | 1,51E-02 | 2,15 |
| Pag1 | 10497237 | NM_053182 | 6,83E-04 | 2,15 |
| Pion | 10519951 | NM_175437 | 1,10E-03 | 2,15 |
| Ppp1r1a | 10433163 | NM_021391 | 9,81E-03 | 2,15 |
| Rara | 10381082 | NM_001177302 | 4,11E-03 | 2,15 |
| Scnn1b | 10557124 | NM_011325 | 7,85E-03 | 2,15 |
| Sesn1 | 10362811 | NM_001162908 | 1,62E-03 | 2,15 |
| Shc4 | 10487139 | NM_199022 | 1,52E-03 | 2,15 |
| Slc5a8 | 10365640 | NM_145423 | 1,49E-02 | 2,15 |
| Stx1b | 10568298 | NM_024414 | 8,86E-03 | 2,15 |
| Arhgap24 | 10523579 | NM_029270 | 6,98E-03 | 2,16 |
| Ctdspl | 10590060 | NM_133710 | 6,71E-03 | 2,16 |
| Eno2 | 10547807 | NM_013509 | 7,24E-04 | 2,16 |
| Fam198a | 10590438 | BC113767 | 3,24E-03 | 2,16 |
| Fam92a | 10511617 | NM_026558 | 6,22E-04 | 2,16 |
| Hoxc9 | 10427286 | NM_008272 | 9,75E-03 | 2,16 |
| Ly6d | 10429520 | NM_010742 | 4,44E-03 | 2,16 |
| Meis1 | 10384504 | NM_010789 | 2,50E-03 | 2,16 |
| Ppic | 10458906 | NM_008908 | 9,13E-03 | 2,16 |
| Stab1 | 10418506 | NM_138672 | 4,53E-03 | 2,16 |
| Tgfbr3 | 10532085 | NM_011578 | 5,88E-04 | 2,16 |
| Tspo | 10425808 | NM_009775 | 2,55E-02 | 2,16 |
| Ttc3 | 10437080 | NM_009441 | 3,59E-03 | 2,16 |
| 1200009O22Rik | 10544837 | NM_025817 | 6,09E-04 | 2,17 |
| Akap12 | 10367634 | NM_031185 | 1,57E-02 | 2,17 |
| Als2cr12 | 10354883 | NM_175370 | 9,16E-03 | 2,17 |
| Arhgef17 | 10565935 | NM_001081116 | 6,42E-03 | 2,17 |
| Atp10a | 10553788 | NM_009728 | 7,39E-03 | 2,17 |
| B130024G19Rik | 10554129 | BC070425 | 3,53E-03 | 2,17 |
| Bcl2 | 10357043 | NM_009741 | 3,00E-03 | 2,17 |
| Col6a2 | 10370180 | NM_146007 | 1,52E-02 | 2,17 |
| Fap | 10483081 | NM_007986 | 6,04E-03 | 2,17 |
| Myo9a | 10585976 | NM_173018 | 2,53E-02 | 2,17 |
| Ndp | 10603764 | NM_010883 | 3,66E-03 | 2,17 |
| Pdlim4 | 10385903 | NM_019417 | 4,37E-03 | 2,17 |
| Plat | 10570855 | NM_008872 | 3,56E-02 | 2,17 |
| Ramp2 | 10381298 | NM_019444 | 1,81E-02 | 2,17 |
| Runx1t1 | 10503382 | NM_001111027 | 9,04E-04 | 2,17 |
| Snai1 | 10478884 | NM_011427 | 1,46E-03 | 2,17 |
| Ttc39b | 10514128 | NM_027238 | 1,31E-02 | 2,17 |
| 9930111J21Rik2 | 10385526 | NM_173434 | 2,28E-03 | 2,18 |
| Adam8 | 10568873 | NM_007403 | 4,09E-02 | 2,18 |
| Adap2 | 10379389 | NM_172133 | 2,32E-03 | 2,18 |
| Bmp2 | 10476395 | NM_007553 | 1,26E-02 | 2,18 |
| Cd209b | 10576816 | NM_026972 | 4,07E-02 | 2,18 |
| Cpne8 | 10431637 | NM_025815 | 7,11E-04 | 2,18 |
| Cxx1c | 10599654 | NM_028375 | 6,91E-03 | 2,18 |
| D8Ertd82e | 10571344 | NM_172911 | 6,95E-03 | 2,18 |
| Dusp6 | 10366043 | NM_026268 | 4,19E-03 | 2,18 |
| Gm10673 | 10501046 | ENSMUST00000098753 | 3,79E-02 | 2,18 |
| Gm13251 | 10518346 | NM_001085522 | 5,71E-03 | 2,18 |
| Gna14 | 10461856 | NM_008137 | 2,53E-02 | 2,18 |
| Gpr153 | 10510700 | NM_178406 | 2,19E-02 | 2,18 |
| Heph | 10600857 | NM_010417 | 1,45E-03 | 2,18 |
| Mir218-2 | 10375214 | NR_029799 | 9,76E-03 | 2,18 |
| Pde2a | 10555510 | NM_001143848 | 7,74E-03 | 2,18 |
| Ptpn13 | 10523595 | NM_011204 | 1,76E-03 | 2,18 |
| Ptpru | 10516789 | NM_001083119 | 8,49E-04 | 2,18 |
| Acy3 | 10460263 | NM_027857 | 6,24E-03 | 2,19 |
| Bmp4 | 10419261 | NM_007554 | 5,92E-04 | 2,19 |
| Cav1 | 10536499 | NM_007616 | 3,45E-03 | 2,19 |
| Cd200 | 10439651 | NM_010818 | 4,89E-03 | 2,19 |
| Col4a6 | 10607012 | NM_053185 | 3,39E-04 | 2,19 |
| Ift57 | 10436169 | NM_028680 | 4,85E-03 | 2,19 |
| Igfbp7 | 10530841 | NM_001159518 | 6,83E-04 | 2,19 |
| Lilrb3 | 10559446 | NM_011095 | 1,42E-02 | 2,19 |
| Megf9 | 10513869 | NM_172694 | 4,24E-03 | 2,19 |
| Nuak1 | 10371379 | NM_001004363 | 7,28E-03 | 2,19 |
| Pira11 | 10559454 | NM_011088 | 1,11E-02 | 2,19 |
| Plac1 | 10604616 | NM_019538 | 7,75E-03 | 2,19 |
| Prkca | 10392388 | NM_011101 | 6,18E-04 | 2,19 |
| Rem1 | 10477140 | NM_009047 | 1,96E-03 | 2,19 |
| Slco3a1 | 10564631 | NM_023908 | 3,36E-03 | 2,19 |
| Sparcl1 | 10531931 | NM_010097 | 1,06E-03 | 2,19 |
| Tbxas1 | 10537410 | NM_011539 | 3,21E-02 | 2,19 |
| Ust | 10367734 | NM_177387 | 3,93E-02 | 2,19 |
| Abca8b | 10392484 | NM_013851 | 1,82E-02 | 2,2 |
| Abcb1a | 10519527 | NM_011076 | 9,72E-03 | 2,2 |
| C4a | 10450280 | NM_011413 | 6,98E-03 | 2,2 |
| Cmah | 10404132 | NM_001111110 | 4,00E-02 | 2,2 |
| Fam171a1 | 10468916 | NM_001081161 | 2,29E-03 | 2,2 |
| Gpc2 | 10535021 | NM_172412 | 5,51E-03 | 2,2 |
| Kmo | 10352000 | NM_133809 | 7,82E-04 | 2,2 |
| Mir708 | 10555009 | NR_030489 | 1,00E-02 | 2,2 |
| Mrc2 | 10381898 | NM_008626 | 5,65E-03 | 2,2 |
| Mtap9 | 10492720 | NM_001081230 | 5,39E-03 | 2,2 |
| Myo1b | 10354432 | NM_001161817 | 1,47E-03 | 2,2 |
| Robo1 | 10436519 | NM_019413 | 5,26E-04 | 2,2 |
| Slc6a15 | 10366163 | NM_175328 | 1,15E-03 | 2,2 |
| Slfn5 | 10379615 | NM_183201 | 2,74E-03 | 2,2 |
| Cbr2 | 10393936 | NM_007621 | 5,96E-03 | 2,21 |
| Chst12 | 10527009 | NM_021528 | 1,08E-02 | 2,21 |
| Csmd1 | 10577240 | NM_053171 | 1,05E-03 | 2,21 |
| Cyp4b1 | 10515201 | NM_007823 | 6,04E-03 | 2,21 |
| Cysltr1 | 10606355 | NM_021476 | 3,94E-03 | 2,21 |
| Efha2 | 10571384 | NM_030110 | 9,04E-04 | 2,21 |
| Krt7 | 10427052 | NM_033073 | 2,62E-03 | 2,21 |
| Meox2 | 10395409 | NM_008584 | 1,28E-02 | 2,21 |
| Mrgprb2 | 10563760 | NM_175531 | 1,84E-02 | 2,21 |
| Msr1 | 10578264 | NM_031195 | 1,49E-02 | 2,21 |
| Pir | 10603087 | NM_027153 | 1,08E-02 | 2,21 |
| Plekhh2 | 10447190 | NM_177606 | 4,97E-04 | 2,21 |
| Pqlc3 | 10399540 | NM_172574 | 5,20E-03 | 2,21 |
| Sulf2 | 10489759 | NM_028072 | 6,86E-03 | 2,21 |
| Tacstd2 | 10545168 | NM_020047 | 3,13E-03 | 2,21 |
| Tcf4 | 10456522 | NM_013685 | 1,11E-03 | 2,21 |
| Tuba1a | 10432404 | NM_011653 | 3,57E-02 | 2,21 |
| Zfp608 | 10458940 | NM_175751 | 1,63E-03 | 2,21 |
| 1110051M20Rik | 10485013 | BC085239 | 1,87E-03 | 2,22 |
| Adcy4 | 10420171 | NM_080435 | 1,76E-03 | 2,22 |
| Adcy5 | 10435388 | NM_001012765 | 2,55E-02 | 2,22 |
| Axl | 10561104 | NM_009465 | 4,33E-03 | 2,22 |
| Efemp2 | 10460603 | NM_021474 | 4,37E-03 | 2,22 |
| Gm9930 | 10367770 | ENSMUST00000066742 | 2,01E-02 | 2,22 |
| Jag1 | 10488060 | NM_013822 | 1,42E-03 | 2,22 |
| Rasgrf2 | 10410995 | NM_009027 | 4,06E-03 | 2,22 |
| 1300014I06Rik | 10408629 | NM_025831 | 4,77E-02 | 2,23 |
| Abcc1 | 10433735 | NM_008576 | 3,07E-03 | 2,23 |
| Adcy7 | 10573747 | NM_007406 | 2,30E-03 | 2,23 |
| Arhgap6 | 10603182 | NM_009707 | 3,53E-03 | 2,23 |
| Bai2 | 10508500 | NM_173071 | 4,07E-03 | 2,23 |
| Cnrip1 | 10374406 | NM_029861 | 5,31E-03 | 2,23 |
| Gstz1 | 10397507 | NM_010363 | 2,45E-02 | 2,23 |
| Plekhg1 | 10367673 | NM_001033253 | 1,17E-03 | 2,23 |
| Tox3 | 10580522 | NM_172913 | 1,01E-02 | 2,23 |
| Btc | 10531274 | NM_007568 | 1,54E-03 | 2,24 |
| Cbr3 | 10436978 | NM_173047 | 2,21E-02 | 2,24 |
| Ccdc69 | 10385995 | NM_177471 | 1,09E-02 | 2,24 |
| Crim1 | 10446986 | NM_015800 | 4,06E-04 | 2,24 |
| Esrrb | 10397440 | NM_001159500 | 6,94E-03 | 2,24 |
| Fam102b | 10501456 | NM_001163567 | 6,83E-03 | 2,24 |
| Fkbp10 | 10381122 | NM_010221 | 8,82E-03 | 2,24 |
| Igsf9 | 10351801 | NM_033608 | 1,01E-03 | 2,24 |
| Lgals3bp | 10393573 | NM_011150 | 4,19E-03 | 2,24 |
| Man2a2 | 10564909 | NM_172903 | 6,28E-03 | 2,24 |
| Mir30e | 10515981 | NR_029602 | 3,47E-03 | 2,24 |
| Slc2a13 | 10431711 | NM_001033633 | 5,67E-03 | 2,24 |
| Twist1 | 10395320 | NM_011658 | 2,29E-02 | 2,24 |
| Adamts7 | 10587748 | NM_001003911 | 3,22E-03 | 2,25 |
| Adcyap1r1 | 10538482 | NM_007407 | 1,20E-02 | 2,25 |
| Ank | 10423363 | NM_020332 | 4,65E-02 | 2,25 |
| Cd300lg | 10381514 | NM_001160711 | 2,69E-03 | 2,25 |
| Dbn1 | 10409464 | NM_001177371 | 2,96E-03 | 2,25 |
| Ddx60 | 10571984 | NM_001081215 | 3,66E-02 | 2,25 |
| Hmcn1 | 10358601 | NM_001024720 | 9,58E-04 | 2,25 |
| Layn | 10593449 | NM_001033534 | 1,21E-03 | 2,25 |
| Mir145 | 10459225 | NR_029557 | 3,29E-02 | 2,25 |
| Ms4a14 | 10466190 | ENSMUST00000067600 | 1,69E-02 | 2,25 |
| Nrarp | 10469936 | NM_025980 | 1,90E-02 | 2,25 |
| Rnf24 | 10487879 | NM_178607 | 7,91E-03 | 2,25 |
| Cldn10a | 10417027 | NM_021386 | 1,36E-03 | 2,26 |
| Ephx3 | 10449807 | NM_001033163 | 2,67E-03 | 2,26 |
| Gja1 | 10363173 | NM_010288 | 3,97E-03 | 2,26 |
| Hectd2 | 10462702 | NM_001163471 | 4,92E-04 | 2,26 |
| Mocs2 | 10407307 | NM_013826 | 9,13E-04 | 2,26 |
| Rasl12 | 10586390 | NM_001033158 | 2,09E-02 | 2,26 |
| Spire1 | 10459552 | NM_194355 | 5,90E-03 | 2,26 |
| Syde1 | 10370510 | NM_027875 | 9,72E-03 | 2,26 |
| Abcc4 | 10422280 | NM_001033336 | 3,94E-03 | 2,27 |
| Abcc5 | 10438478 | NM_013790 | 2,30E-03 | 2,27 |
| C5ar1 | 10560242 | NM_001173550 | 4,73E-02 | 2,27 |
| Dok6 | 10460127 | NM_001039173 | 1,94E-03 | 2,27 |
| Dopey1 | 10587558 | NM_177208 | 1,14E-03 | 2,27 |
| Hvcn1 | 10525365 | NM_001042489 | 2,63E-02 | 2,27 |
| Nxf7 | 10606792 | NM_130888 | 1,77E-03 | 2,27 |
| Plxdc2 | 10469457 | NM_026162 | 1,63E-03 | 2,27 |
| Apoe | 10560624 | NM_009696 | 2,29E-03 | 2,28 |
| Arhgap40 | 10478114 | NM_001145015 | 5,41E-03 | 2,28 |
| Bgn | 10600169 | NM_007542 | 1,75E-03 | 2,28 |
| Car8 | 10511429 | NM_007592 | 1,05E-03 | 2,28 |
| Cd248 | 10460541 | NM_054042 | 2,98E-02 | 2,28 |
| Cfp | 10603860 | NM_008823 | 7,06E-03 | 2,28 |
| Col5a2 | 10354309 | NM_007737 | 1,69E-02 | 2,28 |
| Dusp1 | 10449284 | NM_013642 | 1,05E-03 | 2,28 |
| Emilin2 | 10452648 | NM_145158 | 3,92E-02 | 2,28 |
| Gm16503 | 10510239 | ENSMUST00000104964 | 1,33E-02 | 2,28 |
| Gramd4 | 10426042 | NM_172611 | 3,89E-03 | 2,28 |
| Hoxc10 | 10427280 | NM_010462 | 3,06E-02 | 2,28 |
| Palm | 10364502 | NM_023128 | 1,44E-02 | 2,28 |
| Plvap | 10579525 | NM_032398 | 1,52E-02 | 2,28 |
| Ralgapa2 | 10488322 | NM_001033348 | 6,46E-03 | 2,28 |
| Cd37 | 10563178 | NM_007645 | 2,05E-02 | 2,29 |
| Cyp2j9 | 10514520 | NM_028979 | 1,48E-03 | 2,29 |
| Dpysl3 | 10458663 | NM_009468 | 8,48E-04 | 2,29 |
| Itga11 | 10586079 | NM_176922 | 2,40E-02 | 2,29 |
| Mia1 | 10561187 | NM_019394 | 1,91E-03 | 2,29 |
| Mpdz | 10514000 | NM_010820 | 9,35E-04 | 2,29 |
| P2ry6 | 10565958 | NM_183168 | 3,01E-02 | 2,29 |
| Plac9 | 10418193 | NM_207229 | 1,60E-02 | 2,29 |
| Prrg3 | 10600044 | NM_001081135 | 4,51E-02 | 2,29 |
| Shc2 | 10370587 | NM_001024539 | 1,10E-03 | 2,29 |
| Sp100 | 10347948 | NM_013673 | 5,60E-04 | 2,29 |
| Tlr8 | 10607868 | NM_133212 | 3,55E-02 | 2,29 |
| Tmprss6 | 10430324 | NM_027902 | 1,40E-02 | 2,29 |
| Wnk4 | 10381311 | NM_175638 | 7,00E-03 | 2,29 |
| Csf2rb | 10425066 | NM_007780 | 3,52E-03 | 2,3 |
| Ctsf | 10460468 | NM_019861 | 1,57E-02 | 2,3 |
| Elf4 | 10604393 | NM_019680 | 1,17E-02 | 2,3 |
| Slc6a6 | 10540122 | NM_009320 | 6,02E-03 | 2,3 |
| Armcx6 | 10606730 | NM_001007578 | 1,20E-02 | 2,31 |
| Gm16485 | 10583195 | ENSMUST00000104915 | 1,87E-03 | 2,31 |
| Kif5c | 10471994 | NM_008449 | 1,40E-03 | 2,31 |
| Tfpi | 10484389 | NM_011576 | 2,15E-02 | 2,31 |
| Trim29 | 10584604 | NM_023655 | 2,30E-03 | 2,31 |
| Vim | 10469322 | NM_011701 | 1,23E-03 | 2,31 |
| Akap2 | 10505143 | ENSMUST00000107600 | 1,36E-03 | 2,32 |
| Col6a1 | 10370210 | NM_009933 | 1,56E-02 | 2,32 |
| Cpe | 10578904 | NM_013494 | 2,69E-03 | 2,32 |
| Emr1 | 10446282 | NM_010130 | 4,15E-02 | 2,32 |
| Hspb8 | 10533050 | NM_030704 | 4,28E-02 | 2,32 |
| Ifitm3 | 10569017 | NM_025378 | 1,25E-03 | 2,32 |
| Itgb2 | 10364262 | NM_008404 | 2,80E-02 | 2,32 |
| Mmp16 | 10503448 | NM_019724 | 1,09E-03 | 2,32 |
| Rxrg | 10351430 | NM_009107 | 1,82E-02 | 2,32 |
| Slc16a10 | 10368720 | NM_001114332 | 9,05E-03 | 2,32 |
| Arhgef10 | 10570483 | NM_172751 | 1,48E-03 | 2,33 |
| Ccdc141 | 10484205 | ENSMUST00000049544 | 1,31E-02 | 2,33 |
| Cd34 | 10352905 | NM_001111059 | 9,85E-03 | 2,33 |
| Csrp2 | 10366293 | NM_007792 | 2,83E-03 | 2,33 |
| Hip1 | 10534456 | NM_146001 | 3,53E-03 | 2,33 |
| Id3 | 10509163 | NM_008321 | 1,76E-02 | 2,33 |
| Lamc3 | 10471216 | NM_011836 | 4,90E-03 | 2,33 |
| Oas2 | 10533198 | NM_145227 | 4,78E-02 | 2,33 |
| Palm2 | 10505120 | NM_172868 | 7,27E-04 | 2,33 |
| Pdpn | 10518147 | NM_010329 | 1,60E-03 | 2,33 |
| Tbx15 | 10494672 | NM_009323 | 7,84E-03 | 2,33 |
| Tyrobp | 10551883 | NM_011662 | 3,86E-03 | 2,33 |
| Xlr4b | 10600131 | NM_021365 | 1,81E-02 | 2,33 |
| Dlc1 | 10578241 | HM008381 | 2,40E-03 | 2,34 |
| Fam149a | 10578477 | NM_153535 | 1,23E-02 | 2,34 |
| Mtmr11 | 10494351 | NM_181409 | 1,35E-03 | 2,34 |
| Npy1r | 10572070 | NM_010934 | 7,35E-03 | 2,34 |
| Pcdhb6 | 10455069 | NM_053131 | 5,80E-03 | 2,34 |
| Reck | 10504424 | NM_016678 | 1,16E-03 | 2,34 |
| Spry4 | 10458555 | NM_011898 | 8,76E-03 | 2,34 |
| Trhde | 10372421 | NM_146241 | 8,54E-03 | 2,34 |
| Xlr4c | 10605034 | NM_183094 | 1,78E-02 | 2,34 |
| Aldh1a1 | 10461979 | NM_013467 | 5,31E-03 | 2,35 |
| BC013712 | 10516966 | NM_001033308 | 8,63E-03 | 2,35 |
| Cc2d2a | 10521626 | NM_172274 | 3,09E-04 | 2,35 |
| Ebf3 | 10568735 | NM_001113415 | 8,78E-03 | 2,35 |
| Ecm2 | 10405033 | NM_001012324 | 1,44E-03 | 2,35 |
| Eln | 10534343 | NM_007925 | 2,57E-03 | 2,35 |
| Ift81 | 10533504 | NM_009879 | 6,77E-03 | 2,35 |
| Opn3 | 10360454 | NM_010098 | 1,44E-02 | 2,35 |
| Slc35f3 | 10576586 | NM_175434 | 1,53E-02 | 2,35 |
| 4430402I18Rik | 10466903 | NM_198651 | 2,75E-02 | 2,36 |
| Bicc1 | 10369844 | NM_031397 | 6,04E-03 | 2,36 |
| Ccdc3 | 10469066 | NM_028804 | 4,31E-02 | 2,36 |
| Fhl1 | 10599736 | NM_001077361 | 4,23E-02 | 2,36 |
| Il34 | 10581664 | NM_029646 | 5,53E-03 | 2,36 |
| Laptm5 | 10508663 | NM_010686 | 5,33E-03 | 2,36 |
| Npr1 | 10499811 | NM_008727 | 7,82E-04 | 2,36 |
| Nrp1 | 10576639 | NM_008737 | 1,54E-03 | 2,36 |
| Prkd1 | 10400170 | NM_008858 | 6,26E-03 | 2,36 |
| Prrt2 | 10568135 | NM_001102563 | 3,49E-04 | 2,36 |
| Sult1a1 | 10568001 | NM_133670 | 1,01E-02 | 2,36 |
| Bmp1 | 10421361 | NR_033241 | 2,78E-03 | 2,37 |
| Calcrl | 10484371 | NM_018782 | 2,42E-02 | 2,37 |
| Dhrs3 | 10510129 | NM_011303 | 1,23E-03 | 2,37 |
| Fbln5 | 10402211 | NM_011812 | 4,22E-03 | 2,37 |
| Fibin | 10485711 | NM_026271 | 2,21E-03 | 2,37 |
| Hoxc5 | 10427297 | NM_175730 | 5,31E-03 | 2,37 |
| Pappa | 10505489 | NM_021362 | 5,67E-03 | 2,37 |
| 4930578C19Rik | 10603796 | BC118515 | 5,35E-03 | 2,38 |
| Adcy3 | 10394258 | NM_001159537 | 1,73E-02 | 2,38 |
| Cxcl15 | 10523145 | NM_011339 | 2,04E-03 | 2,38 |
| Emp1 | 10542355 | NM_010128 | 1,48E-02 | 2,38 |
| Enpp1 | 10368289 | NM_008813 | 4,07E-04 | 2,38 |
| Gucy1b3 | 10498935 | NM_017469 | 1,56E-03 | 2,38 |
| Man1a | 10369154 | NM_008548 | 1,78E-03 | 2,38 |
| Plcb1 | 10476401 | NM_019677 | 9,09E-04 | 2,38 |
| Pof1b | 10606495 | NM_181579 | 5,60E-04 | 2,38 |
| Ptn | 10543959 | NM_008973 | 7,74E-03 | 2,38 |
| Serpina3h | 10398052 | NR_033450 | 1,19E-03 | 2,38 |
| Snx32 | 10465089 | NM_001024560 | 5,45E-04 | 2,38 |
| Soat1 | 10359161 | NM_009230 | 8,24E-04 | 2,38 |
| 4632428N05Rik | 10363445 | NM_028732 | 1,94E-02 | 2,39 |
| Abca9 | 10392560 | NM_147220 | 1,10E-02 | 2,39 |
| Aifm2 | 10363528 | NM_001039194 | 1,24E-02 | 2,39 |
| Chst1 | 10474045 | NM_023850 | 4,16E-02 | 2,39 |
| Fam198b | 10492682 | NM_133187 | 8,94E-04 | 2,39 |
| Ly6c2 | 10429573 | NM_001099217 | 2,44E-03 | 2,39 |
| Naaa | 10531370 | NM_025972 | 6,87E-03 | 2,39 |
| Scara3 | 10420891 | NM_172604 | 1,12E-02 | 2,39 |
| Snai2 | 10433776 | NM_011415 | 1,26E-03 | 2,39 |
| Tgfbi | 10405587 | NM_009369 | 8,01E-03 | 2,39 |
| Csf1r | 10456071 | NM_001037859 | 1,36E-02 | 2,4 |
| Ifi203 | 10360391 | NM_001045481 | 1,75E-03 | 2,4 |
| Lama4 | 10362538 | NM_010681 | 3,05E-03 | 2,4 |
| Pla2r1 | 10482968 | NM_008867 | 7,96E-04 | 2,4 |
| 4921506M07Rik | 10395869 | BC100489 | 1,67E-03 | 2,41 |
| Gpr137b | 10407803 | NM_031999 | 1,11E-03 | 2,41 |
| Lims2 | 10454514 | NM_144862 | 1,12E-02 | 2,41 |
| Oscp1 | 10508099 | NM_172701 | 5,50E-03 | 2,41 |
| Phlda3 | 10350146 | NM_013750 | 3,01E-02 | 2,41 |
| Zeb1 | 10453678 | NM_011546 | 8,00E-04 | 2,41 |
| Basp1 | 10427895 | NM_027395 | 6,23E-03 | 2,42 |
| Ccl11 | 10379524 | NM_011330 | 1,39E-02 | 2,42 |
| Elovl4 | 10595392 | NM_148941 | 3,26E-03 | 2,42 |
| Gm13139 | 10510215 | NM_001083918 | 6,36E-03 | 2,42 |
| Igf1r | 10554094 | NM_010513 | 9,04E-04 | 2,42 |
| Il1r1 | 10345762 | NM_008362 | 1,87E-03 | 2,42 |
| Pon1 | 10542983 | NM_011134 | 1,89E-02 | 2,42 |
| Thbd | 10488378 | NM_009378 | 6,47E-03 | 2,42 |
| Ar | 10600901 | NM_013476 | 9,04E-04 | 2,43 |
| BC055004 | 10526783 | NM_001013773 | 1,66E-02 | 2,43 |
| Ecm1 | 10500204 | NM_007899 | 7,28E-03 | 2,43 |
| Hpca | 10516544 | NM_010471 | 1,54E-02 | 2,43 |
| Mcam | 10584674 | NM_023061 | 2,86E-03 | 2,43 |
| Sh3pxd2a | 10468309 | NM_008018 | 1,60E-02 | 2,43 |
| Sorbs3 | 10421269 | NM_011366 | 5,65E-03 | 2,43 |
| Uggt2 | 10422348 | NM_001081252 | 4,98E-04 | 2,43 |
| Zfp385a | 10433104 | NM_013866 | 9,04E-03 | 2,43 |
| Akr1b8 | 10537146 | NM_008012 | 3,25E-02 | 2,44 |
| Bmp7 | 10490129 | NM_007557 | 4,36E-03 | 2,44 |
| Cdh2 | 10457644 | NM_007664 | 1,24E-02 | 2,44 |
| Fmo2 | 10359582 | NM_018881 | 1,70E-03 | 2,44 |
| Htr2a | 10416406 | NM_172812 | 5,03E-04 | 2,44 |
| Itgb3 | 10381809 | NM_016780 | 4,71E-03 | 2,44 |
| Lama2 | 10368409 | NM_008481 | 8,59E-03 | 2,44 |
| Tmem184b | 10430510 | NM_172608 | 7,00E-03 | 2,44 |
| Zfp354c | 10385635 | NM_013922 | 6,66E-04 | 2,44 |
| Ankrd44 | 10354677 | NM_001081433 | 7,96E-04 | 2,45 |
| Arhgef2 | 10493267 | NM_008487 | 2,14E-03 | 2,45 |
| Asrgl1 | 10465844 | NM_025610 | 9,23E-04 | 2,45 |
| Col1a1 | 10380419 | NM_007742 | 3,13E-02 | 2,45 |
| Gabbr1 | 10445078 | NM_019439 | 6,83E-04 | 2,45 |
| Ifit1 | 10462623 | NM_008331 | 4,22E-02 | 2,45 |
| Lass6 | 10472501 | NM_172856 | 8,48E-04 | 2,45 |
| Lyz2 | 10372648 | NM_017372 | 7,42E-03 | 2,45 |
| Mtap6 | 10555197 | NM_010837 | 1,36E-02 | 2,45 |
| Prss12 | 10495854 | NM_008939 | 1,48E-03 | 2,45 |
| Rgs18 | 10358421 | NM_022881 | 1,55E-02 | 2,45 |
| Six1 | 10400967 | NM_009189 | 5,66E-03 | 2,45 |
| Egr1 | 10454782 | NM_007913 | 1,90E-02 | 2,46 |
| Epb4.1l2 | 10362245 | NM_013511 | 2,04E-03 | 2,46 |
| Fam40b | 10536949 | NM_177204 | 2,15E-02 | 2,46 |
| Galntl4 | 10566993 | NM_173739 | 8,86E-04 | 2,46 |
| Robo2 | 10440344 | NM_175549 | 9,09E-04 | 2,46 |
| Tlr7 | 10607870 | NM_133211 | 1,18E-02 | 2,46 |
| Tnfrsf1b | 10518300 | NM_011610 | 9,04E-03 | 2,46 |
| Ampd3 | 10556302 | NM_009667 | 1,20E-02 | 2,47 |
| Gm5431 | 10385504 | NM_001024230 | 8,85E-03 | 2,47 |
| Irak1bp1 | 10587495 | NM_022986 | 5,02E-03 | 2,47 |
| Loxl3 | 10539263 | NM_013586 | 1,11E-03 | 2,47 |
| Mtmr7 | 10578300 | NM_001040699 | 5,31E-03 | 2,47 |
| Scn7a | 10483353 | NM_009135 | 1,52E-03 | 2,47 |
| Tspan12 | 10543306 | NM_173007 | 2,44E-03 | 2,47 |
| 1110059M19Rik | 10599422 | NM_026841 | 3,27E-02 | 2,48 |
| 2610019F03Rik | 10577226 | NM_173744 | 3,09E-02 | 2,48 |
| Casc4 | 10475378 | NM_177054 | 6,30E-04 | 2,48 |
| Galntl2 | 10413813 | NM_030166 | 1,30E-03 | 2,48 |
| Gm10035 | 10426894 | NM_001081471 | 2,78E-02 | 2,48 |
| Hspb1 | 10408928 | NM_013560 | 5,86E-03 | 2,48 |
| Itm2a | 10606369 | NM_008409 | 3,09E-02 | 2,48 |
| Rhoj | 10396476 | NM_023275 | 1,01E-03 | 2,48 |
| 4930420K17Rik | 10519607 | BC147127 | 5,90E-03 | 2,49 |
| Adora1 | 10357878 | NM_001008533 | 3,66E-02 | 2,49 |
| Cdc42ep2 | 10465278 | NM_026772 | 1,04E-02 | 2,49 |
| F630028O10Rik | 10600852 | NR_030718 | 5,59E-03 | 2,49 |
| Fam83a | 10424245 | NM_173862 | 1,25E-03 | 2,49 |
| Gpr124 | 10571142 | NM_054044 | 3,68E-03 | 2,49 |
| Gpr64 | 10602896 | NM_178712 | 6,80E-04 | 2,49 |
| Has2 | 10428707 | NM_008216 | 2,67E-03 | 2,49 |
| Plxnc1 | 10372028 | NM_018797 | 1,62E-03 | 2,49 |
| Prr5l | 10485378 | NM_001083810 | 1,18E-02 | 2,49 |
| Sema4a | 10499378 | NM_013658 | 6,80E-03 | 2,49 |
| Ccnd2 | 10548105 | NM_009829 | 7,50E-03 | 2,5 |
| Dennd2a | 10544199 | NM_172477 | 4,67E-03 | 2,5 |
| Islr | 10594044 | NM_012043 | 4,04E-02 | 2,5 |
| Ly6c1 | 10429568 | NM_010741 | 3,53E-03 | 2,5 |
| Pcdh7 | 10521972 | NM_018764 | 8,83E-03 | 2,5 |
| Pde1a | 10484283 | NM_016744 | 1,95E-03 | 2,5 |
| Pdk3 | 10605711 | NM_145630 | 3,40E-03 | 2,5 |
| Prkce | 10447294 | NM_011104 | 4,53E-02 | 2,5 |
| Ptgfr | 10502805 | NM_008966 | 3,78E-03 | 2,5 |
| Ptrf | 10391332 | NM_008986 | 1,66E-02 | 2,5 |
| Usp11 | 10598933 | NM_145628 | 4,06E-04 | 2,5 |
| Vwa3a | 10556962 | NM_177697 | 3,64E-03 | 2,5 |
| Ablim1 | 10468691 | NM_178688 | 1,15E-03 | 2,51 |
| Ankrd5 | 10476497 | NM_175667 | 1,36E-02 | 2,51 |
| Cdkn2c | 10515090 | NM_007671 | 2,14E-02 | 2,51 |
| Fcer1g | 10360070 | NM_010185 | 1,60E-02 | 2,51 |
| H2-M9 | 10450784 | NM_008205 | 6,23E-03 | 2,51 |
| Pf4 | 10523134 | NM_019932 | 4,14E-02 | 2,51 |
| Pira1 | 10559467 | NM_011087 | 1,53E-02 | 2,51 |
| Runx2 | 10451061 | NM_001146038 | 8,31E-04 | 2,51 |
| Celf2 | 10479950 | NM_001110231 | 1,47E-03 | 2,52 |
| Col3a1 | 10346015 | NM_009930 | 1,18E-02 | 2,52 |
| Crispld2 | 10575976 | NM_030209 | 5,75E-03 | 2,52 |
| Ifi202b | 10360398 | NM_008327 | 2,00E-02 | 2,52 |
| Lgals9 | 10388902 | NM_010708 | 4,21E-03 | 2,52 |
| 2010110P09Rik | 10557165 | NM_027363 | 3,61E-03 | 2,53 |
| Ang | 10414537 | NM_001161731 | 4,17E-03 | 2,53 |
| Aspa | 10388254 | NM_023113 | 3,72E-02 | 2,53 |
| Atp11a | 10570201 | NM_015804 | 4,18E-04 | 2,53 |
| Il7r | 10427628 | NM_008372 | 4,21E-03 | 2,53 |
| Jam2 | 10436666 | NM_023844 | 1,79E-03 | 2,53 |
| Mr1 | 10358982 | NM_008209 | 7,82E-04 | 2,53 |
| Notch1 | 10481056 | NM_008714 | 4,53E-03 | 2,53 |
| Plod1 | 10518408 | NM_011122 | 8,02E-03 | 2,53 |
| Rgl1 | 10358733 | NM_016846 | 1,92E-03 | 2,53 |
| Idh1 | 10355214 | NM_010497 | 9,86E-03 | 2,54 |
| Lgals1 | 10425161 | NM_008495 | 2,02E-02 | 2,54 |
| Lipm | 10462575 | NM_023903 | 7,35E-03 | 2,54 |
| Mogat1 | 10347741 | NM_026713 | 4,99E-02 | 2,54 |
| Rassf2 | 10487894 | NM_175445 | 1,02E-02 | 2,54 |
| Tox | 10511416 | NM_145711 | 3,49E-04 | 2,54 |
| Ahnak2 | 10402787 | ENSMUST00000101009 | 1,07E-02 | 2,55 |
| Akr1c14 | 10403291 | NM_134072 | 2,28E-02 | 2,55 |
| Dennd5b | 10549506 | NM_177192 | 2,77E-04 | 2,55 |
| Oit1 | 10417568 | NM_146050 | 2,74E-03 | 2,55 |
| Pparg | 10540897 | NM_001127330 | 4,37E-03 | 2,55 |
| Rex2 | 10510201 | NM_009051 | 2,60E-02 | 2,55 |
| Slc16a7 | 10372988 | NM_011391 | 7,81E-03 | 2,55 |
| 9030425E11Rik | 10584561 | NM_133733 | 3,09E-03 | 2,56 |
| Clec12a | 10542164 | NM_177686 | 2,98E-02 | 2,56 |
| Gfra2 | 10416340 | NM_008115 | 5,31E-03 | 2,56 |
| Hoxb3 | 10380654 | NM_001079869 | 1,07E-03 | 2,56 |
| Mtap7d3 | 10604694 | NM_177293 | 2,74E-03 | 2,56 |
| Pde7b | 10368175 | NM_013875 | 2,77E-04 | 2,56 |
| Pfkfb1 | 10602385 | NM_008824 | 1,00E-02 | 2,56 |
| Pik3cg | 10399924 | NM_020272 | 1,04E-03 | 2,56 |
| Ehd2 | 10560190 | NM_153068 | 7,82E-03 | 2,57 |
| Htr1d | 10509238 | NM_008309 | 1,31E-03 | 2,57 |
| Mir26a-1 | 10590071 | NR_029742 | 1,79E-03 | 2,57 |
| Vcan | 10410931 | NM_001081249 | 5,38E-03 | 2,57 |
| Nox4 | 10554752 | NM_015760 | 8,57E-04 | 2,58 |
| Sema5a | 10423520 | NM_009154 | 2,83E-03 | 2,58 |
| Abcg1 | 10443730 | NM_009593 | 2,05E-03 | 2,59 |
| BC034902 | 10476493 | BC034902 | 8,60E-03 | 2,59 |
| Edil3 | 10406504 | NM_001037987 | 9,96E-03 | 2,59 |
| Gm9992 | 10447634 | NM_001142539 | 4,72E-03 | 2,59 |
| Igsf6 | 10567580 | NM_030691 | 1,18E-03 | 2,59 |
| Nav2 | 10553354 | NM_175272 | 8,37E-04 | 2,59 |
| Timp3 | 10365482 | NM_011595 | 7,82E-04 | 2,59 |
| Ugt8a | 10501963 | NM_011674 | 6,12E-04 | 2,59 |
| Xlr3b | 10600122 | NM_001081643 | 4,74E-03 | 2,59 |
| 4930588N13Rik | 10527658 | BC120819 | 4,83E-04 | 2,6 |
| Col1a2 | 10536220 | NM_007743 | 2,44E-02 | 2,6 |
| Dclk1 | 10492136 | NM_019978 | 2,77E-04 | 2,6 |
| F3 | 10495675 | NM_010171 | 4,90E-03 | 2,6 |
| Fgf14 | 10422585 | NM_207667 | 1,84E-02 | 2,6 |
| Gm12824 | 10507099 | NM_001085549 | 2,51E-02 | 2,6 |
| Mdk | 10485070 | NM_010784 | 3,99E-03 | 2,6 |
| Ms4a4a | 10461587 | XM_889011 | 3,48E-03 | 2,6 |
| Osr1 | 10394534 | NM_011859 | 7,83E-04 | 2,6 |
| Plscr4 | 10587818 | NM_178711 | 1,48E-03 | 2,6 |
| Rbms3 | 10597531 | NM_001172123 | 7,82E-04 | 2,6 |
| Samd5 | 10367772 | NM_177271 | 5,39E-03 | 2,6 |
| Il2rg | 10606016 | NM_013563 | 5,47E-04 | 2,61 |
| Kirrel | 10499168 | NM_001170985 | 3,53E-03 | 2,61 |
| Mitf | 10540248 | NM_001113198 | 1,63E-03 | 2,61 |
| Nqo1 | 10581538 | NM_008706 | 2,99E-03 | 2,61 |
| Pcdh18 | 10498018 | NM_130448 | 5,51E-04 | 2,61 |
| Sspn | 10542740 | NM_010656 | 1,89E-02 | 2,61 |
| Tmem43 | 10540105 | NM_028766 | 3,17E-03 | 2,61 |
| Bcam | 10560655 | NM_020486 | 7,50E-03 | 2,62 |
| Fgfrl1 | 10524052 | NM_054071 | 8,22E-03 | 2,62 |
| Gltp | 10532857 | NM_019821 | 1,01E-02 | 2,62 |
| Gm10855 | 10479971 | ENSMUST00000100426 | 3,76E-03 | 2,62 |
| Hexa | 10585874 | NM_010421 | 1,56E-02 | 2,62 |
| Palmd | 10501734 | NM_023245 | 2,21E-03 | 2,62 |
| Pfkfb3 | 10480035 | NM_001177753 | 4,08E-02 | 2,62 |
| Rgs2 | 10358389 | NM_009061 | 3,64E-02 | 2,62 |
| Trim47 | 10393106 | NM_172570 | 1,09E-02 | 2,62 |
| Fads3 | 10461423 | NM_021890 | 1,81E-02 | 2,63 |
| Fst | 10412260 | NM_008046 | 5,16E-04 | 2,63 |
| Prdm5 | 10538811 | NM_027547 | 9,69E-04 | 2,63 |
| Taf9b | 10606315 | NM_001167988 | 1,32E-03 | 2,63 |
| Ugt1a9 | 10348354 | NM_201644 | 6,57E-03 | 2,63 |
| Fstl1 | 10435641 | NM_008047 | 1,10E-02 | 2,64 |
| Gm11711 | 10392825 | NM_001101657 | 2,84E-02 | 2,64 |
| Mir27b | 10405781 | NR_029531 | 1,43E-02 | 2,64 |
| Serpina1e | 10402409 | NM_009247 | 2,15E-02 | 2,64 |
| Slc9a9 | 10587854 | NM_177909 | 7,06E-03 | 2,64 |
| Sox8 | 10448967 | NM_011447 | 4,59E-04 | 2,64 |
| Tbc1d2b | 10595680 | NM_194334 | 2,32E-03 | 2,64 |
| 1200009I06Rik | 10398649 | NM_028807 | 5,35E-03 | 2,65 |
| Eps8 | 10548905 | NM_007945 | 3,49E-04 | 2,65 |
| Lass4 | 10569972 | NM_026058 | 2,36E-03 | 2,65 |
| Npr2 | 10504375 | NM_173788 | 1,15E-03 | 2,65 |
| Osmr | 10427471 | NM_011019 | 1,74E-03 | 2,65 |
| 2010007H06Rik | 10585282 | ENSMUST00000050829 | 1,02E-03 | 2,66 |
| Igfbp5 | 10355500 | NM_010518 | 1,65E-03 | 2,66 |
| Sipa1l1 | 10397002 | NM_001167983 | 4,18E-03 | 2,66 |
| Crip1 | 10399005 | NM_007763 | 1,34E-02 | 2,67 |
| Nr1h5 | 10500780 | NM_198658 | 2,69E-03 | 2,67 |
| Prr16 | 10455687 | NM_001081224 | 7,84E-03 | 2,67 |
| 2810055G20Rik | 10436590 | NR_015543 | 1,44E-03 | 2,68 |
| Bmper | 10583870 | NM_028472 | 1,71E-02 | 2,68 |
| Cygb | 10393364 | NM_030206 | 1,42E-02 | 2,68 |
| Darc | 10360344 | NM_010045 | 1,79E-03 | 2,68 |
| Epb4.1l3 | 10446553 | NM_013813 | 2,77E-04 | 2,68 |
| Gdpd1 | 10389590 | NM_025638 | 5,92E-04 | 2,68 |
| Gm10032 | 10416107 | ENSMUST00000071522 | 3,52E-02 | 2,68 |
| Gstm2 | 10501222 | NM_008183 | 2,27E-02 | 2,68 |
| Hey2 | 10368556 | NM_013904 | 2,30E-03 | 2,68 |
| Pcsk5 | 10466530 | NM_001163144 | 1,62E-03 | 2,68 |
| Sh2b2 | 10534585 | NM_018825 | 3,00E-02 | 2,68 |
| Tm4sf1 | 10498273 | NM_008536 | 7,11E-04 | 2,68 |
| Fbln1 | 10425945 | NM_010180 | 7,75E-03 | 2,69 |
| Mgst3 | 10359861 | NM_025569 | 1,11E-02 | 2,69 |
| Myo5a | 10587107 | NM_010864 | 1,40E-03 | 2,69 |
| Pcolce | 10534862 | NM_008788 | 1,06E-02 | 2,69 |
| St3gal6 | 10440099 | NM_018784 | 5,74E-04 | 2,69 |
| Anxa2 | 10586744 | NM_007585 | 6,68E-03 | 2,7 |
| Apobec1 | 10547621 | NM_031159 | 6,79E-04 | 2,7 |
| C1qc | 10517513 | NM_007574 | 1,19E-02 | 2,7 |
| Dsg1a | 10454113 | NM_010079 | 4,07E-04 | 2,7 |
| Ephx2 | 10420935 | NM_007940 | 1,96E-02 | 2,7 |
| F13a1 | 10408693 | NM_028784 | 4,15E-02 | 2,7 |
| Gdf10 | 10414025 | NM_145741 | 3,51E-03 | 2,7 |
| Lum | 10365983 | NM_008524 | 2,12E-02 | 2,7 |
| Slc19a1 | 10364239 | NM_031196 | 2,64E-02 | 2,7 |
| Aif1l | 10471247 | NM_145144 | 1,05E-03 | 2,71 |
| Egflam | 10427496 | NM_178748 | 1,86E-03 | 2,71 |
| Pgr | 10583179 | NM_008829 | 8,48E-04 | 2,71 |
| Sdpr | 10346164 | NM_138741 | 2,48E-03 | 2,71 |
| Selp | 10351206 | NM_011347 | 2,24E-03 | 2,71 |
| Siglech | 10553559 | NM_178706 | 6,29E-04 | 2,71 |
| Sik2 | 10593430 | NM_178710 | 4,77E-03 | 2,71 |
| Tenc1 | 10427095 | NM_153533 | 1,00E-02 | 2,71 |
| 2610203C20Rik | 10584595 | NR_015483 | 3,24E-03 | 2,72 |
| Abcc3 | 10389894 | NM_029600 | 4,28E-03 | 2,72 |
| Hepacam2 | 10542917 | NM_178899 | 6,61E-04 | 2,72 |
| Laptm4b | 10423593 | NM_033521 | 4,45E-03 | 2,72 |
| Nav1 | 10358091 | NM_173437 | 4,40E-03 | 2,72 |
| Adamts15 | 10591988 | NM_001024139 | 1,32E-03 | 2,73 |
| Cd302 | 10482920 | NM_025422 | 9,76E-03 | 2,74 |
| Ednra | 10579812 | NM_010332 | 6,60E-03 | 2,74 |
| Igfbp4 | 10381096 | NM_010517 | 1,26E-02 | 2,74 |
| Tpsb2 | 10442786 | NM_010781 | 8,51E-04 | 2,74 |
| Irf4 | 10404389 | NM_013674 | 4,72E-02 | 2,75 |
| Serpine2 | 10355984 | NM_009255 | 1,11E-03 | 2,75 |
| Ddr2 | 10359929 | NM_022563 | 8,80E-04 | 2,76 |
| Gas7 | 10377215 | NM_008088 | 7,47E-03 | 2,76 |
| Flrt2 | 10397633 | NM_201518 | 6,76E-03 | 2,77 |
| Gp49a | 10363070 | NM_008147 | 2,55E-02 | 2,77 |
| Cftr | 10536563 | NM_021050 | 8,38E-03 | 2,78 |
| Cryab | 10585214 | NM_009964 | 4,02E-03 | 2,78 |
| Ebf1 | 10375358 | ENSMUST00000081265 | 1,15E-03 | 2,78 |
| Mettl7a1 | 10426891 | NM_027334 | 2,38E-02 | 2,78 |
| Slc10a6 | 10531887 | NM_029415 | 6,47E-03 | 2,78 |
| Ttyh2 | 10382376 | NM_053273 | 8,02E-03 | 2,78 |
| Crabp1 | 10585438 | NM_013496 | 1,18E-02 | 2,79 |
| Agpat9 | 10523547 | NM_172715 | 4,76E-02 | 2,8 |
| Gas6 | 10577164 | NM_019521 | 3,64E-03 | 2,8 |
| Hoxb2 | 10380660 | NM_134032 | 1,05E-02 | 2,8 |
| Plcd1 | 10597575 | NM_019676 | 2,46E-03 | 2,8 |
| Serpinb8 | 10349174 | NM_011459 | 1,07E-02 | 2,8 |
| Gpc6 | 10416974 | NM_001079844 | 1,35E-03 | 2,81 |
| Igfbp6 | 10427125 | NM_008344 | 3,28E-02 | 2,81 |
| Lrrc39 | 10495562 | NM_175413 | 4,74E-03 | 2,81 |
| Mgl2 | 10377774 | NM_145137 | 3,60E-02 | 2,81 |
| Mgp | 10548879 | NM_008597 | 2,39E-03 | 2,81 |
| Mme | 10492355 | NM_008604 | 2,18E-03 | 2,81 |
| Papln | 10397112 | NM_130887 | 5,34E-04 | 2,81 |
| Unc93a | 10447904 | NM_199252 | 4,51E-03 | 2,81 |
| Ebf2 | 10416126 | NM_010095 | 2,99E-03 | 2,82 |
| Gria3 | 10599348 | NM_016886 | 2,96E-03 | 2,82 |
| Il4i1 | 10552743 | NM_010215 | 1,02E-03 | 2,82 |
| Nid1 | 10403584 | NM_010917 | 5,65E-03 | 2,82 |
| 4933409K07Rik | 10504172 | NR_033123 | 6,08E-04 | 2,83 |
| Aox1 | 10346374 | NM_009676 | 5,88E-03 | 2,83 |
| Calca | 10567095 | NM_007587 | 1,66E-02 | 2,83 |
| Cpne2 | 10574166 | NM_153507 | 2,64E-03 | 2,83 |
| Olfml1 | 10556076 | NM_172907 | 4,37E-02 | 2,83 |
| Rasa3 | 10577190 | NM_009025 | 3,84E-03 | 2,83 |
| Ucma | 10469058 | NM_001113558 | 1,10E-03 | 2,83 |
| Ace | 10381962 | NM_207624 | 6,04E-03 | 2,84 |
| C2 | 10450344 | NM_013484 | 1,10E-02 | 2,84 |
| Cd97 | 10580033 | NM_011925 | 3,29E-03 | 2,84 |
| Glis2 | 10433264 | NM_031184 | 6,83E-04 | 2,84 |
| Mmp3 | 10583071 | NM_010809 | 1,06E-02 | 2,84 |
| 1810010H24Rik | 10382136 | NM_001163473 | 8,03E-03 | 2,85 |
| Art3 | 10523231 | NM_181728 | 6,60E-03 | 2,85 |
| Ccrl2 | 10597279 | NM_017466 | 1,26E-03 | 2,85 |
| Colec12 | 10453747 | NM_130449 | 2,36E-03 | 2,85 |
| Fzd4 | 10554808 | NM_008055 | 5,62E-03 | 2,85 |
| Gm10484 | 10527963 | AK142929 | 7,11E-04 | 2,85 |
| Ndrg2 | 10419578 | NM_013864 | 2,36E-03 | 2,85 |
| Olfml2a | 10471882 | NM_172854 | 1,40E-03 | 2,85 |
| Timp4 | 10547022 | NM_080639 | 1,40E-03 | 2,85 |
| Clec4a1 | 10541555 | NM_199311 | 2,95E-02 | 2,86 |
| Gm10404 | 10412699 | AK077526 | 1,63E-02 | 2,86 |
| Plod2 | 10587829 | NM_001142916 | 6,29E-04 | 2,86 |
| Tph1 | 10563570 | NM_009414 | 2,52E-02 | 2,86 |
| BC067074 | 10407286 | ENSMUST00000078163 | 4,21E-03 | 2,87 |
| Gli3 | 10403727 | NM_008130 | 1,83E-03 | 2,87 |
| Slc44a4 | 10444554 | NM_023557 | 2,04E-03 | 2,87 |
| C230081A13Rik | 10593856 | NM_172924 | 2,77E-04 | 2,88 |
| Cxcl12 | 10541075 | NM_001012477 | 3,93E-03 | 2,88 |
| Meis2 | 10486041 | NM_001136072 | 9,87E-03 | 2,88 |
| Agtr1a | 10404376 | NM_177322 | 2,76E-02 | 2,89 |
| Nat8l | 10521168 | NM_001001985 | 3,56E-02 | 2,89 |
| Tagln2 | 10351825 | NM_178598 | 3,73E-03 | 2,89 |
| Armcx2 | 10606735 | NM_026139 | 1,07E-03 | 2,9 |
| Bmp2k | 10523468 | NM_080708 | 4,62E-04 | 2,9 |
| Crot | 10528102 | NM_023733 | 3,66E-04 | 2,9 |
| Duxbl | 10413243 | NM_183389 | 1,77E-03 | 2,9 |
| Rab32 | 10367822 | NM_026405 | 1,53E-03 | 2,9 |
| AI607873 | 10360377 | BC150711 | 4,27E-03 | 2,91 |
| Cd163 | 10541644 | NM_001170395 | 2,34E-02 | 2,91 |
| Ceacam20 | 10550740 | NM_027839 | 5,19E-04 | 2,91 |
| Cx3cl1 | 10574220 | NM_009142 | 2,13E-03 | 2,91 |
| Frem1 | 10514088 | NM_177863 | 2,46E-03 | 2,91 |
| Pmp22 | 10376950 | NM_008885 | 2,77E-03 | 2,91 |
| Thy1 | 10584628 | NM_009382 | 1,95E-02 | 2,91 |
| Fbln2 | 10540085 | NM_007992 | 3,10E-03 | 2,92 |
| Gadd45b | 10364950 | NM_008655 | 3,89E-03 | 2,92 |
| Antxr2 | 10531560 | NM_133738 | 6,11E-03 | 2,93 |
| Ear2 | 10414262 | NM_007895 | 3,09E-02 | 2,94 |
| Esm1 | 10407281 | NM_023612 | 5,45E-04 | 2,94 |
| Fut9 | 10511870 | NM_010243 | 1,82E-03 | 2,94 |
| Igf2 | 10569344 | NM_001122737 | 3,73E-04 | 2,94 |
| Loxl1 | 10594066 | NM_010729 | 2,05E-02 | 2,94 |
| Slc1a3 | 10427590 | NM_148938 | 4,34E-03 | 2,94 |
| Srpx2 | 10601659 | NM_026838 | 2,28E-02 | 2,94 |
| Abca1 | 10512949 | NM_013454 | 9,09E-04 | 2,95 |
| Cyp27a1 | 10347481 | NM_024264 | 9,00E-03 | 2,95 |
| Slc43a3 | 10473384 | NM_021398 | 5,22E-04 | 2,95 |
| 9530053A07Rik | 10551435 | NM_001164655 | 1,77E-03 | 2,96 |
| Antxr1 | 10545974 | NM_054041 | 4,00E-04 | 2,96 |
| Emcn | 10496359 | NM_001163522 | 4,11E-03 | 2,96 |
| Gm9911 | 10578017 | AK035883 | 1,53E-02 | 2,96 |
| Oasl2 | 10524621 | NM_011854 | 1,30E-02 | 2,96 |
| Peg3 | 10559796 | NM_008817 | 3,66E-04 | 2,96 |
| Zdhhc2 | 10571399 | NM_178395 | 1,89E-02 | 2,96 |
| Clip3 | 10551852 | NM_001081114 | 2,16E-03 | 2,97 |
| Dock11 | 10599120 | NM_001009947 | 6,69E-03 | 2,97 |
| Fam5c | 10350506 | NM_153539 | 1,31E-02 | 2,97 |
| Ltbp2 | 10401527 | NM_013589 | 4,93E-04 | 2,97 |
| Slc2a4 | 10387743 | NM_009204 | 1,90E-02 | 2,97 |
| Acsm5 | 10556701 | NM_178758 | 1,51E-02 | 2,98 |
| Prkar2b | 10399908 | NM_011158 | 2,35E-02 | 2,98 |
| Pdlim3 | 10571601 | NM_016798 | 3,58E-04 | 2,99 |
| Clec4a3 | 10541564 | NM_153197 | 1,24E-02 | 3 |
| Igsf10 | 10498379 | NM_001162884 | 1,01E-02 | 3 |
| Mlxipl | 10526277 | NM_021455 | 1,15E-02 | 3 |
| Gpx8 | 10412207 | NM_027127 | 8,60E-03 | 3,01 |
| Mir143 | 10459227 | NR_029601 | 6,39E-03 | 3,01 |
| Rcn3 | 10563077 | NM_026555 | 6,11E-03 | 3,01 |
| Tspan6 | 10606609 | NM_019656 | 7,83E-04 | 3,01 |
| Cdk14 | 10527940 | NM_011074 | 4,50E-04 | 3,02 |
| Cfd | 10364542 | NM_013459 | 7,68E-03 | 3,02 |
| Gpx3 | 10376201 | NM_001083929 | 2,18E-03 | 3,02 |
| Ifi205 | 10360406 | NM_172648 | 9,33E-03 | 3,02 |
| Lrrc17 | 10519998 | NM_028977 | 6,12E-03 | 3,02 |
| Pdgfrb | 10456046 | NM_001146268 | 2,27E-03 | 3,02 |
| Pdgfrl | 10571467 | NM_026840 | 5,16E-04 | 3,02 |
| Hoxc6 | 10427293 | NM_010465 | 3,03E-03 | 3,03 |
| Slc22a4 | 10385893 | NM_019687 | 8,28E-03 | 3,03 |
| Abca6 | 10392601 | NM_147218 | 6,02E-03 | 3,04 |
| Tcfap2b | 10345016 | NM_009334 | 2,66E-02 | 3,04 |
| Vcam1 | 10501608 | NM_011693 | 4,97E-04 | 3,04 |
| Crispld1 | 10344990 | NM_031402 | 8,37E-04 | 3,05 |
| Ifi204 | 10360382 | NM_008329 | 5,59E-03 | 3,05 |
| Itga7 | 10367440 | NM_008398 | 3,92E-02 | 3,05 |
| Slc43a1 | 10473367 | NM_001081349 | 1,01E-02 | 3,05 |
| Cdh6 | 10427862 | NM_007666 | 1,42E-03 | 3,06 |
| Ces3 | 10580635 | NM_053200 | 2,92E-02 | 3,06 |
| Cmklr1 | 10532711 | NM_008153 | 2,78E-02 | 3,06 |
| Mrc1 | 10469358 | NM_008625 | 1,11E-02 | 3,06 |
| Peg10 | 10536294 | NM_130877 | 5,22E-03 | 3,06 |
| Zfhx4 | 10490777 | NM_030708 | 8,97E-03 | 3,06 |
| 6330406I15Rik | 10527649 | BC116246 | 3,01E-03 | 3,07 |
| Abca8a | 10392522 | NM_153145 | 2,77E-04 | 3,07 |
| Mfap5 | 10541496 | NM_015776 | 1,27E-02 | 3,07 |
| Klb | 10522127 | NM_031180 | 1,17E-02 | 3,08 |
| Parm1 | 10523190 | NM_145562 | 1,98E-03 | 3,08 |
| Sgcb | 10530633 | NM_011890 | 1,34E-02 | 3,08 |
| Cd68 | 10387536 | NM_009853 | 1,11E-02 | 3,09 |
| Podn | 10514939 | NM_172874 | 8,26E-03 | 3,09 |
| Adrb3 | 10577973 | NM_013462 | 1,28E-02 | 3,1 |
| Cd209a | 10576784 | NM_133238 | 2,24E-02 | 3,1 |
| Ctnnd2 | 10423471 | NM_008729 | 1,72E-03 | 3,1 |
| Mxra8 | 10511180 | NM_024263 | 2,33E-03 | 3,1 |
| Bmp3 | 10523506 | NM_173404 | 5,65E-03 | 3,11 |
| Ccdc80 | 10435948 | NM_026439 | 1,16E-02 | 3,11 |
| Them5 | 10494016 | NM_025416 | 9,11E-04 | 3,11 |
| Zcchc24 | 10418171 | NM_001101433 | 8,58E-03 | 3,11 |
| Eepd1 | 10583920 | NM_026189 | 1,43E-02 | 3,12 |
| Plbd1 | 10548817 | NM_025806 | 1,20E-02 | 3,12 |
| Plin4 | 10451932 | NM_020568 | 1,99E-02 | 3,13 |
| Adra1a | 10416099 | NM_013461 | 5,32E-03 | 3,14 |
| Aoc3 | 10381371 | NM_009675 | 2,15E-02 | 3,14 |
| Dpt | 10351293 | NM_019759 | 9,73E-03 | 3,14 |
| Garnl3 | 10481772 | NM_178888 | 2,77E-04 | 3,14 |
| Acot7 | 10510687 | NM_133348 | 1,30E-03 | 3,15 |
| Bnc2 | 10514177 | NM_172870 | 1,85E-02 | 3,15 |
| Cpeb1 | 10565089 | NM_007755 | 3,69E-03 | 3,15 |
| 2900062L11Rik | 10601846 | NM_029823 | 3,38E-02 | 3,16 |
| Maob | 10603746 | NM_172778 | 9,18E-03 | 3,16 |
| Pdgfra | 10522503 | NM_011058 | 6,94E-03 | 3,16 |
| Samd9l | 10542911 | NM_010156 | 7,01E-03 | 3,16 |
| Cav2 | 10536494 | NM_016900 | 9,10E-04 | 3,17 |
| Cxcr4 | 10357472 | NM_009911 | 1,14E-03 | 3,17 |
| Dpep2 | 10581434 | NM_176913 | 4,55E-02 | 3,17 |
| Gpr133 | 10525989 | NM_001081342 | 5,00E-02 | 3,17 |
| Tmem159 | 10556820 | NM_145586 | 1,53E-03 | 3,17 |
| Enpep | 10502081 | NM_007934 | 3,14E-02 | 3,18 |
| Gstm1 | 10501229 | NM_010358 | 3,53E-03 | 3,19 |
| Serpinf1 | 10388430 | NM_011340 | 4,16E-03 | 3,19 |
| Aqp7 | 10512145 | NM_007473 | 1,20E-02 | 3,21 |
| Dpep1 | 10576235 | NM_007876 | 2,05E-03 | 3,21 |
| Emp3 | 10563441 | NM_010129 | 1,36E-02 | 3,21 |
| Itih5 | 10469151 | NM_172471 | 8,27E-03 | 3,21 |
| Gsn | 10471655 | NM_146120 | 4,56E-03 | 3,22 |
| Trpm3 | 10462039 | NM_001035244 | 1,27E-03 | 3,22 |
| Cacna1g | 10389929 | NM_009783 | 2,28E-03 | 3,23 |
| Htra1 | 10558150 | NM_019564 | 8,98E-03 | 3,23 |
| A4galt | 10430968 | NM_001170954 | 1,33E-03 | 3,24 |
| Angptl2 | 10471555 | NM_011923 | 6,50E-03 | 3,24 |
| Hspa12a | 10468746 | NM_175199 | 1,33E-02 | 3,24 |
| Il17b | 10456136 | NM_019508 | 2,87E-03 | 3,24 |
| Emb | 10407327 | NM_010330 | 5,65E-03 | 3,25 |
| Fgf2 | 10491699 | NM_008006 | 4,81E-02 | 3,25 |
| Phyh | 10469046 | NM_010726 | 8,60E-03 | 3,25 |
| Adam12 | 10568668 | NM_007400 | 3,74E-04 | 3,26 |
| Fbln7 | 10475932 | NM_024237 | 6,83E-04 | 3,26 |
| Gm3893 | 10504121 | BC059060 | 1,15E-03 | 3,26 |
| Htra3 | 10529485 | NM_030127 | 8,26E-03 | 3,26 |
| Pid1 | 10356172 | NM_001003948 | 5,91E-03 | 3,26 |
| AU018778 | 10580663 | NM_144930 | 4,03E-02 | 3,27 |
| Niacr1 | 10533720 | NM_030701 | 2,28E-02 | 3,27 |
| Pla2g7 | 10445293 | NM_013737 | 1,56E-04 | 3,27 |
| Dnase1l3 | 10417526 | NM_007870 | 1,44E-02 | 3,28 |
| Pla1a | 10439442 | NM_134102 | 1,18E-02 | 3,28 |
| Sgce | 10542965 | NM_001130190 | 8,47E-03 | 3,29 |
| Cidec | 10546929 | NM_178373 | 2,99E-02 | 3,3 |
| Gm10808 | 10435961 | ENSMUST00000099728 | 6,79E-03 | 3,3 |
| Maf | 10581992 | NM_001025577 | 3,48E-03 | 3,3 |
| Lgals3 | 10414360 | NM_001145953 | 7,45E-03 | 3,31 |
| Rufy4 | 10347282 | NM_001170641 | 1,41E-02 | 3,31 |
| Adamts12 | 10423109 | NM_175501 | 2,77E-03 | 3,32 |
| Cd3e | 10593024 | NM_007648 | 3,53E-02 | 3,32 |
| Fcgrt | 10563085 | NM_010189 | 1,99E-03 | 3,32 |
| Igfbp3 | 10384223 | NM_008343 | 3,74E-04 | 3,32 |
| Arhgef6 | 10604713 | NM_152801 | 8,00E-04 | 3,33 |
| Nr1h3 | 10484987 | NM_013839 | 2,13E-03 | 3,33 |
| Spib | 10562812 | NM_019866 | 9,21E-04 | 3,33 |
| Acss3 | 10372208 | NM_001142804 | 6,69E-03 | 3,34 |
| 2610528A11Rik | 10419034 | BC049685 | 4,18E-03 | 3,35 |
| Ephb3 | 10434559 | NM_010143 | 1,71E-03 | 3,35 |
| Cpxm1 | 10487645 | NM_019696 | 2,32E-02 | 3,36 |
| Lgi2 | 10530029 | NM_144945 | 1,90E-02 | 3,36 |
| Negr1 | 10497051 | NM_001039094 | 1,98E-02 | 3,36 |
| Etv1 | 10395457 | NM_007960 | 1,18E-03 | 3,37 |
| St6galnac5 | 10502881 | NM_012028 | 2,21E-02 | 3,37 |
| Olfr550 | 10555722 | NM_147104 | 3,52E-03 | 3,39 |
| Rasgef1c | 10375650 | NM_029004 | 1,56E-04 | 3,39 |
| Scara5 | 10416023 | NM_028903 | 2,42E-03 | 3,39 |
| Slc4a4 | 10523021 | NM_018760 | 1,36E-03 | 3,39 |
| Adam23 | 10346882 | NM_011780 | 1,11E-02 | 3,4 |
| Il13ra1 | 10599174 | NM_133990 | 5,92E-04 | 3,4 |
| Svep1 | 10513208 | NM_022814 | 8,70E-03 | 3,4 |
| Zeb2 | 10482448 | NM_015753 | 7,11E-04 | 3,4 |
| Atp1a2 | 10360270 | NM_178405 | 6,05E-03 | 3,41 |
| Nkd2 | 10410547 | NM_028186 | 4,50E-04 | 3,41 |
| Plac8 | 10531724 | NM_139198 | 3,89E-03 | 3,41 |
| Pltp | 10489569 | NM_011125 | 9,35E-04 | 3,42 |
| Epha7 | 10503659 | NM_010141 | 2,45E-03 | 3,43 |
| H6pd | 10518743 | NM_173371 | 1,21E-02 | 3,43 |
| Sod3 | 10521824 | NM_011435 | 2,60E-02 | 3,43 |
| Tnxb | 10444459 | NM_031176 | 3,06E-02 | 3,43 |
| Cxcl13 | 10523359 | NM_018866 | 2,38E-02 | 3,44 |
| Fry | 10527732 | NM_172887 | 1,23E-02 | 3,44 |
| Myb | 10368199 | NM_010848 | 1,40E-03 | 3,44 |
| Ntrk2 | 10405633 | NM_001025074 | 1,29E-02 | 3,44 |
| Wfdc2 | 10478525 | NM_026323 | 6,71E-04 | 3,44 |
| Anxa8 | 10414065 | NM_013473 | 6,03E-04 | 3,45 |
| Ltc4s | 10385583 | NM_008521 | 3,64E-03 | 3,47 |
| Apod | 10439009 | NM_007470 | 1,57E-03 | 3,48 |
| Ccdc129 | 10538503 | NM_001081665 | 7,82E-04 | 3,48 |
| Gstt1 | 10370000 | NM_008185 | 3,01E-03 | 3,48 |
| Cyp2d22 | 10430851 | NM_001163472 | 2,07E-03 | 3,49 |
| Tmem45b | 10592044 | NM_144936 | 1,20E-02 | 3,49 |
| Twist2 | 10348632 | NM_007855 | 5,32E-03 | 3,49 |
| Acvr1c | 10482814 | NM_001111030 | 1,33E-02 | 3,5 |
| Adh1 | 10496438 | NM_007409 | 1,32E-02 | 3,5 |
| Cdo1 | 10458828 | NM_033037 | 2,29E-02 | 3,5 |
| Lrp1 | 10373223 | NM_008512 | 2,74E-03 | 3,5 |
| C1rb | 10541683 | NM_001113356 | 7,75E-03 | 3,51 |
| Dmrt2 | 10462231 | NM_145831 | 4,02E-02 | 3,51 |
| Hmgcs2 | 10494643 | NM_008256 | 8,37E-04 | 3,51 |
| Lrg1 | 10451953 | NM_029796 | 2,03E-02 | 3,51 |
| Ogn | 10405063 | NM_008760 | 8,76E-03 | 3,51 |
| Sgcd | 10385466 | NM_011891 | 4,90E-03 | 3,51 |
| 9530091C08Rik | 10586718 | NR_033299 | 1,14E-02 | 3,52 |
| Nova1 | 10400157 | NM_021361 | 1,19E-02 | 3,52 |
| Adipoq | 10434747 | NM_009605 | 2,15E-02 | 3,53 |
| Fmo1 | 10359571 | NM_010231 | 5,28E-03 | 3,53 |
| Hsd11b1 | 10361234 | NM_008288 | 1,74E-02 | 3,53 |
| Mrap | 10436804 | NM_029844 | 3,60E-02 | 3,53 |
| Amy1 | 10501555 | NM_007446 | 2,58E-02 | 3,55 |
| Fxyd1 | 10562211 | NM_052992 | 3,30E-03 | 3,55 |
| Prrx1 | 10359624 | NM_175686 | 4,04E-03 | 3,55 |
| Serpina3c | 10402435 | NM_008458 | 1,44E-02 | 3,55 |
| Cacng7 | 10569714 | NM_133189 | 3,25E-03 | 3,57 |
| Mmp19 | 10367400 | NM_021412 | 1,22E-02 | 3,57 |
| Dram1 | 10371607 | NM_027878 | 1,31E-02 | 3,58 |
| Rarres2 | 10544573 | NM_027852 | 3,00E-03 | 3,58 |
| Igf1 | 10365559 | NM_010512 | 3,22E-03 | 3,59 |
| Cfh | 10358339 | NM_009888 | 6,59E-03 | 3,6 |
| Slc1a5 | 10550332 | NM_009201 | 9,05E-03 | 3,6 |
| Dapp1 | 10502359 | NM_011932 | 2,77E-04 | 3,63 |
| Pgf | 10401607 | NM_008827 | 1,26E-03 | 3,64 |
| Prkcdbp | 10566454 | NM_028444 | 1,60E-03 | 3,64 |
| Lep | 10536789 | NM_008493 | 2,15E-02 | 3,65 |
| Lox | 10458894 | NM_010728 | 3,41E-03 | 3,65 |
| Spon1 | 10556509 | NM_145584 | 3,85E-03 | 3,65 |
| Cmbl | 10423505 | NM_181588 | 1,01E-02 | 3,66 |
| Enpp2 | 10428619 | NM_015744 | 3,66E-03 | 3,66 |
| Mmd | 10380289 | NM_026178 | 2,55E-02 | 3,66 |
| Ntng1 | 10501468 | NM_030699 | 1,26E-04 | 3,66 |
| Tmeff2 | 10346150 | NM_019790 | 3,66E-03 | 3,66 |
| Cited1 | 10606083 | NM_007709 | 2,24E-03 | 3,67 |
| Entpd8 | 10469923 | NM_028093 | 2,19E-04 | 3,67 |
| Gpc3 | 10604576 | NM_016697 | 2,82E-02 | 3,67 |
| Itga2 | 10412267 | NM_008396 | 3,09E-04 | 3,68 |
| Reg3g | 10545569 | NM_011260 | 2,49E-02 | 3,68 |
| Cdh11 | 10581013 | NM_009866 | 5,49E-03 | 3,69 |
| Chrdl1 | 10607124 | NM_001114385 | 6,03E-04 | 3,69 |
| Ggt5 | 10364072 | NM_011820 | 2,04E-03 | 3,69 |
| Mirlet7c-1 | 10436602 | NR_029728 | 1,12E-03 | 3,69 |
| Bche | 10498710 | NM_009738 | 8,54E-03 | 3,71 |
| Pck1 | 10479047 | NM_011044 | 4,76E-02 | 3,71 |
| Slc22a3 | 10447773 | NM_011395 | 6,98E-03 | 3,71 |
| Dhdh | 10563314 | NM_027903 | 1,28E-02 | 3,73 |
| Mmp14 | 10415052 | NM_008608 | 6,83E-04 | 3,73 |
| Mmp11 | 10370037 | NM_008606 | 7,82E-04 | 3,74 |
| C4b | 10450242 | NM_009780 | 2,27E-02 | 3,75 |
| Apcdd1 | 10456184 | NM_133237 | 1,02E-02 | 3,76 |
| Nnmt | 10593219 | NM_010924 | 6,94E-03 | 3,76 |
| Serping1 | 10484463 | NM_009776 | 3,26E-03 | 3,76 |
| Gm10115 | 10479973 | ENSMUST00000076071 | 5,25E-03 | 3,77 |
| Hey1 | 10497203 | NM_010423 | 8,48E-04 | 3,77 |
| Nrg4 | 10593776 | NM_032002 | 3,84E-02 | 3,77 |
| Figf | 10603099 | NM_010216 | 5,42E-03 | 3,79 |
| Foxa1 | 10400504 | NM_008259 | 3,73E-04 | 3,79 |
| Pde3b | 10556528 | NM_011055 | 2,03E-02 | 3,79 |
| Ly6i | 10429560 | NM_020498 | 1,68E-02 | 3,8 |
| Nnat | 10477986 | NM_010923 | 2,09E-02 | 3,8 |
| Adamts2 | 10375751 | NM_175643 | 6,71E-03 | 3,81 |
| Slc13a2 | 10388834 | NM_022411 | 1,79E-03 | 3,82 |
| A530053G22Rik | 10545079 | NR_015565 | 9,82E-03 | 3,84 |
| Hoxc8 | 10427290 | NM_010466 | 6,26E-03 | 3,85 |
| Capn6 | 10607143 | NM_007603 | 4,82E-03 | 3,86 |
| Sgms2 | 10502224 | NM_028943 | 3,85E-03 | 3,86 |
| Lrrc32 | 10555174 | NM_001113379 | 1,10E-03 | 3,87 |
| Abi3bp | 10436304 | NM_001014423 | 1,36E-03 | 3,88 |
| C7 | 10427436 | ENSMUST00000110689 | 1,46E-02 | 3,89 |
| Egr2 | 10363735 | NM_010118 | 2,21E-03 | 3,89 |
| Irs3 | 10534883 | NM_010571 | 4,87E-03 | 3,9 |
| Ptprd | 10513957 | NM_011211 | 1,18E-02 | 3,9 |
| Aldh1a3 | 10564417 | NM_053080 | 2,06E-03 | 3,91 |
| Clca1 | 10502552 | NM_009899 | 3,30E-03 | 3,92 |
| Cd44 | 10485405 | NM_009851 | 2,25E-04 | 3,93 |
| Ifi27l2a | 10402347 | NM_029803 | 5,17E-03 | 3,93 |
| Sned1 | 10348739 | NM_172463 | 3,09E-04 | 3,94 |
| A430107O13Rik | 10536635 | BC151018 | 2,27E-03 | 3,95 |
| Pamr1 | 10474129 | NM_173749 | 1,14E-02 | 3,97 |
| Mmp2 | 10573924 | NM_008610 | 1,95E-03 | 3,99 |
| Mc2r | 10459611 | NM_008560 | 1,77E-02 | 4,01 |
| Plin1 | 10564795 | NM_175640 | 2,84E-02 | 4,04 |
| Plagl1 | 10361771 | NM_009538 | 1,02E-03 | 4,05 |
| Rbp4 | 10467319 | NM_001159487 | 3,57E-02 | 4,06 |
| Slit3 | 10375175 | NM_011412 | 2,45E-03 | 4,08 |
| Srpx | 10603583 | NM_016911 | 5,93E-03 | 4,08 |
| A530016L24Rik | 10398824 | NM_177039 | 4,08E-02 | 4,09 |
| Cyp2e1 | 10558673 | NM_021282 | 7,30E-03 | 4,1 |
| Clca2 | 10502565 | NM_030601 | 2,75E-03 | 4,12 |
| Rftn1 | 10451710 | ENSMUST00000044503 | 8,81E-05 | 4,14 |
| Mir99a | 10436600 | NR_029535 | 8,23E-05 | 4,17 |
| Slit2 | 10521759 | NM_178804 | 3,31E-04 | 4,18 |
| Bace2 | 10437210 | NM_019517 | 5,44E-04 | 4,19 |
| Slc7a10 | 10552143 | NM_017394 | 3,96E-03 | 4,19 |
| Tshr | 10397606 | NM_011648 | 5,72E-03 | 4,19 |
| Mir181b-2 | 10471880 | NR_029904 | 1,11E-02 | 4,2 |
| Efemp1 | 10374777 | NM_146015 | 5,67E-03 | 4,21 |
| Vnn1 | 10362138 | NM_011704 | 1,55E-02 | 4,21 |
| F830001A07Rik | 10586722 | AK089550 | 9,62E-03 | 4,22 |
| Smoc1 | 10396936 | NM_001146217 | 8,50E-03 | 4,23 |
| Abcd2 | 10431697 | NM_011994 | 1,54E-02 | 4,25 |
| Hc | 10481962 | NM_010406 | 1,00E-02 | 4,25 |
| Rarres1 | 10498584 | NM_001164763 | 2,77E-03 | 4,25 |
| Adamts18 | 10581961 | NM_172466 | 5,16E-04 | 4,27 |
| Fgf10 | 10407350 | NM_008002 | 7,00E-03 | 4,29 |
| Hpgd | 10571840 | NM_008278 | 2,57E-03 | 4,29 |
| Glb1l2 | 10591884 | NM_153803 | 1,05E-02 | 4,31 |
| Aebp1 | 10374083 | NM_009636 | 2,04E-03 | 4,32 |
| B3galt2 | 10350473 | NM_020025 | 6,96E-03 | 4,36 |
| Slc36a2 | 10386020 | NM_153170 | 1,41E-02 | 4,36 |
| Thbs2 | 10447951 | NM_011581 | 1,48E-03 | 4,36 |
| Mfap2 | 10509901 | NM_008546 | 3,48E-03 | 4,39 |
| Agt | 10582658 | NM_007428 | 6,47E-03 | 4,4 |
| Gpr81 | 10533725 | NM_175520 | 1,21E-02 | 4,43 |
| Inmt | 10544932 | NM_009349 | 5,35E-03 | 4,43 |
| Serpina3n | 10398075 | NM_009252 | 1,68E-02 | 4,43 |
| Fn1 | 10355403 | NM_010233 | 1,86E-02 | 4,44 |
| Sncg | 10418921 | NM_011430 | 1,42E-02 | 4,53 |
| Prelp | 10357870 | NM_054077 | 9,15E-03 | 4,54 |
| Foxi1 | 10385114 | NM_023907 | 3,14E-04 | 4,84 |
| Ccl19 | 10504132 | NM_011888 | 5,22E-04 | 4,9 |
| Lect1 | 10421853 | NM_010701 | 2,37E-04 | 5,11 |
| Bhlhe41 | 10549276 | NM_024469 | 8,32E-04 | 5,35 |
| Zim1 | 10559790 | NM_011769 | 1,57E-04 | 5,37 |
| Mcpt4 | 10420247 | NM_010779 | 2,29E-03 | 5,66 |
| Scg3 | 10595033 | NM_009130 | 6,12E-04 | 5,66 |
| St8sia6 | 10480238 | NM_145838 | 3,49E-04 | 5,91 |
| Tnfaip2 | 10398665 | NM_009396 | 6,32E-04 | 6,24 |
| Gabrp | 10385083 | NM_146017 | 1,03E-04 | 6,84 |
| Chi3l1 | 10349968 | NM_007695 | 2,37E-04 | 6,99 |
| Gm266 | 10402705 | NM_001033248 | 1,48E-03 | 7,02 |
| Hp | 10581605 | NM_017370 | 5,86E-03 | 7,08 |
| Clic6 | 10436958 | NM_172469 | 2,11E-04 | 7,21 |
| Penk | 10511363 | NM_001002927 | 2,36E-03 | 8,16 |
| Alb | 10523062 | NM_009654 | 1,18E-02 | 8,37 |
| Gpnmb | 10538187 | NM_053110 | 3,67E-02 | 8,37 |
| Cpa3 | 10497451 | NM_007753 | 5,35E-03 | 8,67 |
| Fbn2 | 10458999 | NM_010181 | 2,25E-04 | 10,49 |
| Cybrd1 | 10472757 | NM_028593 | 2,19E-04 | 11 |
| Alox12e | 10387838 | NM_145684 | 2,77E-04 | 11,7 |
| Slco1a5 | 10549041 | NM_130861 | 8,81E-05 | 11,75 |
| Atp6v1b1 | 10539449 | NM_134157 | 2,39E-04 | 11,82 |
| Msln | 10449000 | NM_018857 | 2,43E-04 | 18,19 |
| C530030P08Rik | 10375123 | ENSMUST00000101381 | 8,23E-05 | 31,48 |
|  |  |  |  |  |
