# Supplementary material for: Phenotypic and Molecular Alterations in the Mammary Tissue of R-Spondin1 Knock-Out Mice during Pregnancy
Source: PLoS One. 2016 Sep 9;11(9):e0162566. doi: 10.1371/journal.pone.0162566 (PMC5017653; doi:10.1371/journal.pone.0162566)
Supplement: S5 Table — (DOCX) [file pone.0162566.s006.docx]

**Supplemental Table 5:** RT-qPCR of 10 genes selected among microarrays data.

|  | **P12** | | **P16** | |
| --- | --- | --- | --- | --- |
|  | qPCR | arrays | qPCR | arrays |
| *Csn1s2a* | -40.22 | -1.91 | -5.58 | nd |
| *Fabp3* | -50.21 | -30.39 | -5.98 | -11.02 |
| *Gjb6* | -56.49 | -31.49 | -2.71 | -44.83 |
| *Igfbp7* | 2.97 | nd | 1.35 | 2.19 |
| *Lect1* | 42.52 | 8.65 | 3.36 | 5.11 |
| *Mtmr9* | 5.54 | 1.96 | 8.00 | 1.6 |
| *Olah* | -14.42 | -13.54 | -6.15 | -14.4 |
| *Rspo1* | -15.35 | -4.93 | -21.26 | -2.68 |
| *Sparc* | 2.64 | nd | 13.74 | 1.75 |
| *Vcan* | 6.36 | 3.01 | 10.78 | 2.57 |

nd : not determined
