# Supplementary material for: Phenotypic and Molecular Alterations in the Mammary Tissue of R-Spondin1 Knock-Out Mice during Pregnancy
Source: PLoS One. 2016 Sep 9;11(9):e0162566. doi: 10.1371/journal.pone.0162566 (PMC5017653; doi:10.1371/journal.pone.0162566)
Supplement: S6 Table — (DOCX) [file pone.0162566.s007.docx]

**Supplemental Table 6**: **Ingenuity Pathway Analysis- Top Functions associated with *Rspo1* inactivation at pregnancy day-12.**

| Top Network | Score | Genes in Network |  |  |
| --- | --- | --- | --- | --- |
|  |  | **Upregulated** | **Downregulated** |  |
| Cellular Movement, Cardiovascular System Development and Function, Organ Morphology | 45 | ADAM12,BCAT1,CRABP1,EMB,ESM1,ETV4,HEY1,HEY2,HPGD,PAPPA,  S100A6,SGK223,STRA6,TRPV6,VCAN | APLN,BCL2L14,CRABP2,GJB2,GJB6,  NUCB2,RGS16,SEMA4D,ST6GAL1,  TGFB2 |  |
| Cellular Assembly and Organization, Cellular Function and Maintenance, Connective Tissue Disorders | 38 | ADAMTS14,BMP3,CDH2,COL1A1,  COL1A2,COL3A1,COL5A2,CTNND2,  DSC3,DSP,EPHB3,INHBB,LECT1,  MTMR7,MYB,PLOD2,SLIT2,WFDC2 | HLF,LRRC7,MUC4,TMPRSS4 |  |
| Cellular Function and Maintenance, Hematological System Development and Function, Cell Death and Survival | 35 | ALDH1A3,DSG1,GADD45B,SPIB,  TNFAIP2 | ANKRD22,ARHGDIG,CARD14,CD7,  CD82,CITED4,FAM46C,Gzmb,  IL18R1,KCNN4,PIGR,PTPN22,RAB4A,SLC5A1,TFRC,TNFRSF19 |  |
| Cancer, Endocrine System Disorders, Organismal Injury and Abnormalities | 35 | AREG,CAPN6,CAPN8,CDK14,CITED1,  EGR2,GJA1,MSLN,RGS2,SLCO1A2 | CA2,CALML3,CBX7,Elf5,FABP3,GOT1,ICA1,MAPK4,SPP1,SYT9,Wap |  |
| Cancer, Endocrine System Disorders, Gastrointestinal Disease | 29 | CACNA1G,FOXA1,HMCN1,HP,KRT7,  SLC5A8,UGT8 | ATP7B,BAIAP2L1,BHLHA15,CYTIP,  DUOX1,GLDC,NPNT,PGLYRP1,  RNASE1,STAT5A,TNIK |  |
| Cancer, Gastrointestinal Disease, Organismal Injury and Abnormalities | 27 | ANXA8/ANXA8L1,FUT9,GNA14,  NTNG1 | BSPRY,CA6,DUOXA1,FABP3,FKBP11,GLDC,IGSF5,MAPK4,RGS8,ROGDI,  SLC16A12,Timd2,TRIM7 |  |
| Cardiovascular System Development and Function, Cell Morphology, Skeletal and Muscular System Development and Function | 25 | Bhlhe41,ELOVL4,MOXD1,NKD2,  POF1B,RASGEF1C,ST8SIA6,SV2B | AASS,ATP2C2,BAIAP2L1,CCDC64B,  MYO5C,SH3YL1,TMPRSS13,UPB1 |  |
| Cancer, Cell Morphology, Cellular Assembly and Organization | 22 | ARHGAP40,CCDC141,CTNND2,IGSF9,MDGA2,SLC35F3,SLC6A15,TRPV6 | GOT1,HHIPL2,KCTD14,LIPH,MEIG1,  PLCXD2,SLC9A4 |  |
| Embryonic Development, Hair and Skin Development and Function, Organ Development | 20 | ADAMTS15,Alox12e,CSMD1,ITIH2 | EHHADH,ELOVL7,FAM189A2,  MAST4,MUC20,OLAH,Scgb1c1,  SCRG1,SLC6A14,Wap |  |
| Cellular Growth and Proliferation, Cellular Movement, Embryonic Development | 20 | BACE2,CYBRD1,FBN2,THEM5 | ATP6V1C2,CHRDL2,CLDN8,Csn1s2b,FAM20C,KCNK6,RSPO1,STX19,TC2N,Wap |  |
| Cell-To-Cell Signaling and Interaction, Cellular Function and Maintenance, Nervous System Development and Function | 19 | BASP1,GIPC2,PTN,SLC13A2,TRPM3 | ACSL4,APOBEC3B,DUOX1,FOLR1,  OVOL1,PIK3C2G,SLC38A3,Sult1d1 |  |
| Molecular Transport, Small Molecule Biochemistry, Cell Death and Survival | 19 | CLIC6,FOXI1,PKD1L1,R3HDML | Apol7e (includes others),  FABP3,FAM20A,FN3K,GPR110,GRHL1,NAALADL2,Scd3,SUSD4 |  |
