# Supplementary material for: Phenotypic and Molecular Alterations in the Mammary Tissue of R-Spondin1 Knock-Out Mice during Pregnancy
Source: PLoS One. 2016 Sep 9;11(9):e0162566. doi: 10.1371/journal.pone.0162566 (PMC5017653; doi:10.1371/journal.pone.0162566)
Supplement: S7 Table — (DOCX) [file pone.0162566.s008.docx]

**Supplemental Table 7: Ingenuity Pathway Analysis- Top Functions associated with *Rspo1* inactivation at pregnancy day-16.**

| Top Network | Score | | Genes in Network | |  | |  |
| --- | --- | --- | --- | --- | --- | --- | --- |
|  |  | | Upregulated | | Downregulated | |  |
| Cancer, Organismal Injury and Abnormalities, Reproductive System Disease | 35 | | ACY3,AEBP1,AOC3,ASPA,CELF2,EMB,  EMP3,FBLN1,FBLN7,FBN2,HOXC8,  LAMC3,LGMN,MDK,MFAP5,NID1,  PLAGL1,PMP22,SHC2,SLC13A2,  SLC43A3,TMPRSS6 | | ACY1,ASS1,FOLR1,TDGF1,TPD52,TPD52L1 | |  |
| Carbohydrate Metabolism, Small Molecule Biochemistry, Hereditary Disorder | 35 | | Akr1c14,ANGPTL2,BMP2K,BMPER,CDC14A,  CHST1,CHST12,CPEB1,CRABP1,CYGB,EMP1,  EMP2,ESM1,FIGF,HMGCS2,JAM2,NDP,  PLXNA2,SLC16A7,SOD3,TSPAN12,UST,  VASH2 | | BTN1A1,CHRNB2,DUOX1,GJB2,HS3ST3B1,  HS6ST1,Sult1d1,TBC1D8 | |  |
| Cell-To-Cell Signaling and Interaction, Cell Signaling, Hereditary Disorder | 35 | | ADCYAP1R1,ADRB3,BAI2,CALCRL,CCRL2,  CD97,CELSR1,CMKLR1,CYSLTR1,ELTD1,  EMR1,FZD4,GPR64,GPR124,GPR133,  GPR153,GPR137B,HTR1D,HTR2A,LPHN3,  MC2R,NPY1R,P2RY6,P2RY13,P2RY14,  PTGFR,RAMP2,SCTR | | DRD4,GPR110,OPN1LW | |  |
| Carbohydrate Metabolism, Post-Translational Modification, Cell-To-Cell Signaling and Interaction | 35 | | BCAT1,CDK14,ITGA4,LAPTM5,LDHB,  Mcpt4,PFKFB1,PFKFB3,PICALM,  PPAP2B,STOM,TAGLN2 | | CTNNAL1,DAB2,EHHADH,GALNT3,IARS,  NHP2,PFKFB2,PFKL,PYCR1,SEC23B,  SEC61B,SERP1,SND1,SRM,TRIM55,  TSTA3,UBA5 | |  |
| Cancer, Organismal Injury and Abnormalities, Reproductive System Disease | 31 | | ABCC1,ABLIM1,ALB,ANGPT2,ARMCX2,  BEGAIN,ETV5,FADS3,FKBP10,FXYD5,KRT7,  LRP1,MEOX2,MMP3,MMP14,NNMT,PLAU,  PQLC3,SYNPO,TRHDE,UGT8,  UGT1A9 (includes others) | | CLDN7,KRT15,LTF,QSOX1,ST14,TFRC | |  |
| Cellular Development, Cellular Growth and Proliferation, Lymphoid Tissue Structure and Development | 31 | | AR,Bex1,CAV1,CDH2,DISP2,FAM198B,GJA1,  Oasl2,PEG3,RCN3,SGMS2,SLC43A1,  TBXAS1,USP11 | | ACBD7,ATP13A4,Ccdc74a,DHODH,FKBP1,  MGAT2,OLAH,PCTP,PSTPIP2,SEC11C,  SLC26A7,SLC7A4,STEAP1,XBP1 | |  |
| Gene Expression, RNA Post-Transcriptional Modification, Cell-To-Cell Signaling and Interaction | 29 | | ABAT,ATP2B4,CAV2,Cd33,COLEC12,Cxcl15,  ECM1,GAS6,IFIT1B,IGF1R,MSR1,PCSK5,  PRKCDBP,PTRF,SCARA3,SCARA5,SDPR,TGFBI | | ATP2B2,ATP2C1,ATP2C2,COBL,Elf5,ORAI,  PCSK7,RAB18,STIM2,Wap | |  |
| Cancer, Embryonic Development, Tissue Morphology | 29 | | ADAMTS12,AKR1B10,ALDH1A1,ALDH1A3,  ALDH3B1,CDH6,CFD,CTDSPL,DCLK1,HOXB3,  IDH1,LOXL3,MAF,MAFB,NUAK1,SLCO3A1,  SNAI1,ST3GAL2,STEAP4,TNFAIP2,TWIST2,  ZDHHC2,ZEB1,ZEB2 | | ALDH18A1,C19orf10,ESRP2,TTC25 | |  |
| Embryonic Development, Organismal Development, Cellular Development | 27 | | BMP2,BMP3,BMPR1B,DPYSL2,DPYSL3,ENG,  FGF10,FLRT2,GDF10,GLI3,GLIS2,HEY1,  HOXC9,LECT1,MGP,OGN,PCDH18,RUNX2,  SIX1,SRGAP1,ZNF521,ZNF354C | | BAIAP2L1,CLCN3,CREB3L1 | |  |
| Cardiovascular System Development and Function, Cellular Development, Organismal Development | 27 | | AGT,APOE,CD44,CD200,CD3E,EGR1,EGR2,  FBXO21,FGF2,GADD45B,ITGB3,KIRREL,  LILRB4,LIMS2,MMP2,MOGAT1,MR1,  PDGFRL,PEG10,PRKCA,PRKCE,RGS12,  SLC10A6,VCAM1 | | IL18R1,KCNK5,ROGDI | |  |
| Carbohydrate Metabolism, Gastrointestinal Disease, Hepatic System Disease | 25 | | ACE,B4GALT5,CLIP3,Cyp2j9,DOCK6,EMCN,  ETV4,GEM,KLB,NDRG2,PARM1,PID1,  PLAC1,PLAC8,TACSTD2,TTC3,WDFY3 | | B4GALT1,BTN2A2,LALBA,Muc1,Muc4,  PDGFD,PROK1,SEC14L2,TMPRSS4 | |  |
| Lipid Metabolism, Small Molecule Biochemistry, Connective Tissue Development and Function | 25 | | ADH1C,APBB1IP,CDO1,CIDEC,CLEC4M,  CPNE8,DHRS3,DOCK11,EDIL3,GAS7,H6PD,  INMT,LAMA2,LAMA4,LIPE,PLIN1,PRRX1,  RARRES2,SOX8,SOX18,VNN1,VWF | | ABHD5,CEL,CIDEA,F5 | |  |
| Developmental Disorder, Hereditary Disorder, Immunological Disease | | 25 | | C2,C5,C7,C1QB,C1QC,C1R,C4A/C4B,CFH,  CFP,DNASE1L3,ITGA11,ITGAX,ITGB2,  LUM,PRELP,SERPING1,Siglech,SLC1A5,  TFPI,TYROBP,WFDC2 | | RADIL,RBM47,SFTPD,SLC39A7,SUSD4 | |
| Cancer, Organismal Injury and Abnormalities, Reproductive System Disease | | 25 | | ADAMTS2,ADAMTS15,APOD,CDH11,  CTNND2,DDR2,DPEP1,DPT,ECM2,Hmgn3,  HTRA1,METTL7A,MME,PAPLN,RCN1,  SERPINA1,SERPINH1,SPARCL1,  TPSAB1/TPSB2 | | ATP12A,GZMA,Gzmb,HSD17B2,IMPDH1,  SLC16A1,SSR4 | |
| Cardiovascular System Development and Function, Embryonic Development, Organ Development | | 25 | | CD34,FHL1,HES1,HEY2,ID3,JAG1,LMO1,  mir-143,MLXIPL,NOTCH1,NR2F2,NRARP,  NUMBL,PLXDC2,RUNX1T1,SGK223,SLFN5,  TAF9B,TCF4,WIF1 | | MLX,PROX1,RNF43,RNF128,RSPO1,  SLC5A5 | |
| Cellular Movement, Immune Cell Trafficking, Cellular Function and Maintenance | | 25 | | ABCC3,ADIPOQ,BGN,C10orf54,CASC4,  CCL11,CCL19,CSF1R,CX3CL1,CXCL13,CXCR4,  ENPP2,EPS8,F3,FCER1G,HSPB1,IL34,NKD2,  PCDH7,PF4 | | CCL28,KCNN4,RNASE1,SLC27A4,SLC34A2,  VDR | |
| Cellular Assembly and Organization, Cellular Function and Maintenance, Connective Tissue Disorders | | 25 | | ADAMTS14,BCAM,CDR2,COL18A1,COL1A1,  COL1A2,COL3A1,COL4A2,DCN,EFEMP2,  FAP,HSD11B1,IFFO1,LOX,mir-218,NDN,  NT5E,PLAT,PLOD1,PLOD2,PLSCR4,  RAP1GAP,SCNN1B,SERPINF1,THBS1,WISP1 | |  | |
| Lipid Metabolism, Small Molecule Biochemistry, Connective Tissue Development and Function | | 25 | | Acp5,AGPAT9,AGTR1,CSMD1,ETV1,Ggta1,  GPNMB,MEIS2,MITF,NOVA1,PIR,PLA1A,  PRKD1,RABEP1,SH2B2,SLC1A3,TNFRSF1B | | AGPAT6,APLN,CMTM6,FAM63B,  HSD17B7,KCNK1,KIAA1598,TNFRSF11A,  XDH | |
